# Cembrane- and Casbane-Type Diterpenes from the South China Sea Soft Coral *Sinularia nanolobata*

**DOI:** 10.3390/md24090317

**Published:** 2026-09-10

**Authors:** Yao Dong, Xiao Xiao, Li-Gong Yao, Lin-Fu Liang, Song-Wei Li, Yue-Wei Guo

**Affiliations:** 1 School of Medicine, Shanghai University, Shanghai 200444, China; 18788380886@163.com (Y.D.); xiao_xiao0121@163.com (X.X.); 2 Shandong Laboratory of Yantai Drug Discovery, Bohai Rim Advanced Research Institute for Drug Discovery, Yantai 264117, China; yaoligong@simm.ac.cn; 3 School of Chemistry and Chemical Engineering, Central South University of Forestry and Technology, Changsha 410004, China

**Keywords:** soft coral, *Sinularia nanolobata*, cembrane, casbane, structural elucidation

## Abstract

Five new cembrane-type diterpenes (**1**–**5**), one new casbane-type diterpene (**7**), and ten known related ones (**6**, **8**–**16**) were isolated from the South China Sea soft coral *Sinularia nanolobata*. The structures of new compounds were elucidated through comprehensive spectroscopic analysis, NMR calculation with DP4+ probability analysis, time-dependent density functional theory–electronic circular dichroism (TDDFT-ECD) calculations, and comparison with the reported spectroscopic data of known analogs. Structurally, compounds **2**–**5** featured rare five- to seven-membered oxygen-containing heterocyclic rings with diverse joints, reflecting remarkable chemical diversity of secondary metabolites. In bioassays, all isolates were evaluated for their cytotoxic and anti-inflammatory effects. Although no significant cytotoxic or anti-inflammatory activity was observed, the structural novelty of these compounds expands the chemical diversity of this class of natural products, providing valuable leads for further structural modifications and biological investigations.

## 1. Introduction

Marine organisms, especially invertebrates, are shaped by their complex and competitive environments, leading them to biosynthesize an enormous diversity of secondary metabolites with unique chemical scaffolds and potent biological activities [1]. Soft corals of the genus *Sinularia* (phylum Cnidaria, class Anthozoa, subclass Octocorallia, order Alcyonacea, family Alcyoniidae) stand out as particularly rich source of such marine natural products and serve as an amazing treasure trove of novel compounds ranging from sesquiterpenes, diterpenes, steroids, alkaloids to other miscellaneous compounds [2,3,4]. Numerous chemical investigations of the genus *Sinularia* revealed a vast array of structurally diverse secondary metabolites displayed highly promising biological activities including antibacterial, osteoclast-inhibitory, anti-inflammatory, antithrombotic, antiviral, cytotoxic, and immunosuppressive activities [4,5].

Our team has been continuously dedicated to the search for bioactive secondary metabolites with novel structures from marine invertebrates, particularly soft corals from the South China Sea, and a large quantity of secondary metabolites with structural diversity and interesting bioactivities have been isolated and characterized [6,7,8]. Previously, an unprecedented dieterpene with a tricyclo[10.3.0.0^1,2^]pentadecane carbon skeleton and multiple polyoxygenated and unusual endoperoxide-bridged casbane-type diterpenes were found from *S. nanolobata* collected off Ximao Island [9].

**Figure 1:**
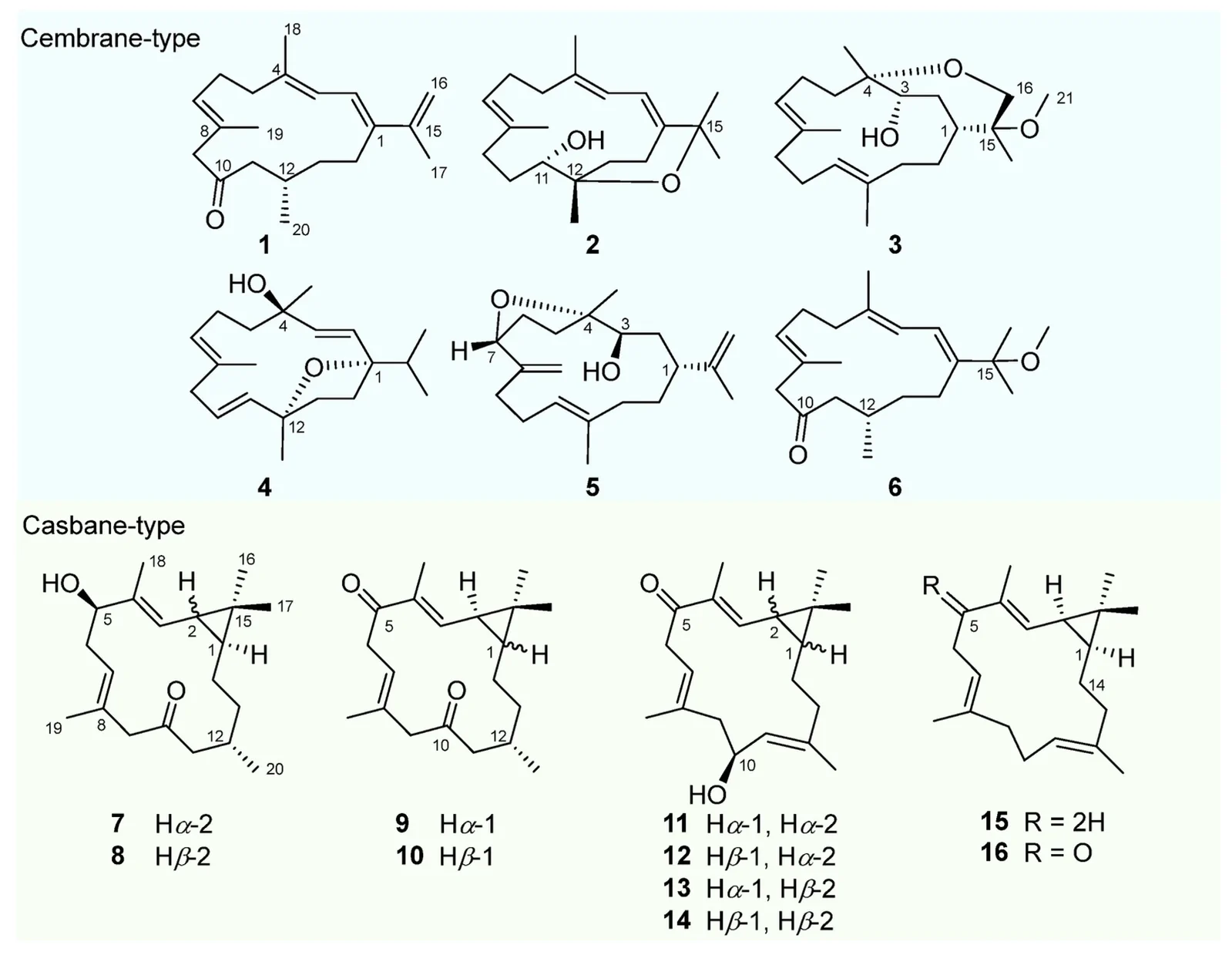
Chemical structures of compounds **1**–**16**.

Inspired by our previous work, a new collection of the title species off the Ximao Island, South China Sea, were studied, which led to the discovery of 16 diterpenes (Figure 1). Among them, five previously unreported cembrane-type diterpenes and one previously described casbane-type diterpene were characterized. All isolated compounds were evaluated for their cytotoxicity against several human tumor cell lines, namely, MV-4-11 and A-549, as well as the normal human embryonic kidney (HEK) 293T cell line, and for their anti-inflammatory effects against murine macrophage cell line RAW264.7. Herein, the isolation, structural characterization, and bioactivity evaluation of these compounds are reported.

## 2. Results

Samples of *S. nanolobata* were frozen immediately at –20 °C after collection and stored at that temperature until exhaustive extraction with dichloromethane and methanol (1:1, *v*/*v*), which was used as an alternative to conventional acetone extraction due to regulatory restrictions on acetone; this mixed solvent has comparable polarity, is effective for extracting secondary metabolites of varying polarities, and has been reported to afford higher recovery than other solvents [10]. The EtOAc-soluble portion of the organic extract was subjected to repeated column chromatography (CC) (silica gel, Sephadex LH-20, and reversed-phase HPLC), yielding five new cembrane-type diterpenes (**1**–**5**), one new casbane-type diterpene (**7**), and ten known related compounds (**6**, **8**–**16**). Those known compounds were rapidly characterized as 12α-methyl-1*E*,3*E*,7*E*-cembratrien-10-one (**6**) [11], nanolobatone B (**8**) [9], 10-*oxo*-11,12-dihydrodepressin (**9**) [12], 1-*epi*-10-*oxo*-11,12-dihydrodepressin (**10**) [12], 10-hydroxydepressin (**11**) [12], 1-*epi*-10-hydroxydepressin (**12**) [12], 2-*epi*-10-hydroxydepressin (**13**) [13], sinularcasbane A (**14**) [14], casbene (**15**) [15], and 5-*oxo*-casbene (**16**) [12], respectively, by comparison of their NMR data (Figures S70–S89) and optical rotation [*α*]_D_ values with those reported in the literature.

**Figure 2:**
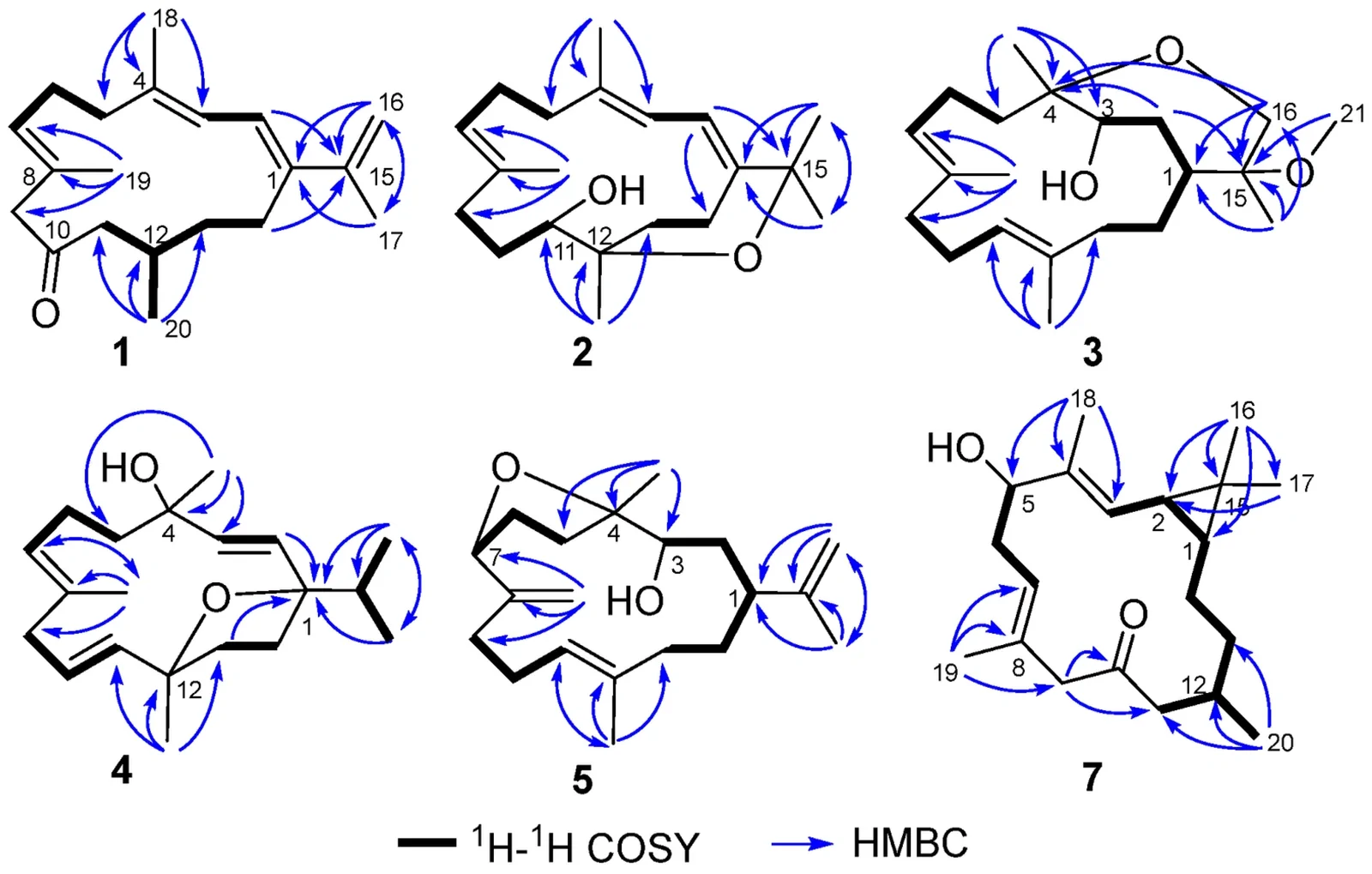
^1^H–^1^H COSY and selected key HMBC correlations of compounds **1**–**5** and **7**.

**Figure 3:**
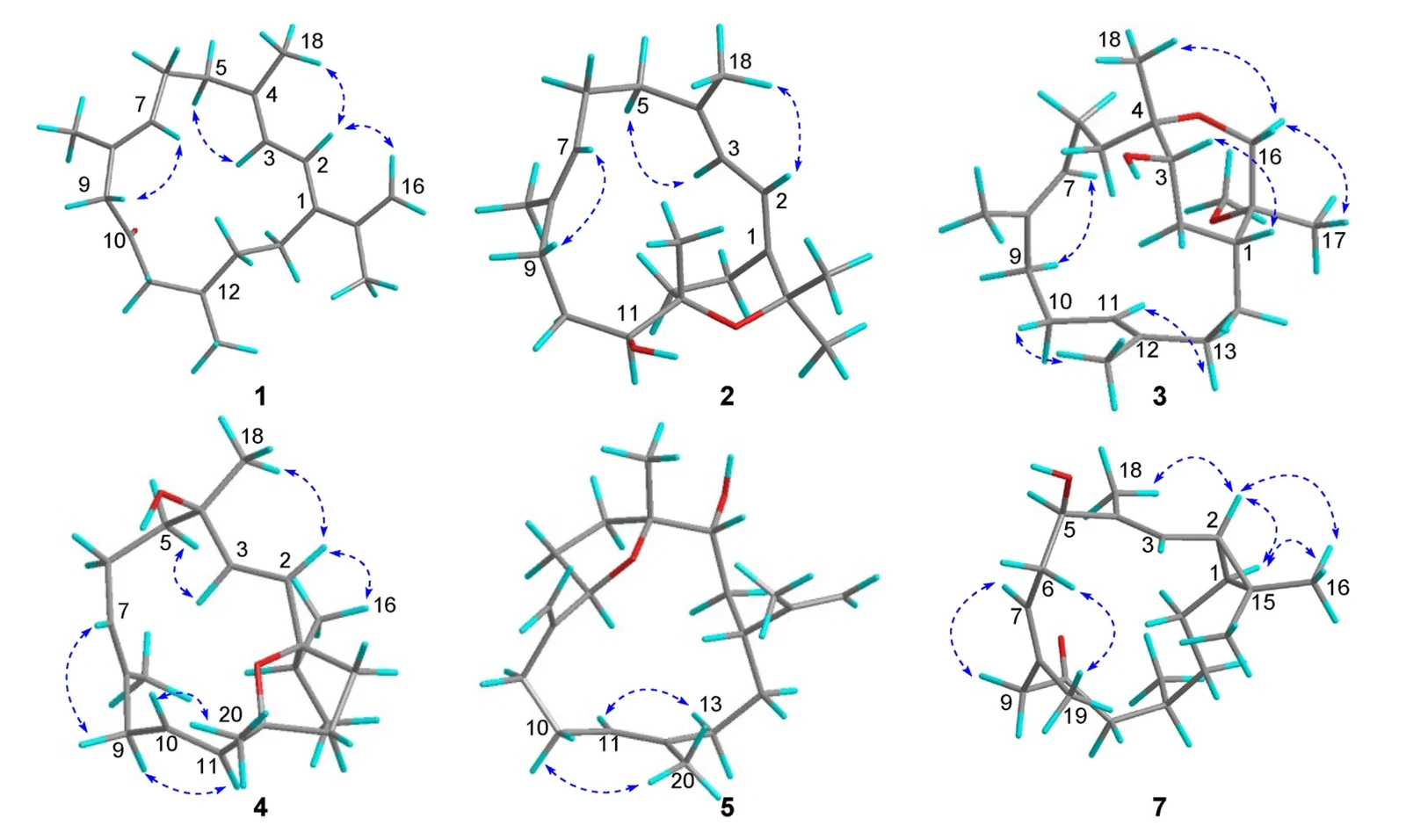
The NOESY correlations (blue dashed line) of compounds **1**–**5** and **7**.

Compound **1** was isolated as a colorless oil with the molecular formula of C_20_H_30_O, which was established by the protonated molecular ion peak observed at *m*/*z* 287.2364 [M + H]^+^ (calcd. for C_20_H_31_O^+^, 287.2369) in the HRESIMS spectrum, implying six degrees of unsaturation. The ^1^H NMR spectroscopic data of **1** (Table 1) revealed three vinyl methyls at *δ*_H_ 1.92 (3H, s), 1.78 (3H, s) and 1.60 (3H, s), one doublet methyl at *δ*_H_ 0.90 (3H, d, *J* = 6.7 Hz), and five olefinic protons at *δ*_H_ 6.31 (1H, d, *J* = 11.2 Hz), 6.02 (1H, d, *J* = 11.2 Hz), 5.25 (1H, t, *J* = 6.8 Hz), 5.03 (1H, s) and 4.95 (1H, s). The ^13^C NMR spectroscopic data of **1** (Table 2) showed 20 carbon signals, identified with the aid of the HSQC spectrum, including four methyls (*δ*_C_ 16.6, 16.9, 21.1, 21.4), eight olefinic carbons (*δ*_C_ 112.4, 122.7, 123.0, 129.2, 130.3, 138.4, 139.2, 143.5), one carbonyl carbon (*δ*_C_ 209.8), and seven methylene carbons (*δ*_C_ 24.0, 25.6, 27.7, 35.9, 39.9, 47.1, 54.3). The above-mentioned four double bonds and one carbonyl accounted for five of the six degrees of unsaturation, indicating a monocyclic carbon framework for **1**. The NMR data of **1** were almost identical to those of the previously reported compound, 12*α*-methyl-1*E*,3*E*,7*E*-cembratrien-10-one (**6**) [11], except that the isopropyl with a methoxy group at C-15 in **6** was replaced by an isopropenyl group in **1**. This replacement was corroborated by the HMBC correlations from H_2_-16 to C-15 and C-17. The planar structure of **1** was finally determined as depicted in Figure 2 through analysis of the ^1^H–^1^H COSY and HMBC spectra. The geometries of double bonds Δ^1^, Δ^3^ and Δ^7^ were assigned as “*E*” based on the shielded carbon resonances of two vinyl methyls at *δ*_C_ 16.9 (C-18), 16.6 (C-19), as well as the observed NOESY correlations of H-2/Me-18, H-3/H_2_-5, H-7/H_2_-9 (Figure 3). Structurally, compound **1** and compound **6** are homologs sharing the same single chiral center at C-12, and there are only two possible absolute configurations, which are closely correlated with their optical rotation values. Therefore, the absolute configuration of compound **1** {${\left[\alpha \right]}_{D}^{20}$ -2.2 (*c* 0.18, CHCl_3_)} was determined to be the same, 12*R,* as that of compound **6** {${\left[\alpha \right]}_{D}^{20}$ -13.5 (*c* 0.25, CHCl_3_)} based on a comparison of optical rotation values recorded under the same conditions. Therefore, the structure of **1** was tentatively established as shown in Figure 1 and named 12α-methyl-1*E*,3*E*,7*E,*15-cembratetraen-10-one.

Compound **2** was isolated as a colorless oil, and its molecular formula C_20_H_32_O_2_ was established by the protonated molecular ion peak observed at *m*/*z* 305.2477 [M + H]^+^ (calcd. for C_20_H_33_O_2_^+^, 305.2475) in the HRESIMS spectrum, which was 18 mass units more than that of **1**, corresponding to five degrees of unsaturation. A comparison of the 1D NMR data of **2** with those of **1** suggested that they were structural analogs, both being cembrane-type diterpenes. The ^1^H NMR spectrum displayed three olefinic protons at *δ*_H_ 6.19 (1H, d, *J* = 10.0 Hz), 6.00 (1H, d, *J* = 10.0 Hz) and 5.06 (1H, t, *J* = 7.8 Hz), which were attributed to three trisubstituted double bonds. These moieties accounted for three of the five degrees of unsaturation, suggesting that **2** possessed a bicyclic system in its molecular structure. Moreover, the ^13^C NMR signals at *δ*_C_ 70.1 (CH), 74.7 (qC) and 76.0 (qC) confirmed the presence of a hydroxyl group and an additional oxygen-containing ring in **2**, accounting for the remaining two oxygen atoms. The location of the hydroxyl group at C-11 and the formation of the oxygen ring via the linkage of C-12 and C-15, respectively, are evidenced by the HMBC correlations from Me-16/17 to C-1 (*δ*_C_ 138.4), C-15 (*δ*_C_ 74.7) and C-17 (*δ*_C_ 29.9)/C-16 (*δ*_C_ 28.9) and from Me-20 to C-11 (*δ*_C_ 70.1), C-12 (*δ*_C_ 76.0), C-13 (*δ*_C_ 30.2). Thus, the planar structure of compound **2** was established on the basis of the collective spectroscopic evidence (Figure 2).

**Figure 4:**
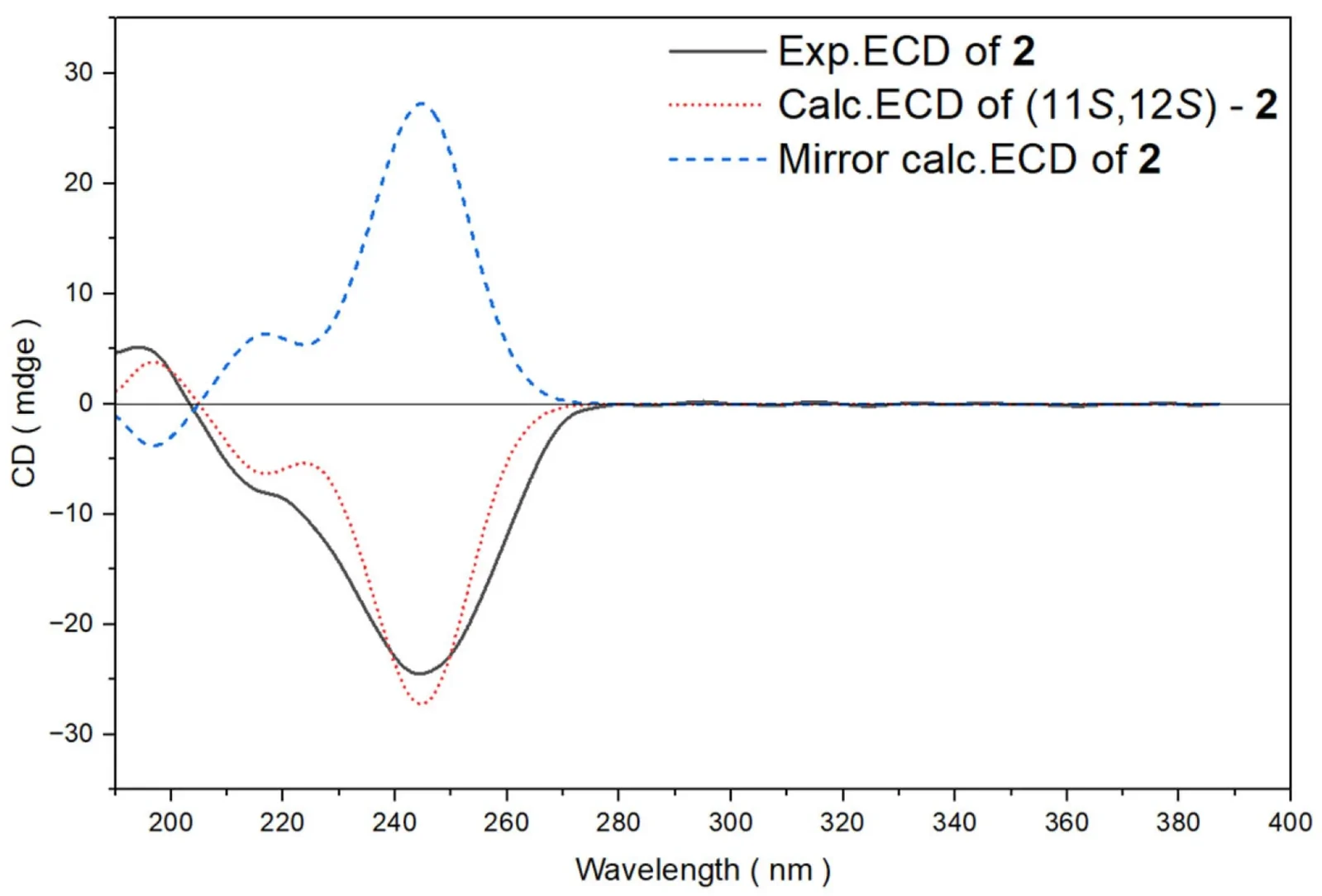
Experimental ECD spectrum of compound **2** (black line) and calculated ECD curves of (11*S*, 12*S*)-**2** (red dashed line) and its enantiomer (blue dashed line).

All the geometries of the double bonds Δ^1^, Δ^3^ and Δ^7^ were assigned to be “*E*” based on the NOESY correlations of H-2/Me-18, H-3/H_2_-5, H-7/H_2_-9 (Figure 3). The relative configuration of C-11 and C-12 was determined to be 11S*,12S* by the QM-NMR calculation and DP4+ probability analysis, where the experimentally observed NMR data of **2** gave the best match of 100% to those of the (11S*,12S*)-isomer (Figure S58). Subsequently, the absolute configuration of **2** was further elucidated to be 11*S*,12*S* via TDDFT-ECD calculation, showing that the Boltzmann-averaged ECD curve of (11*S*,12*S*)-**2** highly matched the experimental ECD spectrum of **2**, whereas the enantiomer exhibited a completely opposite curve (Figure 4). Accordingly, the structure of **2** was characterized as shown in Figure 1, and the compound was designated with the trivial name nanolobatol A.

Compound **3** was also isolated as a colorless oil, with the molecular formula C_21_H_36_O_3_ established by HRESIMS from the molecular ion peak observed at *m*/*z* 337.2737 [M + H]^+^ (calcd. for C_21_H_37_O_3_^+^, 337.2737), suggesting that **3** possessed four degrees of unsaturation. The ^1^H, ^13^C NMR data and HSQC spectrum of **3** displayed 21 distinct carbon signals, including five singlet methyl groups (*δ*_H_/*δ*_C_ 0.85/19.4, 1.07/19.2, 1.51/15.1, 1.60/15.0, 3.26/50.8), eight methylene groups (*δ*_C_ 22.4, 24.9, 25.3, 32.0, 36.8, 38.4, 39.5, 70.0), four methine groups (*δ*_C_ 30.9, 73.3, 127.7, 127.8), and four quaternary carbons (*δ*_C_ 79.0, 80.3, 133.4, 134.0). Based on the above data, the presence of two trisubstituted double bonds accounted for two of the four degrees of unsaturation, indicating that compound **3** was a bicyclic molecule. Furthermore, comprehensive analysis of the ^1^H–^1^H COSY spectrum revealed three distinct spin systems, as evidenced by the observed cross-peaks for H-3/H_2_-2/H-1/H_2_-14/H_2_-13, H_2_-5/H_2_-6/H-7, H_2_-9/H_2_-10/H-11. Additionally, the key HMBC correlations from Me-18 to C-3, C-4, and C-5 revealed the substitution of the hydroxyl at C-3. The HMBC cross-peaks of H_2_-16/C-4 were diagnostic for the ring linkage of C-4 and C-16. The methoxyl group at C-15 was indicated by the characteristic HMBC correlations from Me-21 to C-15 (Figure 2). Consequently, the planar structure of compound **3** was collectively established.

**Figure 5:**
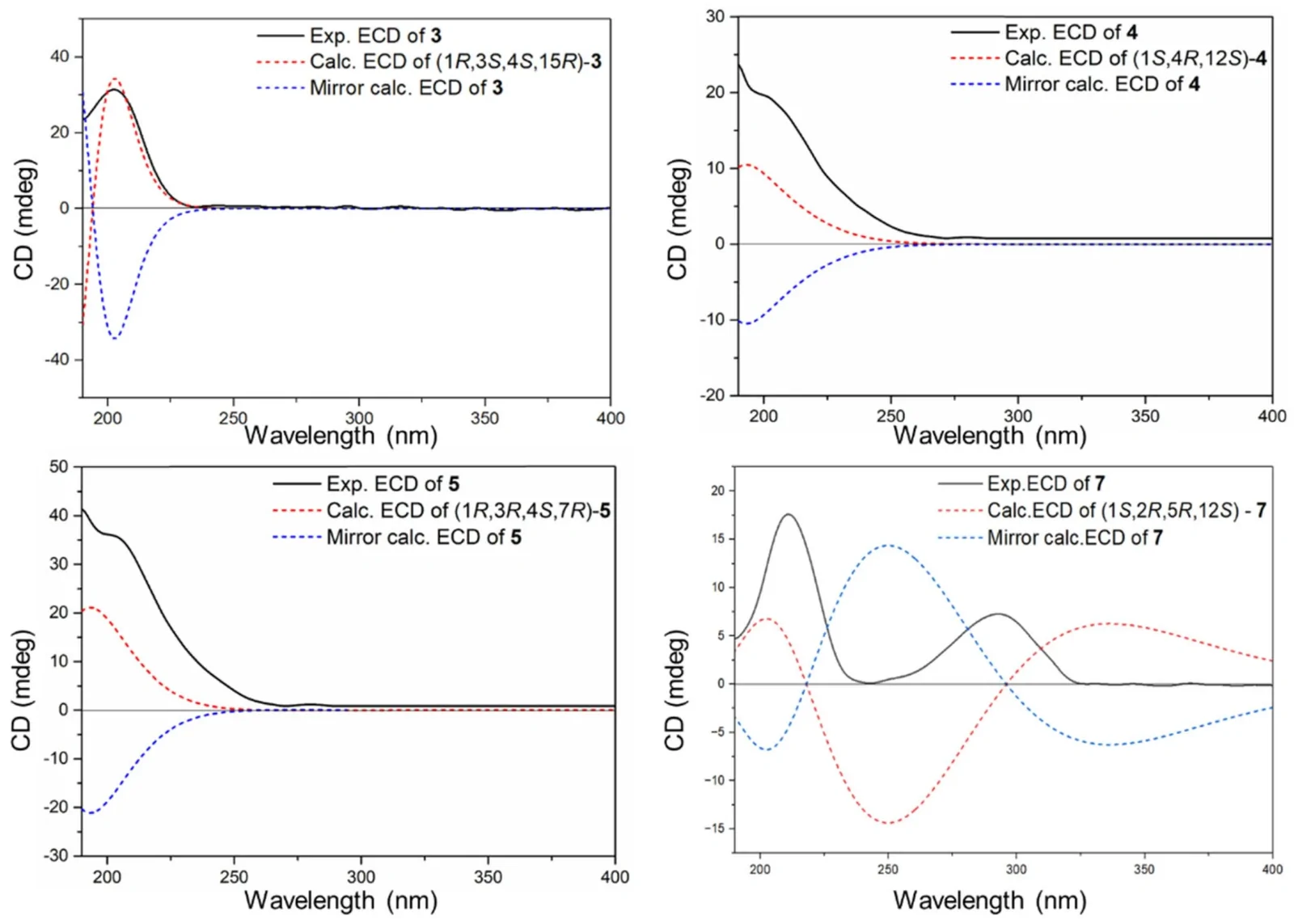
Experimental ECD spectra and calculated ECD curves of compounds **3**–**5** and **7**.

The geometries of the double bonds Δ^7^ and Δ^11^ were both assigned as “*E*” based on the shielded carbon resonances of the two vinyl methyl groups at *δ*_C_ 15.1 (C-19) and 15.0 (C-20). This assignment was further supported by the NOESY correlations (Figure 3) of H-7/H-9 and H-11/H-13. The relative configurations of C-1/C-3 and C-4/C-15 in compound **3** were determined to be 1R*,3S* and 4S*,15R*, respectively, based on the diagnostic NOESY correlations of H-1 (*δ*_H_ 1.96)/H-3 (*δ*_H_ 4.26) and Me-17 (*δ*_H_ 0.85)/H-16 (*δ*_H_ 3.64)/Me-18 (*δ*_H_ 1.07). However, the relationships of the relative configurations of two chiral domains could not be unambiguously determined by NOESY analysis. Assisted by the QM-NMR calculation and DP4+ probability analysis, the full relative configuration of **3** was established as 1R*,3S*,4S*,15R*, because the experimental NMR data of **3** showed the best match (100%) with those of the (1R*,3S*,4S*,15R*)-isomer (Figure S60). As shown in Figure 5, the experimental ECD spectrum of **3** was in good agreement with the calculated curve for (1*R*,3*S*,4*S*,15*R*)-**3**, while being completely opposite to that of its enantiomer. Consequently, the absolute configuration of **3** was determined to be 1*R*,3*S*,4*S*,15*R* by the TDDFT-ECD calculation. Herein, the structure of **3** (trivial name nanolobatol B) was proposed as depicted.

Compound **4** was obtained as a colorless oil. The molecular formula of **4** was established as C_20_H_32_O_2_ based on the protonated molecular ion peak observed at *m*/*z* 305.2471 [M + H]^+^ (calcd. for C_20_H_33_O_2_^+^, 305.2475) in its HRESIMS spectrum, implying five degrees of unsaturation. The ^1^H NMR data displayed signals corresponding to one vinyl methyl at *δ*_H_ 1.61 (3H, s), two tertiary methyls at *δ*_H_ 1.26 (3H, s) and 1.33 (3H, s), two methyls appearing as doublets at *δ*_H_ 0.88 (6H, d, *J* = 6.8 Hz), and five olefinic protons at *δ*_H_ 5.24 (1H, t, *J* = 7.5 Hz), 5.51 (1H, d, *J* = 16 Hz), 5.52 (1H, dd, *J* = 16, 6 Hz), 5.64 (1H, d, *J* = 15.8 Hz) and 5.71 (1H, d, *J* = 15.8 Hz), which were attributed to two disubstituted and one trisubstituted double bonds. Based on the above, three double bonds accounted for three of the five degrees of unsaturation, indicating that a bicyclic ring system belonged to the molecular structure of **4**. The HMBC correlations from Me-18 to C-2, C-3 and C-4 and from H-2 to C-1 corroborated the location of the hydroxyl group at C-4 and the settlement of double bond Δ^2^. The characteristic HMBC cross-peaks of Me-20/C-12, H_2_-13/C-1, along with the ^1^H–^1^H COSY cross-peaks for H_2_-13/H_2_-14, revealed the linkage of C-1 and C-12, forming the tetrahydrofuran ring. Finally, the planar structure of **4** was unequivocally established as shown in Figure 2.

The large coupling constants (*J*_2,3_ = 15.8 Hz, *J*_10,11_ = 16 Hz) established the “*E*” geometries of double bonds Δ^2^ and Δ^10^. Additionally, the shielded carbon resonance of the vinyl methyl at *δ*_C_ 17.6 (C-19) assigned the “*E*” geometry to the double bond Δ^7^. These assignments were further confirmed by analysis of the NOESY correlations (Figure 3). The results of the QM-NMR/DP4+ calculations (Figure S62) confirmed the relative configuration of compound **4** to be 1S*,4R*,12S*. Moreover, the absolute configuration of **4** was also determined by comparing its experimental ECD spectrum with the TDDFT-ECD-calculated curves of its two enantiomers, showing excellent agreement with the one of the (1*S*,4*R*,12*S*)-**4** isomers (Figure 5). Thus, the structure of **4** (trivial name nanolobatol C) was assigned as depicted.

Compound **5** was isolated as a colorless oil, possessing the same molecular formula of C_20_H_32_O_2_ as **4** by the HRESIMS ion peak at *m*/*z* 305.2472 [M + H]^+^ (calcd. for C_20_H_33_O_2_^+^, 305.2475). The ^1^H NMR data revealed the presence of two vinyl methyls at *δ*_H_ 1.60 (3H, s) and 1.70 (3H, s), one tertiary methyl at *δ*_H_ 1.19 (3H, s), and five olefinic protons at *δ*_H_ 4.77 (2H, s), 4.86 (1H, s), 4.91 (1H, s) and 5.30 (1H, t, *J* = 7.6 Hz), assigned to one trisubstituted and two terminal double bonds. These structural fragments accounted for three of the five degrees of unsaturation, indicating that a bicyclic ring system was incorporated in the molecular structure of **5**. Subsequently, comprehensive analysis of the key ^1^H–^1^H COSY cross-peaks and long-range HMBC correlations collectively established the connectivity of the spin systems and the assembly of all structural fragments. In detail, the ^1^H–^1^H COSY cross-peaks of H-3/H_2_-2/H-1/H_2_-14/H_2_-13, H_2_-5/H_2_-6/H-7, and H_2_-9/H_2_-10/H-11 revealed three distinct spin systems. The HMBC correlations from Me-17 to C-1 (*δ*_C_ 41.4), C-15 (*δ*_C_ 150.2), and C-16 (*δ*_C_ 110.9); from Me-18 to C-3 (*δ*_C_ 73.6), C-4 (*δ*_C_ 84.9), and C-5 (*δ*_C_ 37.1); from H_2_-19 to C-7 (*δ*_C_ 80.3), C-8 (*δ*_C_ 151.9), and C-9 (*δ*_C_ 34.3); and from Me-20 to C-11 (*δ*_C_ 126.3), C-12 (*δ*_C_ 134.5), and C-13 (*δ*_C_ 36.5) revealed the linkage between C-4 and C-7, forming the tetrahydrofuran ring. In addition, the hydroxy group was established at C-3, and the two terminal double bonds were assigned to C-8 and C-15, respectively, thereby unambiguously determining the planar structure of **5** (Figure 2).

The “*E*” geometry of the Δ^11,12^ double bond was assigned based on the shielded carbon resonances of the vinyl methyl at *δ*_C_ 17.2 (C-20). The four stereogenic centers (C-1, C-3, C-4, and C-7) in compound **5** were all located on a flexible macrocyclic ring and remote from each other, which prevented definitive configurational assignment by NOESY analysis and thus required QM-NMR/DP4+ calculations. Comparison of the calculated NMR data with the experimental values showed excellent agreement (99.99%) for the 1R*,3R*,4S*,7R* configuration (Figure S64). Following the same TDDFT-ECD calculation protocol as applied to the other compounds in this study, the absolute configuration of **5** was finally established as 1*R*,3*R*,4*S*,7*R* (Figure 5). Accordingly, the structure of **5** (trivial name nanolobatol D) was characterized as depicted.

Compound **7** was isolated as a colorless oil. Its molecular formula was determined to be C_20_H_32_O_2_, based on the HRESIMS ion peak observed at *m*/*z* 305.2475 [M + H]^+^ (calcd. for C_20_H_33_O_2_^+^, 305.2475). The ^1^H and ^13^C NMR data of **7** were highly similar to those of the co-occurring known casbane-type diterpenoid, nanolobatone B (**8**) [9], with the main differences observed at C-1, C-2 and their adjacent carbons, suggesting that they were structural analogs and epimers at either the C-1 or C-2 position. The large Δ*δ*_C_ value (more than 10 ppm) of the gem-dimethyls C-16 (*δ*_C_ 29.1) and C-17 (*δ*_C_ 15.9) indicated a 1,2-*cis* configuration for **7**, which was further supported by the obvious NOESY correlations of H-1 (*δ*_H_ 0.61)/Me-16 (*δ*_H_ 1.06) and H-2 (*δ*_H_ 1.24)/H-1 (Figure 3). Careful comparison of the NMR data of **7** with those of the reported synthetic product **S5** [16], particularly the chemical shifts of H-1/C-1 and H-2/C-2, indicated that **7** shared the same 1S*,2R* relative configuration as **S5**. Furthermore, the relative configuration of 5R*,12S* in **7** was confirmed to be identical to that of **8** based on the similarity of their NMR data. Therefore, the relative configuration of **7** was assigned as 1S*,2R*,5R*,12S*. Finally, the absolute configuration of **7** was determined to be 1*S*,2*R*,5*R*,12*S* by TDDFT-ECD calculations, as the experimental ECD spectrum matched well with the calculated curve for (1*S*,2*R*,5*R*,12*S*)-**7** (Figure 5). Consequently, compound **7** was identified as the C-2 epimer of compound **8**, named 2-*epi*-nanolobatone B.

**Figure 6:**
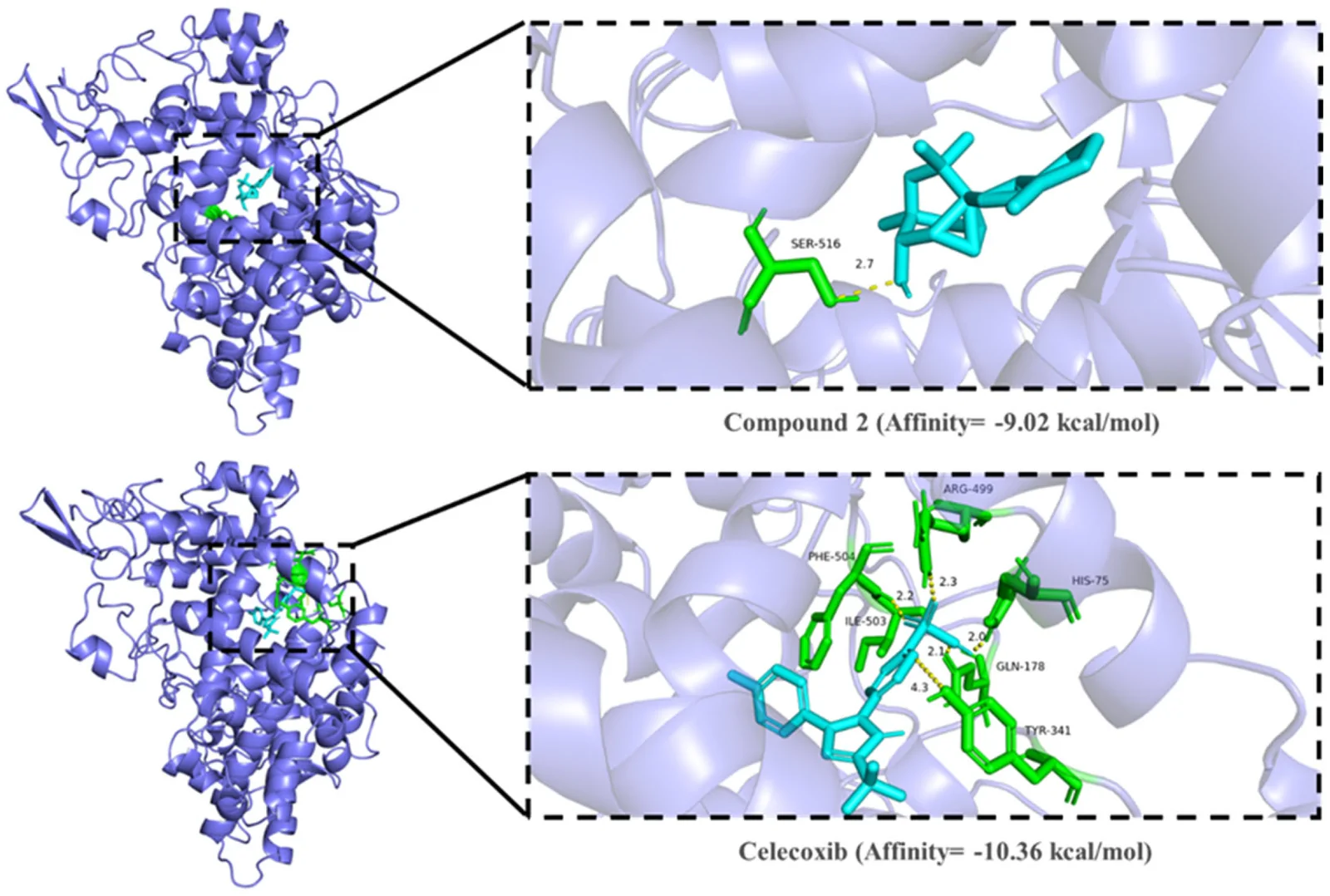
Molecular docking binding affinity and interactions of compound **2** with COX-2.

In the in vitro bioassay, all isolated compounds were evaluated for their cytotoxic activities against MV-4-11, A-549, and 293T cell lines, as well as for their anti-inflammatory effects in RAW264.7 macrophages. Unfortunately, none of the compounds exhibited obvious bioactivity (IC_50_ > 50 µM). Comparatively, previous studies have reported that certain cembrane- and casbane-type diterpenes from *Sinularia* species exhibit moderate cytotoxic and/or anti-inflammatory effects, with IC_50_ values typically ranging from 10 to 50 µM. A structure–activity relationship (SAR) discussion was conducted by comparing the structures of the isolated cembrane-type diterpenoids with previously reported bioactive analogs from *Sinularia* species. It is noteworthy that the *α*-methylene-*γ*-lactone or *α*,*β*-unsaturated-*γ*-lactone moieties have been demonstrated to make major contributions to cytotoxic and anti-inflammatory properties [17]. However, none of the compounds isolated in the present study contain such lactone structural units, which may partially account for their lack of significant bioactivity. To elucidate the absence of anti-inflammatory activity observed in cellular screening assays, molecular docking simulations of representative compound **2** against COX-2 (PDB: 3LN1) were carried out, employing celecoxib as the reference ligand. The reference inhibitor celecoxib exhibited a binding affinity of −10.36 kcal/mol and formed multiple hydrogen-bond interactions with key residues ARG-499, GLN-178, and HIS-75 within the active pocket, allowing stable occupancy of the protein active site. In comparison, compound **2** possessed a lower binding affinity (−9.02 kcal/mol) and only established a single hydrogen bond with SER-516 (Figure 6). This may give rise to unstable ligand–protein binding, which consequently contributes to its failure to exert anti-inflammatory activity.

For the casbane-type diterpenoids, a comparison was made with our previously reported anti-inflammatory analogs [18]. Compared with the bioactive compounds reported earlier, the compounds isolated in the present study exhibit lower oxidation states at C-8 and C-10, reduced unsaturation, and simpler oxidation patterns. These structural differences may account for the weaker anti-inflammatory activity observed for the casbane-type compounds in this study.

Despite the observed weak or negligible activities of the compounds in this study, evaluating these activities remains a routine and essential step for the full characterization of new natural products, whose potency may vary depending on structural substitution patterns. Notably, the lack of activity observed in the present study does not preclude the possibility of other biological effects, such as antimicrobial or neuroprotective activities, and additional bioassays to explore these possibilities are currently in progress.

## 3. Materials and Methods

### 3.1. General Experimental Procedures

Optical rotations were measured on a PerkinElmer 241MC polarimeter (PerkinElmer, Fremont, CA, USA). NMR spectra were measured in CDCl_3_ with a Bruker Advance 600 MHz spectrometer (Bruker Biospin AG, Fällanden, Germany). Chemical shifts are reported in parts per million (*δ*) in CDCl_3_ (δ_H_ reported referred to CHCl_3_ at 7.26 ppm; δ_C_ reported referred to CDCl_3_ at 77.2 ppm), and coupling constants (*J*) are expressed in Hz. HRESIMS spectra were recorded on an Agilent 1290-6545 UHPLC-QTOF mass spectrometer (Agilent Technologies, Santa Clara, CA, USA). Commercial silica gel (Qingdao Haiyang Chemical Group Co., Ltd., Qingdao, China, 200–300 and 300–400 mesh) and Sephadex LH-20 gel (Amersham Biosciences, Uppsala, Sweden) were used for column chromatography, and precoated silica gel plates (Yan Tai Zi Fu Chemical Group Co., Yantai, China, G60 F-254) were used for analytical TLC. Reversed-phase (RP) HPLC was performed on an Agilent 1260 series liquid chromatography system equipped with a DAD G1315D detector at 210 and 254 nm. A semi-preparative ODS-HG-5 column [5 µm, 250 × 9.4 mm] was employed for the purifications. All solvents used for CC and HPLC were of analytical grade (Shanghai Chemical Reagents Co., Ltd., Shanghai, China) and chromatographic grade (Dikma Technologies Inc., Beijing, China), respectively.

### 3.2. Animal Materials

Specimens of *S. nanolobata* were collected by scuba diving along the coast off Ximao Island (18°13.8′ N, 109°22.1′ E), Hainan Province, China, in May 2019, at a depth of 20 m. A voucher specimen (19-XD-12) has been deposited and is available for inspection at the School of Medicine, Shanghai University.

### 3.3. Extraction and Isolation

The frozen animals (260.5 g, dry weight after extraction) were cut into pieces and extracted exhaustively with CH_2_Cl_2_/MeOH (1:1, *v*/*v*) at room temperature (2.0 L × 4). The organic extract was evaporated to give a brown residue, which was then partitioned between EtOAc and H_2_O. The EtOAc solution was evaporated to give a dark brown residue (15.7 g). The obtained residue was subjected to gradient silica gel (200–300 mesh) column chromatography (CC) [EtOAc/petroleum ether (PE) 0 → 100%] to yield five fractions (Fr. A–E).

Fraction A (127.4 mg) was directly purified by semi-preparative RP-HPLC (CH_3_CN/H_2_O, 98:2, 3.0 mL/min) to afford compound **15** (6.0 mg, t_R_ = 27.5 min).

Fraction B (5.38 g) was separated by Sephadex LH-20 column chromatography eluted with PE/CH_2_Cl_2_/MeOH (2:1:1) to give four sub-fractions (B1–B4). Sub-fraction B4 (66.5 mg) was further purified by silica gel column chromatography (300–400 mesh) eluted with PE/EtOAc (40:1 and 15:1), followed by semi-preparative RP-HPLC (CH_3_CN/H_2_O, 87:13, 3.0 mL/min) to yield compound **16** (4.2 mg, t_R_ = 21.3 min).

Fraction C (1.26 g) was first separated by Sephadex LH-20 column chromatography eluted with PE/CH_2_Cl_2_/MeOH (2:1:1) to give three sub-fractions (C1–C3). Sub-fraction C2 was separated by silica gel column chromatography (300–400 mesh) eluted with PE/EtOAc (30:1, 15:1, 5:1, and 1:1) to afford five secondary fractions (C2a–C2e). Fraction C2a was further separated by RP-HPLC (CH_3_CN/H2O, 80:20, 3.0 mL/min) to obtain compounds **4** (2.1 mg, t_R_ = 16.6 min) and **5** (1.6 mg, t_R_ = 19.2 min). Sub-fraction C2d was purified by semi-preparative RP-HPLC (CH_3_CN/H_2_O, 80:20, 3.0 mL/min) to yield compound **3** (2.6 mg, t_R_ = 18.7 min). Sub-fraction C2e was purified by semi-preparative RP-HPLC (CH_3_CN/H_2_O, 80:20, 3.0 mL/min) to give compounds **9** (12.3 mg, t_R_ = 10.8 min) and **10** (9.1 mg, t_R_ = 11.3 min).

Fraction D (1.36 g) was first separated by Sephadex LH-20 column chromatography eluted with PE/CH_2_Cl_2_/MeOH (2:1:1) to obtain four sub-fractions (D1–D4). Sub-fraction D2 (528.4 mg) was subjected to silica gel column chromatography (300–400 mesh) eluted with PE/EtOAc (30:1, 20:1, 10:1, 5:1, and 1:1) to afford compound **14** (2.6 mg) and five secondary fractions (D2a–D2e). Sub-fraction D2e was purified by semi-preparative RP-HPLC (MeOH/H_2_O, 83:17, 3.0 mL/min) to yield compound **2** (1.8 mg, t_R_ = 16.4 min) and a tertiary fraction (10.2 mg, t_R_ = 24.8 min). Sub-fraction D3 (554.2 mg) was separated by silica gel column chromatography (300–400 mesh) eluted with PE/EtOAc (30:1, 20:1, 10:1, 5:1, 1:1) to yield compound **6** (3.9 mg) and three secondary fractions (D3a–D3c). Sub-fraction D3c was purified by semi-preparative RP-HPLC (CH_3_CN/H_2_O, 67:33, 3.0 mL/min) to afford compounds **7** (3.2 mg, t_R_ = 12.5 min) and **8** (2.8 mg, t_R_ = 13.3 min).

Fraction E (1.06 g) was initially separated by Sephadex LH-20 column chromatography eluted with PE/CH_2_Cl_2_/MeOH (2:1:1) to give three sub-fractions (E1–E3). Sub-fraction E3 was separated by silica gel column chromatography (300–400 mesh) eluted with PE/EtOAc (30:1, 20:1, 10:1, 5:1, 1:1) to afford compound **1** (2.4 mg) and three secondary fractions (E3a–E3c). Sub-fraction E3c was purified by semi-preparative RP-HPLC (CH_3_CN/H_2_O, 55:45, 3.0 mL/min) to afford compounds **11** (11.4 mg, t_R_ = 17.3 min), **12** (2.1 mg, t_R_ = 18.7 min), and **13** (4.1 mg, t_R_ = 19.3 min).

#### 3.3.1. 12α-Methyl-1*E*,3*E*,7*E*,15-cembratetraen-10-one (**1**)

Colorless oil, ${\left[\alpha \right]}_{D}^{20}$ -2.2 (*c* 0.18, CHCl_3_); for ^1^H and ^13^C NMR spectroscopic data, see Table 1 and Table 2; HRESIMS *m*/*z* 287.2364 [M + H]^+^ (calcd. for C_20_H_31_O^+^, 287.2369).

#### 3.3.2. Nanolobatol A (**2**)

Colorless oil, ${\left[\alpha \right]}_{D}^{20}$ -116.4 (*c* 0.14, CHCl_3_); for ^1^H and ^13^C NMR spectroscopic data, see Table 1 and Table 2; HRESIMS *m*/*z* 305.2477 [M + H]^+^ (calcd. for C_20_H_33_O_2_^+^, 305.2475).

#### 3.3.3. Nanolobatol B (**3**)

Colorless oil, ${\left[\alpha \right]}_{D}^{20}$ +10.5 (*c* 0.43, CHCl_3_); for ^1^H and ^13^C NMR spectroscopic data, see Table 1 and Table 2; HRESIMS *m*/*z* 337.2737 [M + H]^+^ (calcd. for C_21_H_37_O_3_^+^, 337.2737).

#### 3.3.4. Nanolobatol C (**4**)

Colorless oil, ${\left[\alpha \right]}_{D}^{20}$ +20.5 (*c* 0.10, CHCl_3_); for ^1^H and ^13^C NMR spectroscopic data, see Table 1 and Table 2; HRESIMS *m*/*z* 305.2471 [M + H]^+^ (calcd. for C_20_H_33_O_2_^+^, 305.2475).

#### 3.3.5. Nanolobatol D (**5**)

Colorless oil, ${\left[\alpha \right]}_{D}^{20}$ +6.5 (*c* 0.10, CHCl_3_); for ^1^H and ^13^C NMR spectroscopic data, see Table 1 and Table 2; HRESIMS *m*/*z* 305.2472 [M + H]^+^ (calcd. for C_20_H_33_O_2_^+^, 305.2475).

#### 3.3.6. 2-epi-Nanolobatone B (**7**)

Colorless oil, ${\left[\alpha \right]}_{D}^{20}$ +180.0 (*c* 0.16, CHCl_3_); for ^1^H and ^13^C NMR spectroscopic data, see Table 1 and Table 2; HRESIMS *m*/*z* 305.2475 [M + H]^+^ (calcd. for C_20_H_33_O_2_^+^, 305.2475).

### 3.4. Calculation Section

Conformational searches were performed using the torsional sampling (MCMM) approach and an OPLS_2005 force field within an energy window of 21 kJ/mol. Conformers with Boltzmann populations above 1% were re-optimized at the B3LYP/6-311G(d,p) level using the IEFPCM solvent model for chloroform. Frequency analysis was also carried out to confirm that the re-optimized geometries were at the energy minima. Subsequently, NMR calculations were performed at the PCM/mPW1PW91/6-31G(d) level, as recommended for DP4+ [19]. NMR shielding constants were calculated using the GIAO method. Finally, shielding constants were averaged over the Boltzmann distribution obtained for each stereoisomer and correlated with the experimental data. ECD spectra were obtained by TDDFT calculations with the B3LYP/6-311G(d,p) level with the IEFPCM solvent model for CH_3_CN. Finally, the Boltzmann-averaged ECD spectra of the compounds were obtained using SpecDis 1.62.

### 3.5. Cytotoxicity Assays

The in vitro cytotoxic activities of compounds **1**–**16** were evaluated against MV-4-11, A-549, and 293T cell lines using the CCK-8 assay, with doxorubicin (DOX) serving as the positive control. The human non-small-cell lung carcinoma cell line (A-549) was obtained from the National Collection of Authenticated Cell Cultures of China, while the human myeloid monocytic leukemia (MV-4-11) and human embryonic kidney (HEK) 293T cell lines were purchased from Zhejiang Meisen Cell Technology Co., Ltd. (Jinhua, China). All compounds were first dissolved in DMSO to prepare stock solutions at a concentration of 40 mM, which were then serially diluted to obtain test solutions at various concentrations for the different bioactivity assays. The final DMSO concentration in all assays was maintained at ≤0.1%, and no precipitation was observed at any of the test concentrations. The growth-inhibitory effects of compounds on cancer cells from different tissue sources were tested at five concentration gradients. The maximum tested concentration was 50 µM, diluted fivefold, and the cells were treated with the five concentration gradients for 72 h. Compounds showing an inhibition rate greater than 60% at 50 µM were selected for further screening, and their IC_50_ values were calculated.

### 3.6. Anti-Inflammatory Assays

The murine macrophage cell line RAW264.7 was obtained from the American Type Culture Collection (ATCC, Manassas, VA, USA). The detailed experimental procedures were consistent with those described in our previous work [18]. The half maximal cytotoxic concentration (CC_50_) and IC_50_ values were estimated using the log (inhibitor) vs. normalized response nonlinear fit (GraphPad Prism 6.0). Dexamethasone was used as the positive control.

## 4. Conclusions

In this study, a total of 16 compounds were isolated and identified from the South China Sea soft coral *S. nanolobata*, which possessed cembrane- and casbane-type carbon skeletons. Among them, compounds **1**–**5** were five new cembrane-type diterpenes, and compound **7** was a new casbane-type diterpene. Interestingly, the new compounds **2**–**5** featured rare five- to seven-membered oxygen-containing heterocyclic rings as their structural highlights. In bioassays, all isolated compounds showed no significant cytotoxic or anti-inflammatory activities (IC_50_ > 50 µM), suggesting that these novel structures may not be primarily responsible for such effects.

Given the structural novelty and the lack of cytotoxicity or anti-inflammatory activity observed, future investigations should explore alternative biological evaluations. For instance, antimicrobial, antiviral, neuroprotective, or enzyme-inhibitory assays may reveal other potential bioactivities of these rare oxacyclic ring systems. Furthermore, chemical derivatization or semi-synthesis of these novel scaffolds might improve their bioactivity profiles by introducing pharmacophoric groups. Overall, the discovery of these structurally unique diterpenoids extends the chemical diversity of *S. nanolobata* and provides valuable leads for future marine natural product-based drug discovery efforts, particularly in the search for novel mechanisms of action beyond conventional cytotoxicity and anti-inflammatory pathways.

## Figures and Tables

**Table 1:** ^1^H (600 MHz) NMR data of compounds **1–5** and **7** in CDCl_3_^a^.

No.	*δ*_H_ Mult. (*J* in Hz)					
	1	2	3	4	5	7
1	/	/	1.96 m	/	2.78 t (6.8)	0.61 td (9.3, 2.8)
2	6.31 d (11.2)	6.20 d (10.2)	1.44 m	5.64 d (15.8)	1.74 m	1.24 t (9.3)
	/	/	1.30 d (2.6)	/	1.77 m	/
3	6.02 d (11.2)	6.00 d (10.2)	4.26 dd (11.5, 3.5)	5.71 d (15.8)	3.55 t (6.6)	5.04 d (9.3)
4	/	/	/	/	/	/
5	2.20 m	2.75 td (12.6, 3.9)	1.93 s	1.68 m	1.76 m	4.05 dd (9.5, 6.1)
	2.20 m	1.87 d (2.9)	1.57 m	1.78 m	2.01 m	/
6	2.23 m	2.30 td (9.1, 5.2)	2.68 m	2.14 m	2.03 m	2.36 s
	2.23 m	2.08 m	1.91 m	2.15 m	2.15 m	2.37 d (8.3)
7	5.25 t (6.8)	5.06 t (7.8)	5.23 d (10.2)	5.23 t (7.5)	4.20 dd (10.1, 4.2)	4.98 t (7.8)
8	/	/	/	/	/	/
9	3.02 br s	2.12 m	2.14 m	2.53 d (5.6)	2.17 m	2.89 d (13.1)
	2.93 d (2.9)	1.98 m	2.21 m	2.53 d (5.6)	2.37 m	3.00 d (13.1)
10	/	1.89 dd (3.4, 1.4)	1.70 dq (16.7, 7.6)	5.52 dd (16, 6)	2.05 m	/
	/	1.19 br s	0.96 dd (16.7, 3.5)	/	2.30 m	/
11	2.91 d (12.2, 4.6)	3.56 d (11.3)	4.96 d (7.6)	5.51 d (15.8)	5.30 t (7.6)	2.64 dd (16.8, 7.0)
	1.97 m	/	/	/	/	1.96 dd (16.8,6.1)
12	1.88 m	/	/	/	/	2.17 m
13	1.56 m	2.21 dd (14.2, 9.9)	2.13 m	1.71 m	1.88 m	1.32 m
	1.28 m	1.92 br s	2.13 m	2.08 dd (12.0, 7.6)	2.12 m	1.32 m
14	2.50 m	2.69 m	2.50 m	1.93 m	1.53 m	1.44 m
	2.29 m	2.28 m	2.00 m	1.99 dd (12.0, 8.1)	1.59 m	0.93 m
15	/	/	/	1.66 m	/	/
16	5.03 s	1.31 s	3.64 d (14.8)	0.88 d (6.8)	4.77 s	1.06 s
	4.95 s	/	3.62 d (14.8)	/	4.77 s	/
17	1.92 s	1.36 s	0.86 s	0.88 d (6.8)	1.70 s	0.97 s
	/	/	/	/	/	/
18	1.78 s	1.82 s	1.07 s	1.26 s	1.19 s	1.67 s
19	1.60 s	1.63 s	1.51 s	1.61 s	4.86 s	1.69 s
	/	/	/	/	4.91 s	/
20	0.90 d (6.7)	1.04 s	1.60 s	1.33 s	1.60 s	0.92 d (6.9)
-OMe	/	/	3.26 s	/	/	/

^a^ ^1^H NMR (600 MHz) chemical shifts (*δ*_H_) are reported in ppm referenced to CHCl_3_ (*δ*_H_ 7.26). HSQC and HMBC experiments aided signal assignments. Multiplicity abbreviations: “s”, singlet; “d”, doublet; “t”, triplet; “m”, multiplet; “q”, quadruplet; and “br”, broad.

**Table 2:** ^13^C (150 MHz) NMR data of compounds **1–5** and **7** in CDCl_3_^a^.

No.	*δ*_C,_ Type					
	1	2	3	4	5	7
1	143.5, C	138.4, C	30.9, CH	88.1, C	41.4, CH	30.5, C
2	122.7, CH	119.9, CH	32.0, CH_2_	130.9, CH	36.5, CH_2_	25.3, CH
3	123.0, CH	121.5, CH	73.3, CH	136.6, CH	73.6, CH	125.8, CH
4	138.4, C	139.6, C	79.0, C	73.3, C	84.9, C	136.7, C
5	39.9, CH_2_	31.4, CH_2_	38.4, CH_2_	43.3, CH_2_	37.1, CH_2_	79.2, CH_2_
6	25.6, CH_2_	25.0, CH_2_	22.4, CH_2_	23.1, CH_2_	31.5, CH_2_	33.0, CH_2_
7	129.2, CH	124.2, CH	127.8, CH	126.1, CH	80.3, CH	123.8, CH
8	130.3, C	135.8, C	133.4, C	133.9, C	151.9, C	131.6, C
9	54.3, CH_2_	34.9, CH_2_	39.5, CH_2_	39.5, CH_2_	34.4, CH_2_	53.3, CH_2_
10	209.8, C	27.3, CH_2_	24.9, CH_2_	124.2, CH	29.4, CH_2_	209.4, C
11	47.1, CH_2_	70.1, CH	127.7, CH	138.9, CH	126.3, CH	47.7, CH_2_
12	27.7, CH_2_	76.0, C	134.0, C	82.8, C	134.5, C	27.0, CH
13	35.9, CH_2_	30.2, CH_2_	36.8, CH_2_	35.7, CH_2_	36.5, CH_2_	36.1, CH_2_
14	24.0, CH_2_	20.1, CH_2_	25.3, CH_2_	34.2, CH_2_	29.5, CH_2_	19.3, CH_2_
15	139.2, C	74.7, C	80.3, C	38.3, CH	150.2, C	20.6, C
16	112.4, CH_2_	28.9, CH_3_	70.0, CH_2_	17.8, CH_3_	110.9, CH_2_	29.1, CH_3_
17	21.4, CH_3_	29.9, CH_3_	19.4, CH_3_	18.6, CH_3_	19.6, CH_3_	15.9, CH_3_
18	16.9, CH_3_	23.4, CH_3_	19.2, CH_3_	30.1, CH_3_	22.2, CH_3_	10.3, CH_3_
19	16.6, CH_3_	16.2, CH_3_	15.1, CH_3_	17.6, CH_3_	110.2, CH_2_	17.3, CH_3_
20	21.1, CH_3_	22.2, CH_3_	15.0, CH_3_	28.0, CH_3_	17.2, CH_3_	20.5, CH_3_
21			50.8, CH_3_			

^a^ ^13^C NMR (150 MHz) chemical shifts (*δ*_C_) are reported in ppm referenced to CDCl_3_ (*δ*_C_ 77.16).

## Data Availability

Data are contained within the article or Supplementary Materials.

## Supplementary Materials

The following supporting information can be downloaded at https://www.mdpi.com/article/10.3390/md24090317/s1.
