# Supplementary material for: Cembrane- and Casbane-Type Diterpenes from the South China Sea Soft Coral Sinularia nanolobata
Source: Mar Drugs. 2026 Sep 10;24(9):317. doi: 10.3390/md24090317 (PMC13608546; doi:10.3390/md24090317)
Supplement: Supplementary file 1 [file marinedrugs-24-00317-s001.zip › marinedrugs-4503780-supplementary.pdf]

Supporting Information for

Cembrane- and casbane-type diterpenes from the South China Sea soft coral *Sinularia nanolobata*

Yao Dong[1], Xiao Xiao[1], Li-Gong Yao[2], Lin-Fu Liang[3,*], Song-Wei Li[1,*], and Yue-Wei Guo[1,2,*]

[1] School of Medicine, Shanghai University, Shanghai 200444, China; 18788380886@163.com (Y.D.); xiao_xiao0121@163.com (X.X.)

[2] Shandong Laboratory of Yantai Drug Discovery, Bohai Rim Advanced Research Institute for Drug Discovery, Yantai 264117, China; yaoligong@simm.ac.cn

[3] School of Chemistry and Chemical Engineering, Central South University of Forestry and Technology, Changsha 410004, China

*Correspondence: lianglinfu@csuft.edu.cn (L.-F.L.); songweili@shu.edu.cn (S.-W.L.); ywguo@simm.ac.cn (Y.-W.G.)

Content

1. Original spectra of 1. 5

Figure S1. ^{1}H NMR spectrum (600 MHz) of **1** in $CDCl_3$ 5

Figure S2. Localized amplified ^{1}H NMR spectrum (600 MHz) of **1** in $CDCl_3$ 5

Figure S3. ^{13}C NMR spectrum (150 MHz) of **1** in $CDCl_3$ 6

Figure S4. HSQC spectrum (600 MHz) of **1** in $CDCl_3$ 6

Figure S5. Localized amplified HSQC spectrum (600 MHz) of **1** in $CDCl_3$ 7

Figure S6. HMBC spectrum (600 MHz) of **1** in $CDCl_3$ 7

Figure S7. ^{1}H-^{1}H COSY spectrum (600 MHz) of **1** in $CDCl_3$ 8

Figure S8. NOESY spectrum (600 MHz) of **1** in $CDCl_3$ 9

Figure S9. HR-ESIMS spectrum of **1** 10

Figure S10. ECD and UV spectra of **1** 10

2. Original spectra of 2. 11

Figure S11. ^{1}H NMR spectrum (600 MHz) of **2** in $CDCl_3$ 11

Figure S12. Localized amplified ^{1}H NMR spectrum (600 MHz) of **2** in $CDCl_3$ 11

Figure S13. ^{13}C NMR spectrum (150 MHz) of **2** in $CDCl_3$ 12

Figure S14. HSQC spectrum (600 MHz) of **2** in $CDCl_3$ 12

Figure S15. Localized amplified HSQC spectrum (600 MHz) of **2** in $CDCl_3$ 13

Figure S16. HMBC spectrum (600 MHz) of **2** in $CDCl_3$ 13

Figure S17. ^{1}H–^{1}H COSY spectrum (600 MHz) of **2** in $CDCl_3$ 14

Figure S18. NOESY spectrum (600 MHz) of **2** in $CDCl_3$ 15

Figure S19. HR-ESIMS spectrum of **2** 16

Figure S20. ECD and UV spectra of **2** 16

3. Original spectra of 3. 17

Figure S21. ^{1}H NMR spectrum (600 MHz) of **3** in $CDCl_3$ 17

Figure S22. Localized amplified ^{1}H NMR spectrum (600 MHz) of **3** in $CDCl_3$ 17

Figure S23. ^{13}C NMR spectrum (150 MHz) of **3** in $CDCl_3$ 18

Figure S24. HSQC spectrum (600 MHz) of **3** in $CDCl_3$ 18

Figure S25. Localized amplified HSQC spectrum (600 MHz) of **3** in $CDCl_3$ 19

Figure S26. HMBC spectrum (600 MHz) of **3** in $CDCl_3$ 19

Figure S27. ^{1}H–^{1}H COSY spectrum (600 MHz) of **3** in $CDCl_3$ 20

Figure S28. NOESY spectrum (600 MHz) of **3** in $CDCl_3$ 21

Figure S29. HR-ESIMS spectrum of **3** 22

Figure S30. ECD and UV spectra of **3** 22

4. Original spectra of 4. 23

Figure S31. ^{1}H NMR spectrum (600 MHz) of **4** in $CDCl_3$ 23

Figure S32. ^{13}C NMR spectrum (150 MHz) of **4** in $CDCl_3$ 23

Figure S33. HSQC spectrum (600 MHz) of **4** in $CDCl_3$ 24

Figure S34. HMBC spectrum (600 MHz) of **4** in $CDCl_3$ 24

Figure S35. ^{1}H–^{1}H COSY spectrum (600 MHz) of **4** in $CDCl_3$ 25

Figure S36. NOESY spectrum (600 MHz) of **4** in $CDCl_3$ 25

Figure S37. HR-ESIMS spectrum of **4** 26

Figure S38. ECD and UV spectra of **4** 26

5. Original spectra of 5. .... **27**
Figure S39. ^{1}H NMR spectrum (600 MHz) of **5** in $CDCl_3$ 27
Figure S40. ^{13}C NMR spectrum (150 MHz) of **5** in $CDCl_3$ 27
Figure S41. HSQC spectrum (600 MHz) of **5** in $CDCl_3$ 28
Figure S42. HMBC spectrum (600 MHz) of **5** in $CDCl_3$ 28
Figure S43. ^{1}H–^{1}H COSY spectrum (600 MHz) of **5** in $CDCl_3$ 29
Figure S44. NOESY spectrum (600 MHz) of **5** in $CDCl_3$ 29
Figure S45. HR-ESIMS spectrum of **5** 30
Figure S46. ECD and UV spectra of **5** 30
6. Original spectra of 7. .... **31**
Figure S47. ^{1}H NMR spectrum (600 MHz) of **7** in $CDCl_3$ 31
Figure S48. Localized amplified ^{1}H NMR spectrum (600 MHz) of **7** in $CDCl_3$ 31
Figure S49. ^{13}C NMR spectrum (150 MHz) of **7** in $CDCl_3$ 32
Figure S50. HSQC spectrum (600 MHz) of **7** in $CDCl_3$ 32
Figure S51. Localized amplified HSQC spectrum (600 MHz) of **7** in $CDCl_3$ 33
Figure S52. HMBC spectrum (600 MHz) of **7** in $CDCl_3$ 33
Figure S53. ^{1}H–^{1}H COSY spectrum (600 MHz) of **7** in $CDCl_3$ 34
Figure S54. NOESY spectrum (600 MHz) of **7** in $CDCl_3$ 35
Figure S55. HR-ESIMS spectrum of **7** 36
Figure S56. ECD and UV spectra of **7** 36
7. QM-NMR calculation and DP4+ analysis of 2-5 .... **37**
Figure S57. Structures of studied isomers for compound **2** 37
Figure S58. DP4+ results obtained using experimental NMR data of **2** *versus* calculated NMR data of isomers 1 [(11*S**,12*S**)-**2**] and 2 [(11*R**,12*S**)-**2**] 37
Figure S59. Structures of studied isomers for compound **3** 38
Figure S60. DP4+ results obtained using experimental NMR data of **3** *versus* calculated NMR data of isomers 1 [(1*R**,3*R**,4*R**,15*S**)-**3**], 2 [(1*R**,3*S**,4*R**,15*S**)-**3**], 3 [(1*R**,3*S**,4*S**,15*R**)-**3**] and 4 [(1*R**,3*R**,4*S**,15*R**)-**3**] 38
Figure S61. Structures of studied isomers for compound **4** 38
Figure S62. DP4+ results obtained using experimental NMR data of **4** *versus* calculated NMR data of isomers 1 [(1*R**,4*R**,12*R**)-**4**] and 2 [(1*S**,4*R**,12*S**)-**4**] 38
Figure S63. Structures of studied isomers for compound **5** 39
Figure S64. DP4+ results obtained using experimental NMR data of **5** *versus* calculated NMR data of isomers 1 [(1*R**,3*R**,4*R**,7*S**)-**5**], 2 (1*S**,3*S**,4*R**,7*S**)-**5**], 3 (1*R**,3*R**,4*S**,7*R**)-**5**] and 4 (1*S**,3*R**,4*R**,7*S**)-**5**] 39
8. TDDFT-ECD calculations of 2-5 and 7 .... **40**
Figure S65. Re-optimized conformers of (11*S*,12*S*)-**2** calculated at the B3LYP/6-311G(d,p) level 40
Table S1. Cartesian coordinates for the re-optimized conformers of (11*S*,12*S*)-**2** 41
Figure S66. Re-optimized conformers of (1*R*,3*S*,4*S*,15*R*)-**3** calculated at the B3LYP/6-311G(d,p) level 47
Table S2. Cartesian coordinates for the re-optimized conformers of (1*R*,3*S*,4*S*,15*R*)-**3** . 47
Figure S67. Re-optimized conformers of (1*S*,4*R*,12*S*)-**4** calculated at the B3LYP/6-311G(d,p) level 52

Table S3. Cartesian coordinates for the re-optimized conformers of (1*S*,4*R*,12*S*)-**4**....... 52
Figure S68. Re-optimized conformers of (1*R*,3*R*,4*S*,7*R*)-**5** calculated at the B3LYP/6-311G(d,p) level.. 53
Table S4. Cartesian coordinates for the re-optimized conformers of (1*R*,3*R*,4*S*,7*R*)-**5**... 53
Figure S69. Re-optimized conformers of (1*S*,2*R*,5*R*,12*S*)-**7** calculated at the B3LYP/6-311G(d,p) level.. 56
Table S5. Cartesian coordinates for the re-optimized conformers of (1*S*,2*R*,5*R*,12*S*)-**7** . 56
9. Origin spectra of known compounds 6 and 8-16. 61
Figure S70. ^{1}H NMR spectrum (500 MHz) of **6** in $CDCl_3$.. 61
Figure S71. ^{13}C NMR spectrum (125 MHz) of **6** in $CDCl_3$... 62
Figure S72. ^{1}H NMR spectrum (500 MHz) of **8** in $CDCl_3$.. 62
Figure S73. ^{13}C NMR spectrum (125 MHz) of **8** in $CDCl_3$... 63
Figure S74. ^{1}H NMR spectrum (500 MHz) of **9** in $CDCl_3$.. 63
Figure S75. ^{13}C NMR spectrum (125 MHz) of **9** in $CDCl_3$... 64
Figure S76. ^{1}H NMR spectrum (500 MHz) of **10** in $CDCl_3$.. 64
Figure S77. ^{13}C NMR spectrum (125 MHz) of **10** in $CDCl_3$... 65
Figure S78. ^{1}H NMR spectrum (500 MHz) of **11** in $CDCl_3$.. 65
Figure S79. ^{13}C NMR spectrum (125 MHz) of **11** in $CDCl_3$... 66
Figure S80. ^{1}H NMR spectrum (500 MHz) of **12** in $CDCl_3$.. 66
Figure S81. ^{13}C NMR spectrum (125 MHz) of **12** in $CDCl_3$... 67
Figure S82. ^{1}H NMR spectrum (500 MHz) of **13** in $CDCl_3$.. 67
Figure S83. ^{13}C NMR spectrum (125 MHz) of **13** in $CDCl_3$... 68
Figure S84. ^{1}H NMR spectrum (500 MHz) of **14** in $CDCl_3$.. 68
Figure S85. ^{13}C NMR spectrum (125 MHz) of **14** in $CDCl_3$... 69
Figure S86. ^{1}H NMR spectrum (500 MHz) of **15** in $CDCl_3$.. 69
Figure S87. ^{13}C NMR spectrum (125 MHz) of **15** in $CDCl_3$... 70
Figure S88. ^{1}H NMR spectrum (500 MHz) of **16** in $CDCl_3$.. 70
Figure S89. ^{13}C NMR spectrum (125 MHz) of **16** in $CDCl_3$... 71
10. Raw experimental bioactivity data. 71

1. Original spectra of **1**.

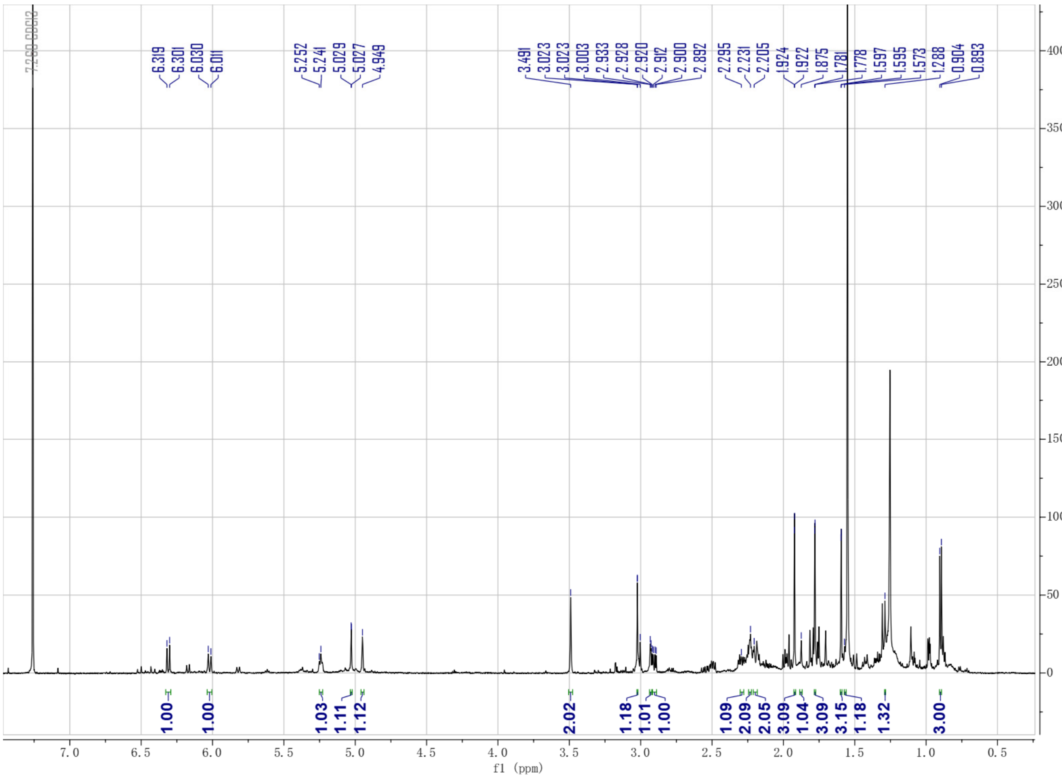

Figure S1. ^{1}H NMR spectrum (600 MHz) of **1** in $CDCl_3$

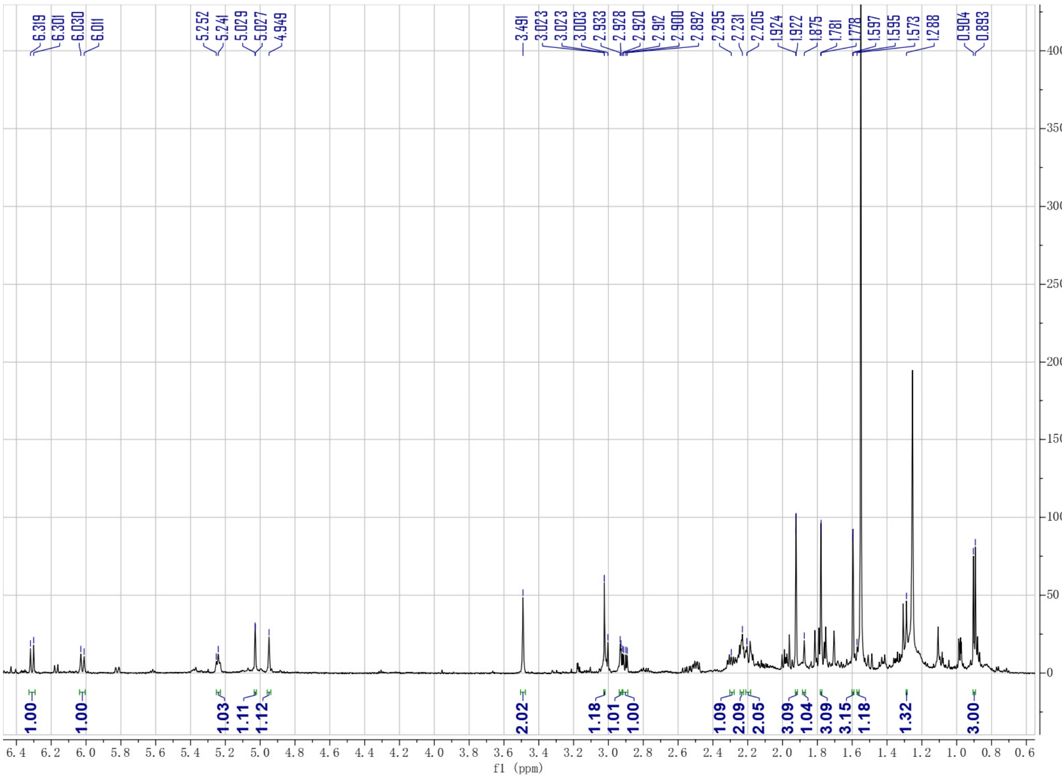

Figure S2. Localized amplified ^{1}H NMR spectrum (600 MHz) of **1** in $CDCl_3$

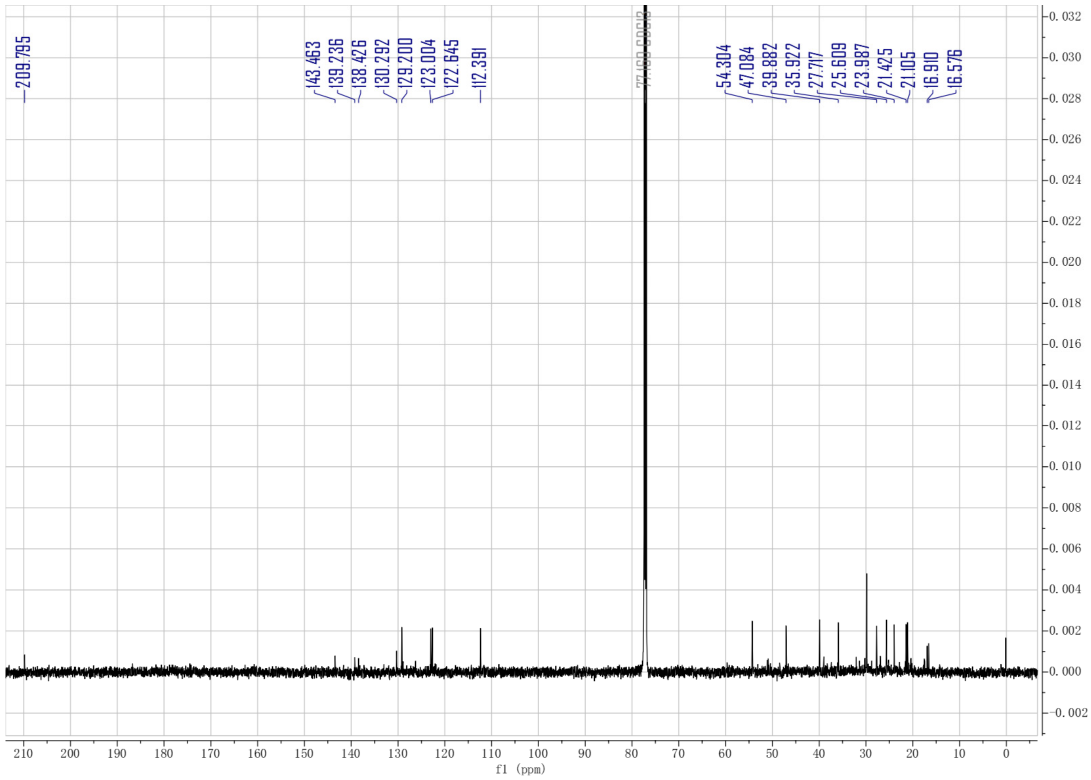

Figure S3. ^{13}C NMR spectrum (150 MHz) of **1** in $CDCl_3$

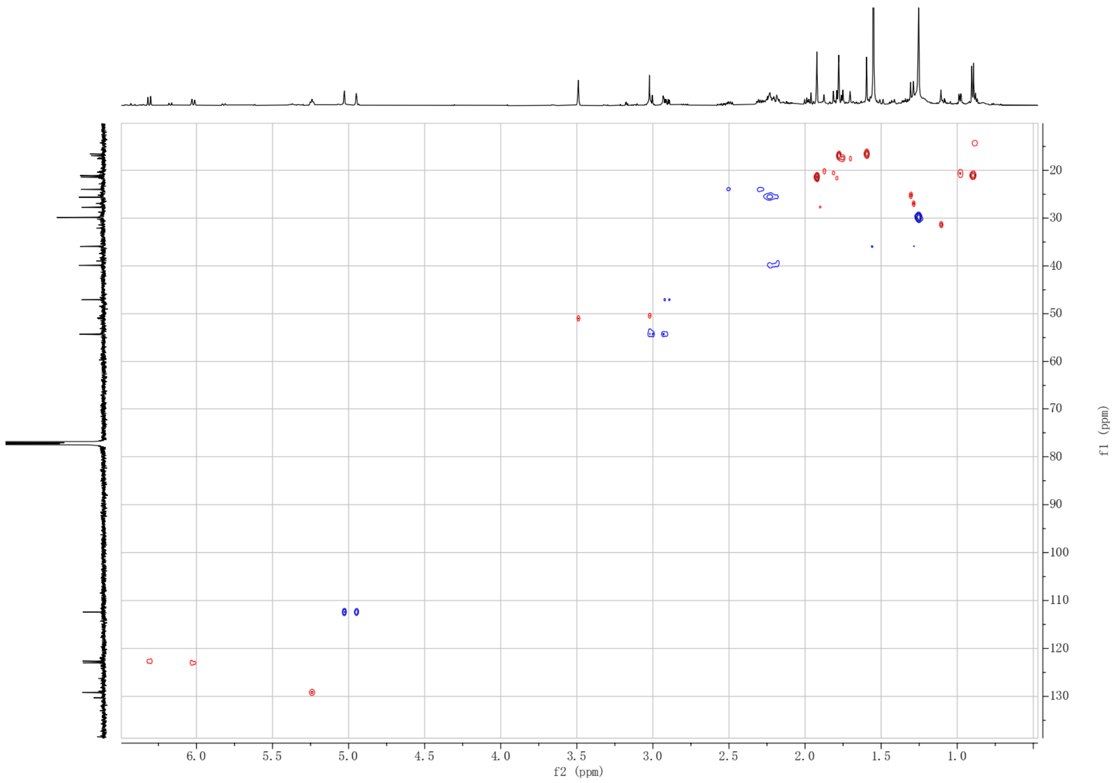

Figure S4. HSQC spectrum (600 MHz) of **1** in $CDCl_3$

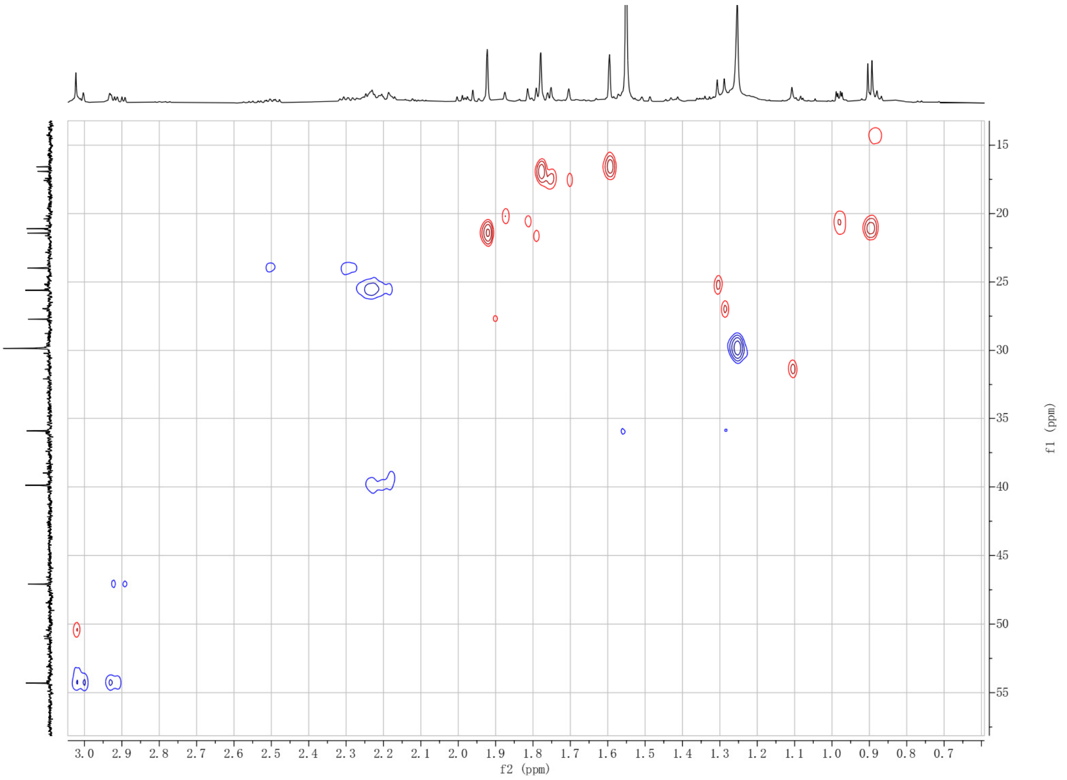

Figure S5. Localized amplified HSQC spectrum (600 MHz) of **1** in $CDCl_3$

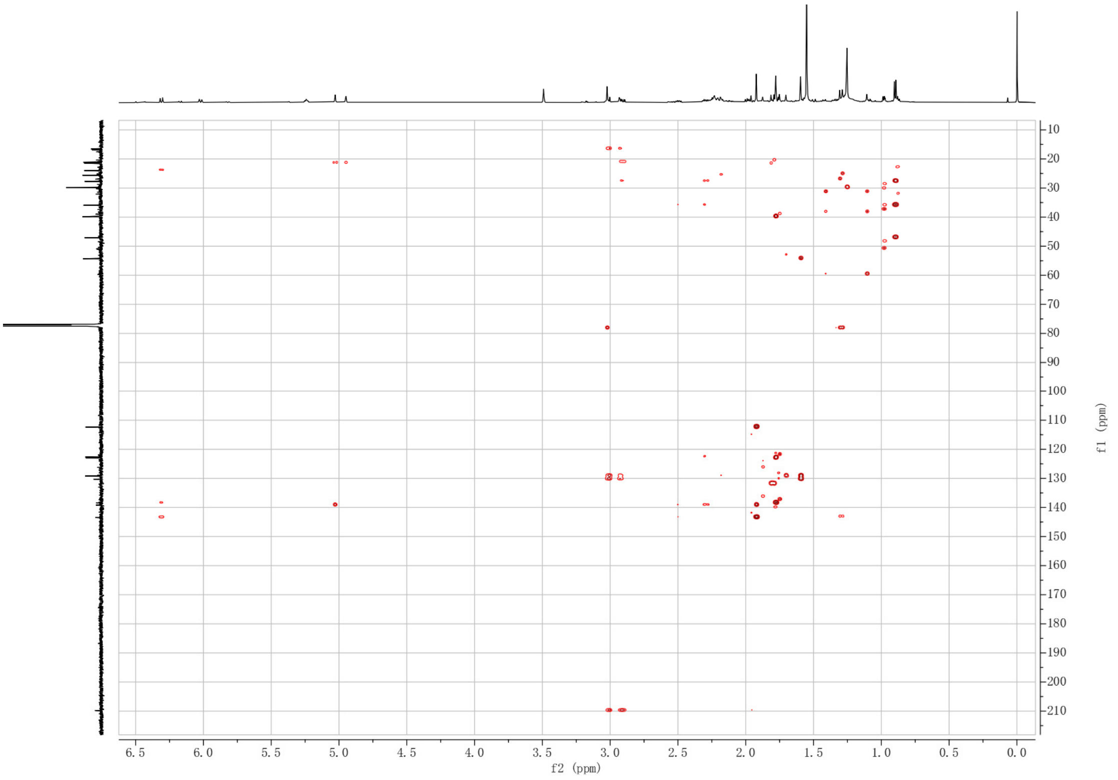

Figure S6. HMBC spectrum (600 MHz) of **1** in $CDCl_3$

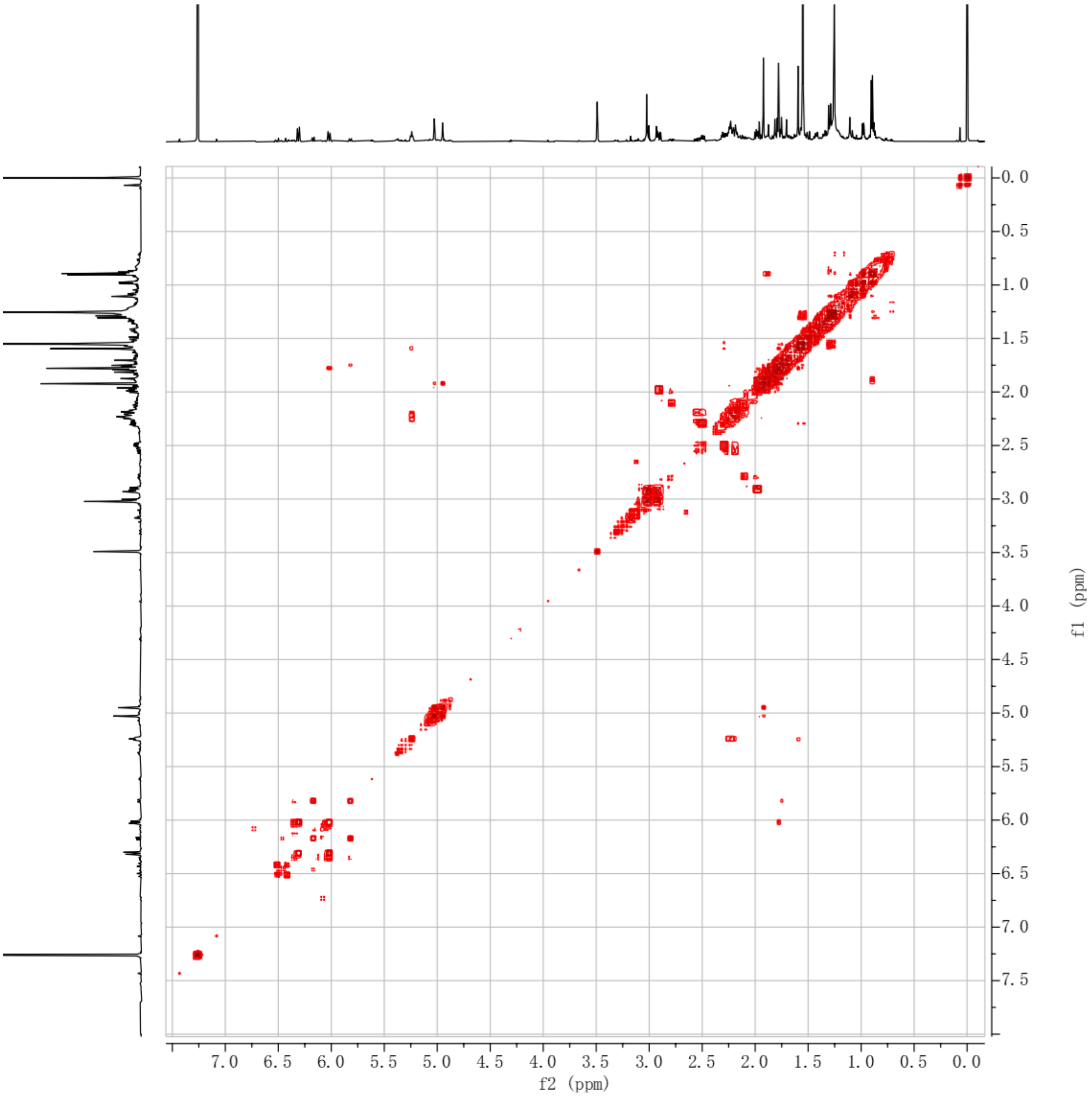

Figure S7. ^{1}H-^{1}H COSY spectrum (600 MHz) of **1** in $CDCl_3$

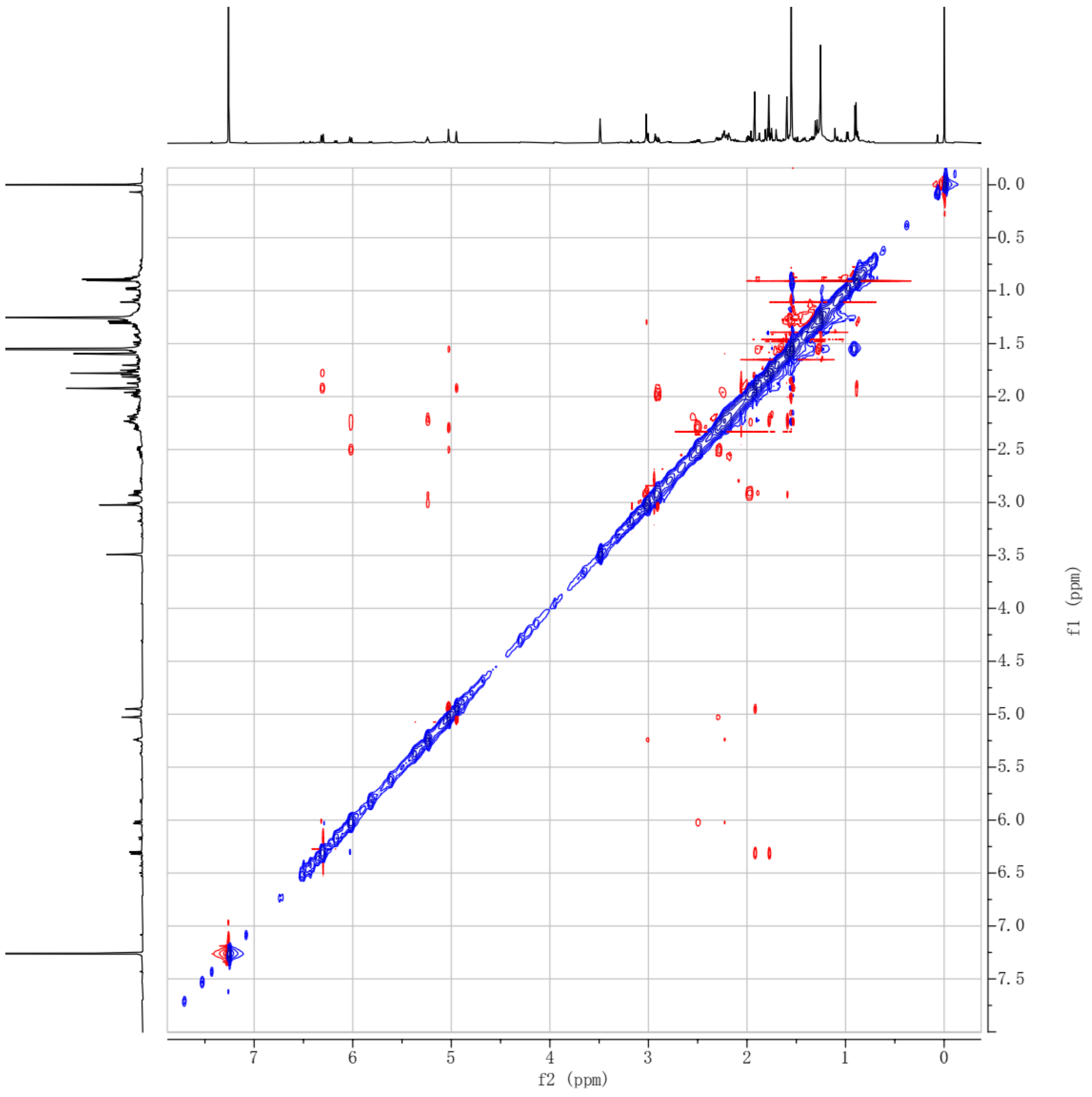

Figure S8. NOESY spectrum (600 MHz) of **1** in $CDCl_3$

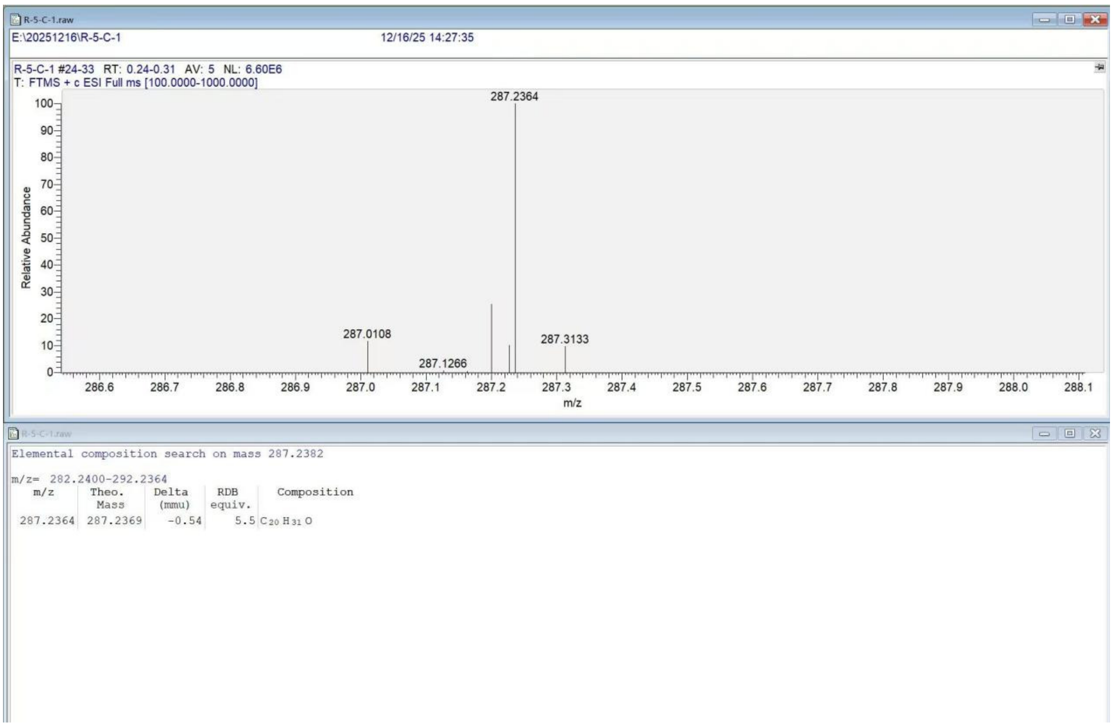

Measured *m/z*	Cal *m/z*	Error (mmu)	Ion Formula	Ion
287.2364	287.2369	-0.54	$C_{20}H_{31}O$	$[M+H]^+$

Figure S9. HR-ESIMS spectrum of **1**

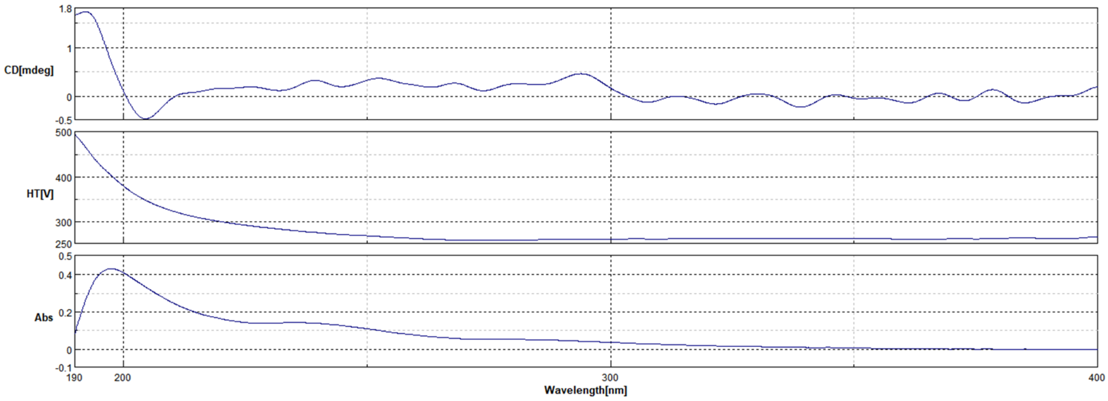

Figure S10. ECD and UV spectra of **1**

2. Original spectra of **2**.

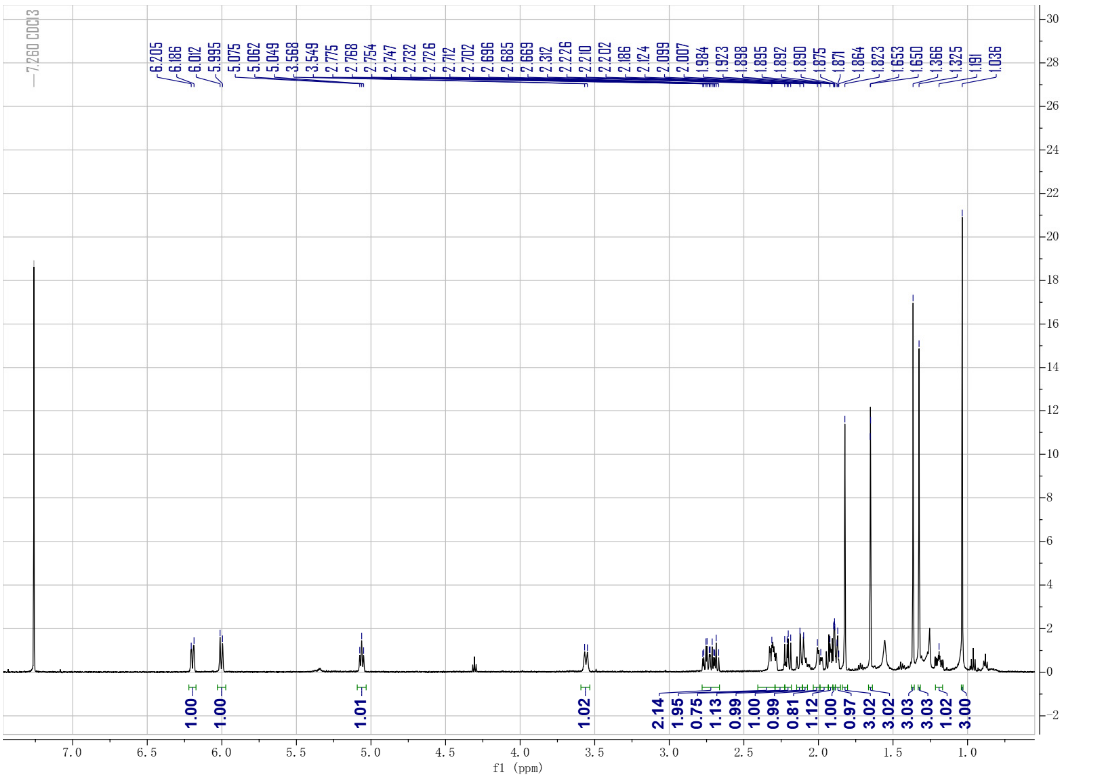

Figure S11. ^{1}H NMR spectrum (600 MHz) of **2** in $CDCl_3$

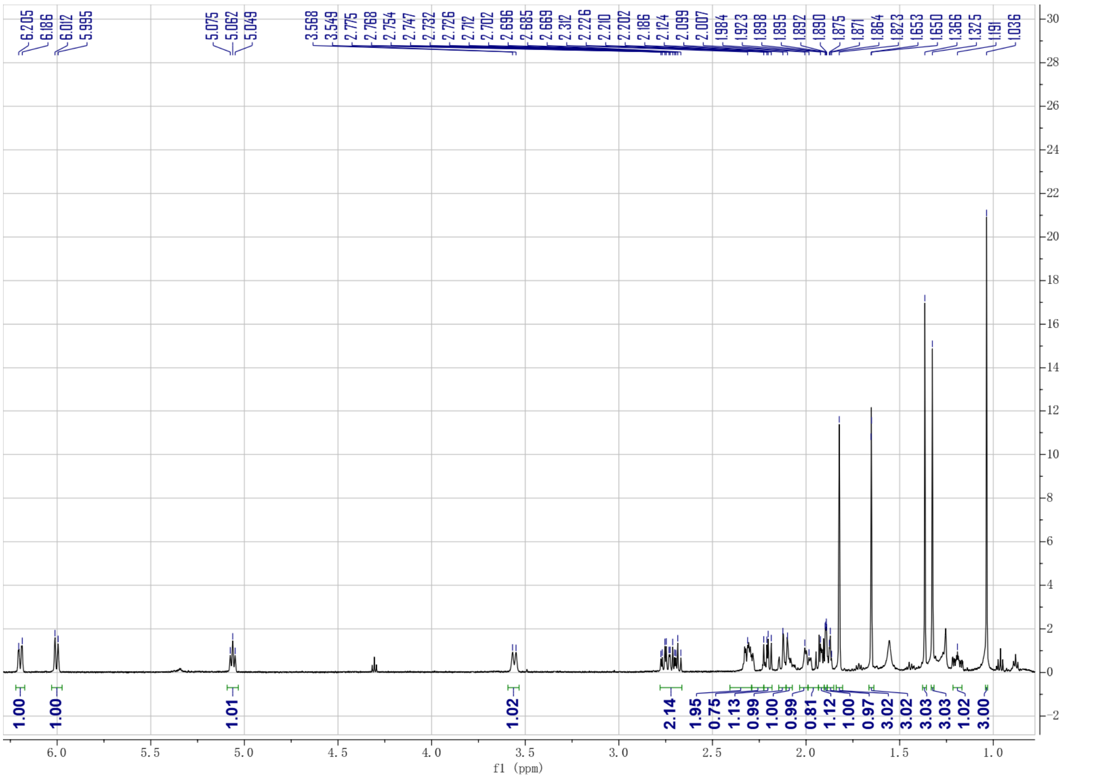

Figure S12. Localized amplified ^{1}H NMR spectrum (600 MHz) of **2** in $CDCl_3$

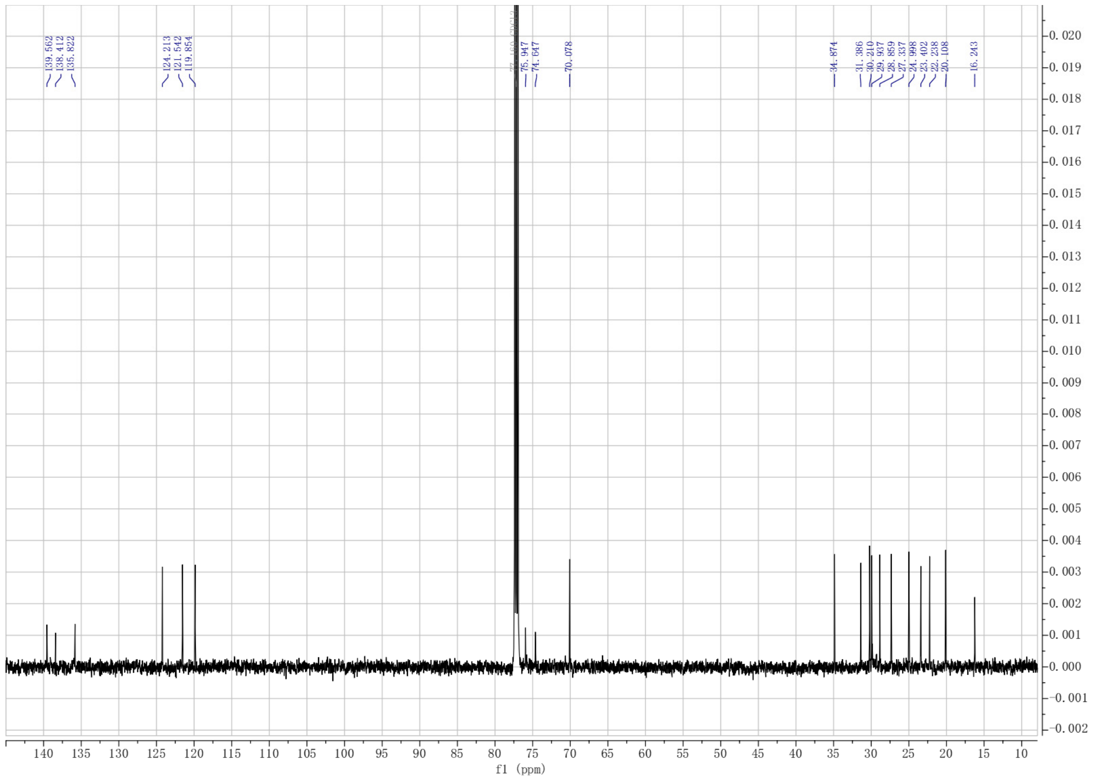

Figure S13. ^{13}C NMR spectrum (150 MHz) of **2** in $CDCl_3$

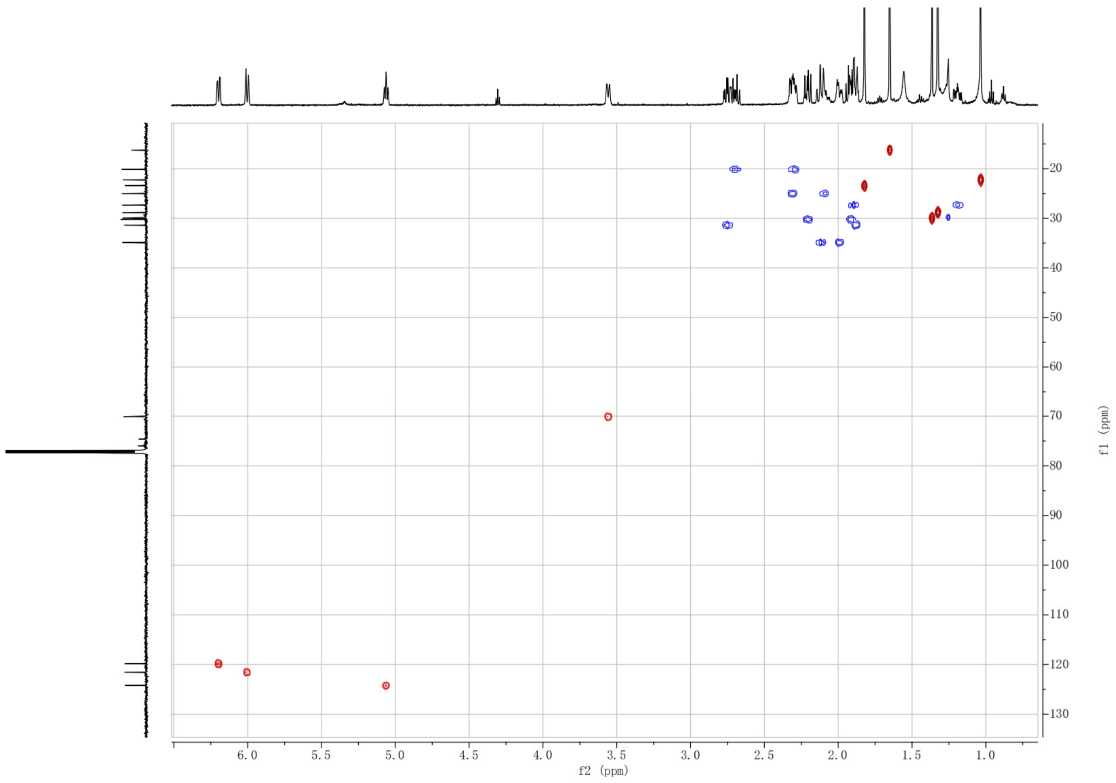

Figure S14. HSQC spectrum (600 MHz) of **2** in $CDCl_3$

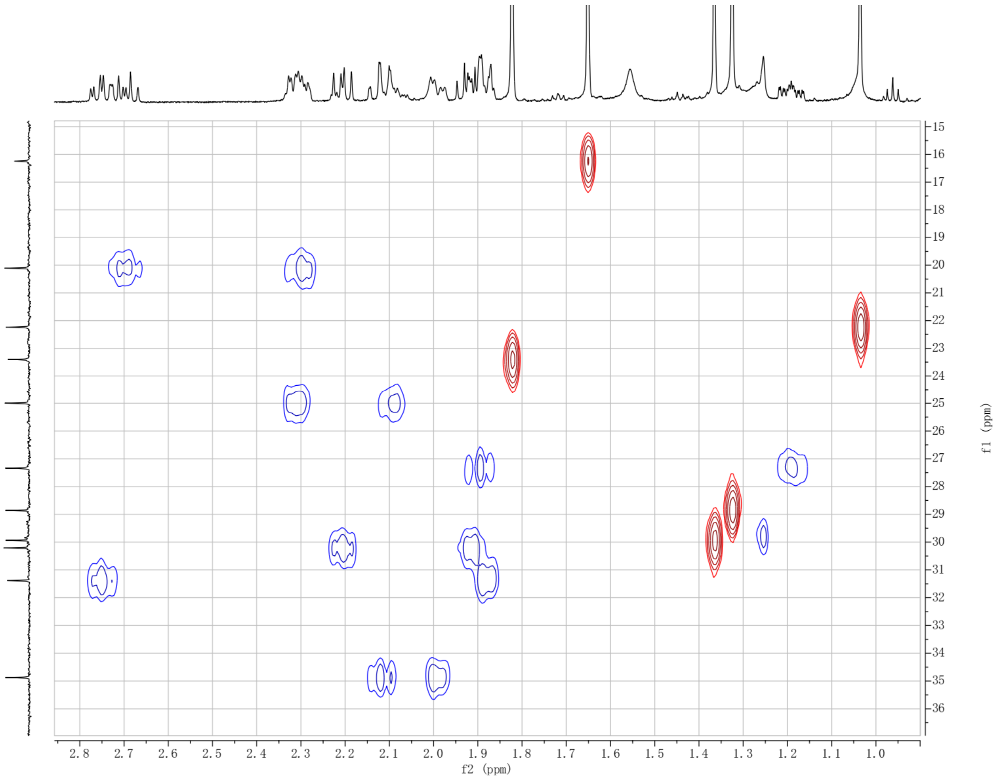

Figure S15. Localized amplified HSQC spectrum (600 MHz) of **2** in $CDCl_3$

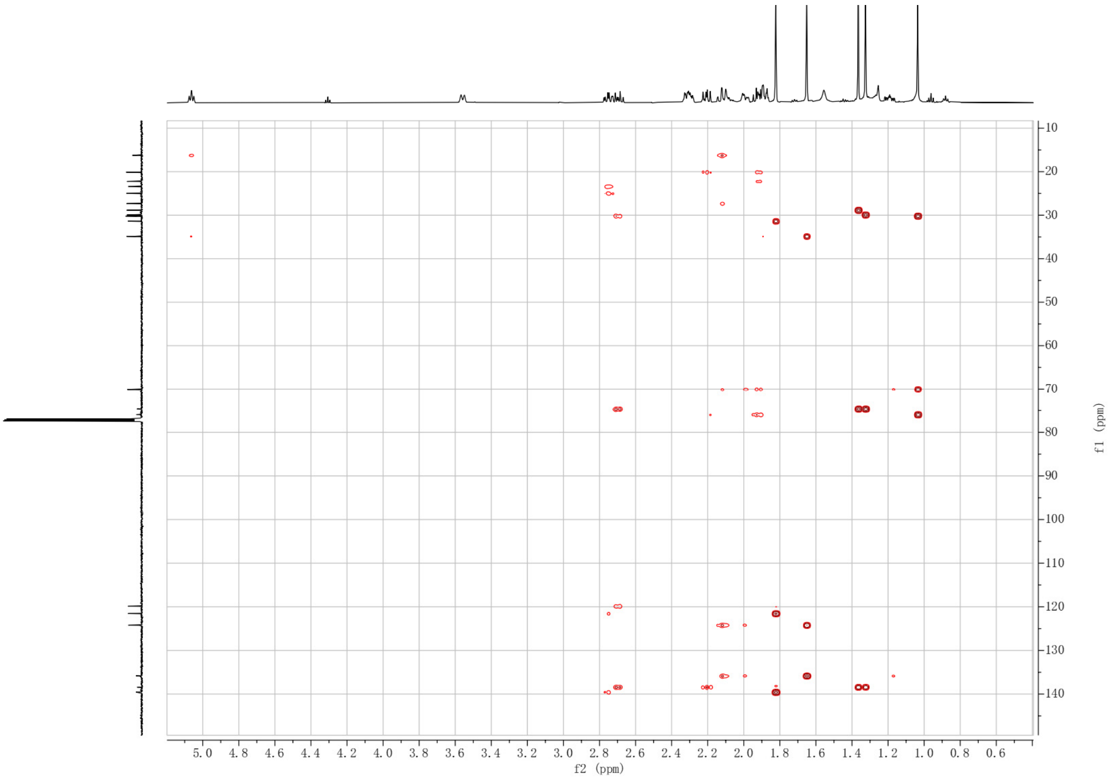

Figure S16. HMBC spectrum (600 MHz) of **2** in $CDCl_3$

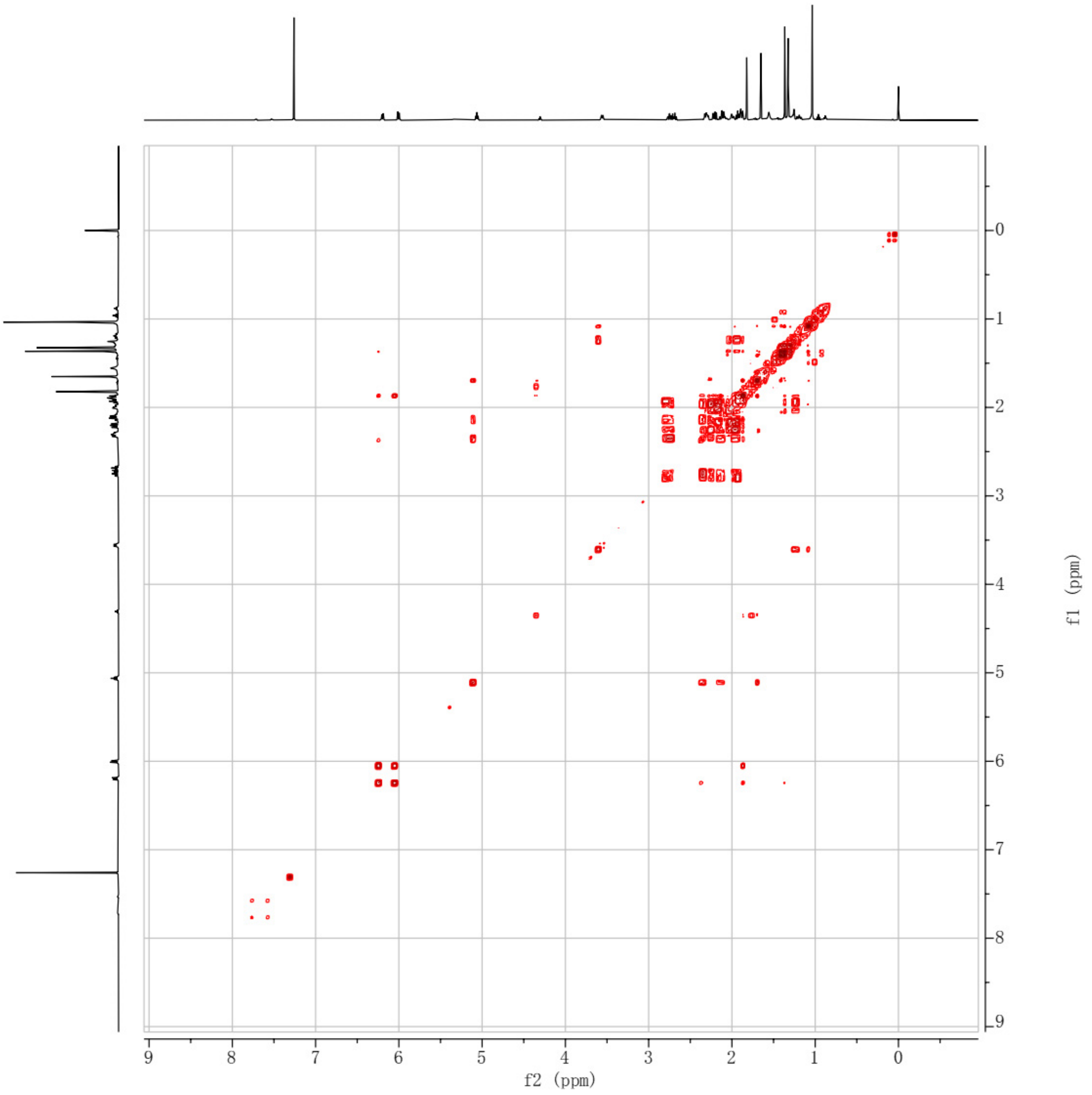

Figure S17. ^{1}H–^{1}H COSY spectrum (600 MHz) of **2** in $CDCl_3$

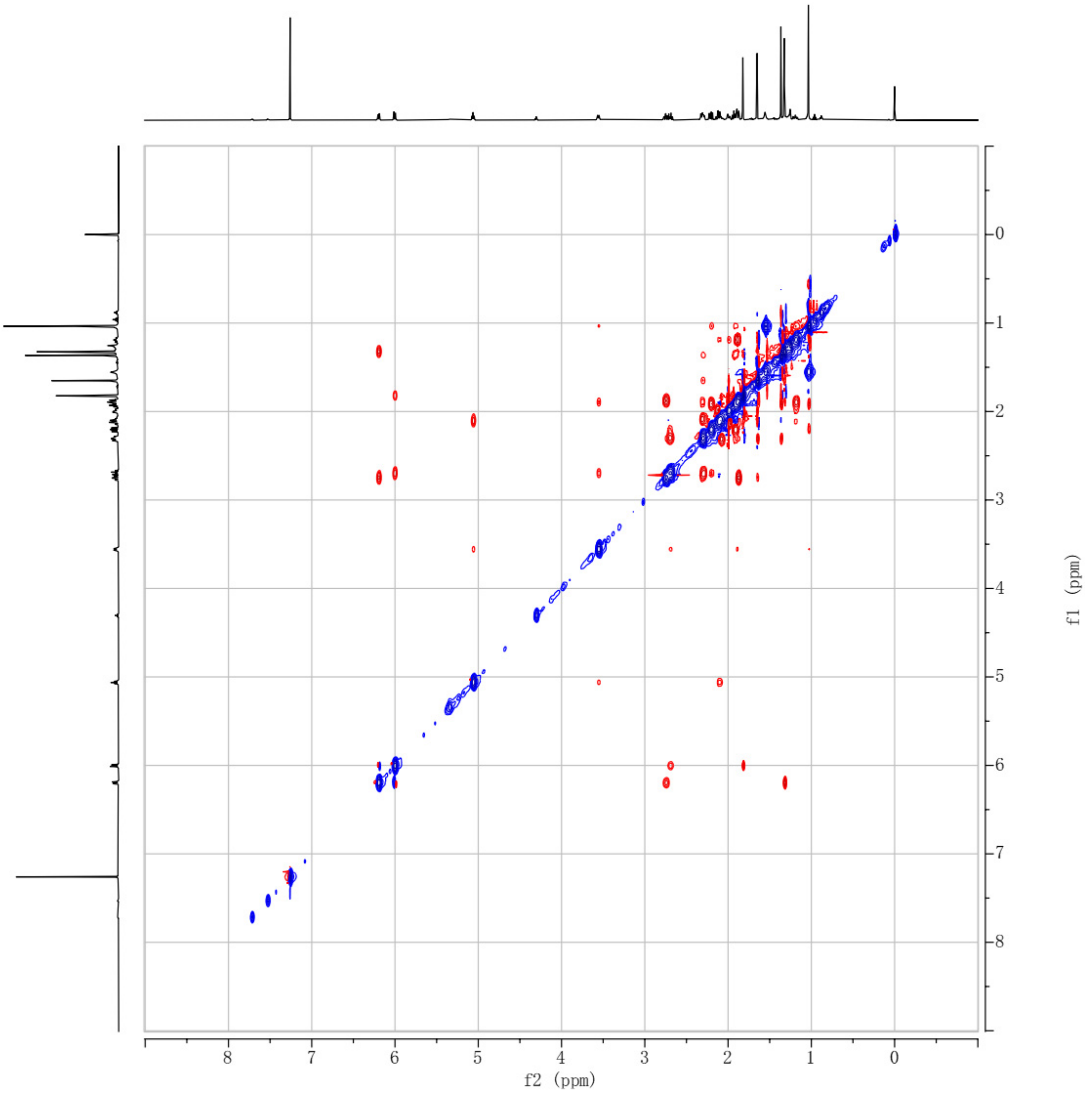

Figure S18. NOESY spectrum (600 MHz) of **2** in $CDCl_3$

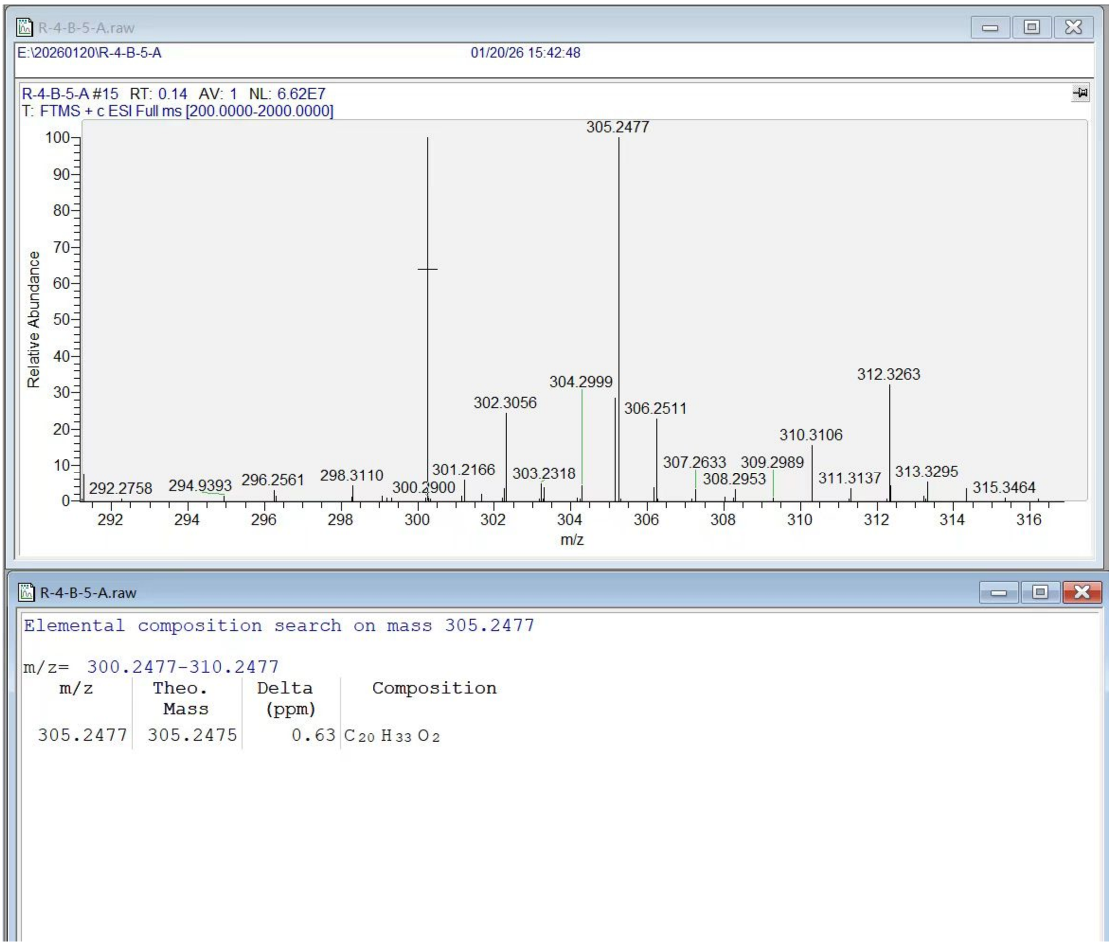

Measured *m/z*	Cal *m/z*	Error (mmu)	Ion Formula	Ion
305.2477	305.2475	0.63	$C_{20}H_{33}O_2$	$[M+H]^+$

Figure S19. HR-ESIMS spectrum of **2**

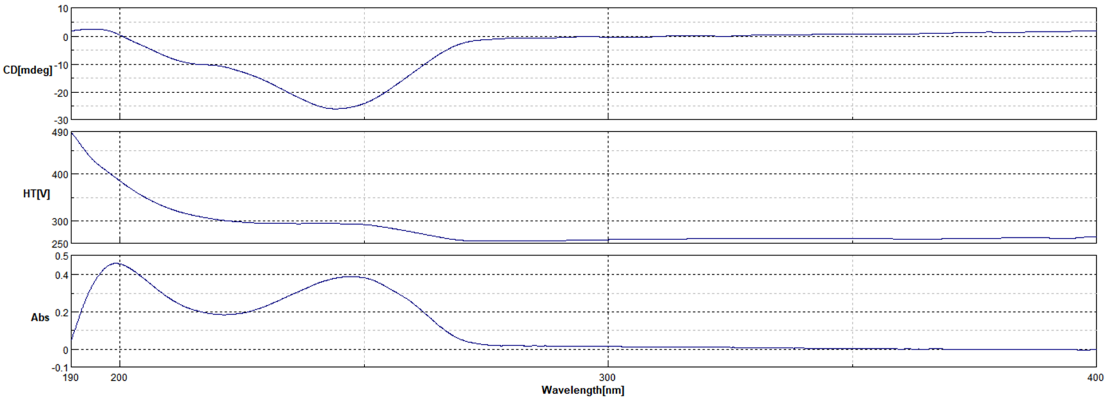

Figure S20. ECD and UV spectra of **2**

3. Original spectra of **3**.

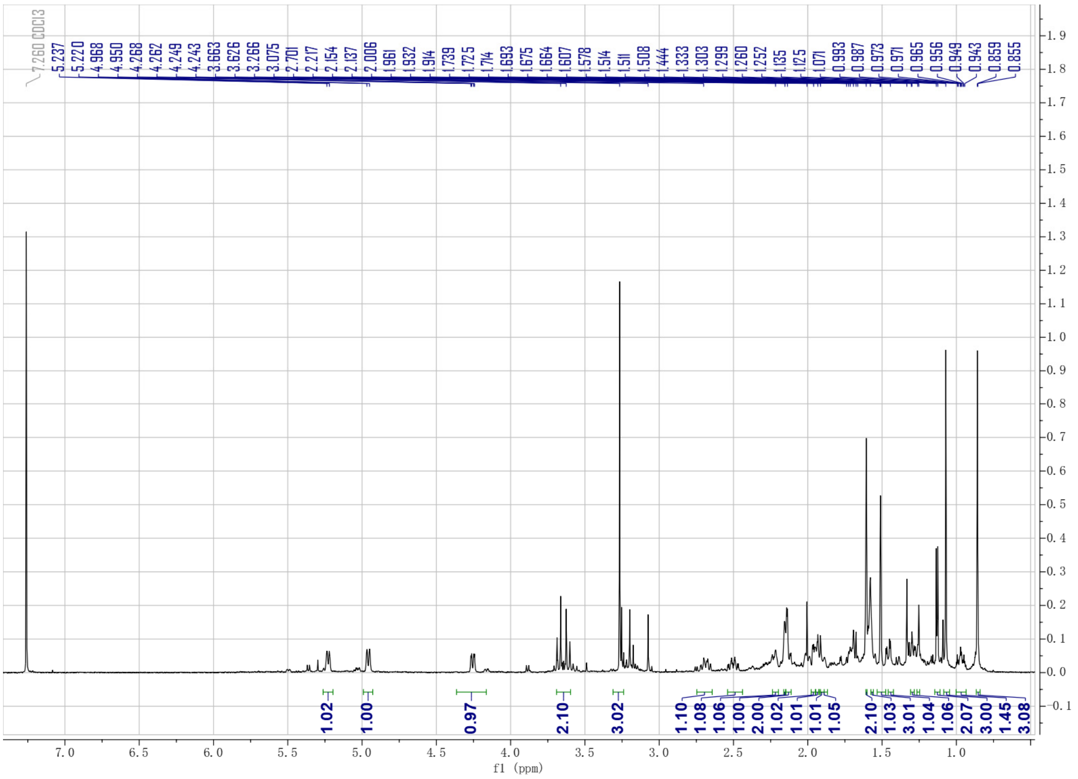

Figure S21. ^{1}H NMR spectrum (600 MHz) of **3** in $CDCl_3$

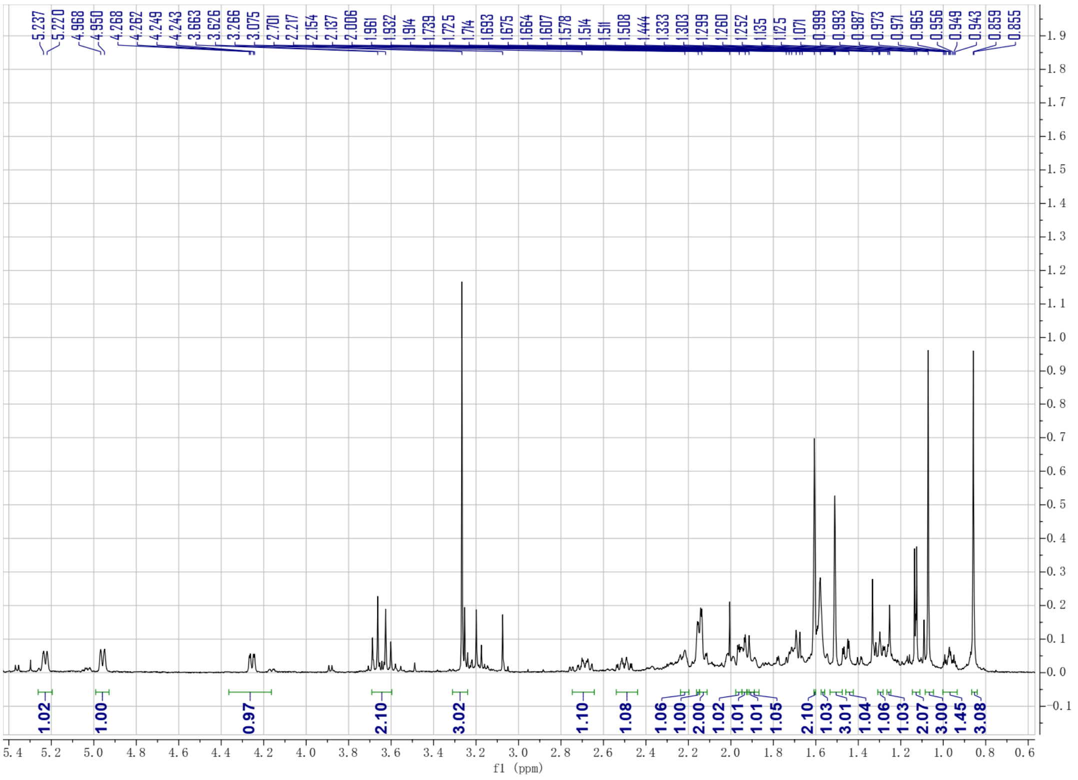

Figure S22. Localized amplified ^{1}H NMR spectrum (600 MHz) of **3** in $CDCl_3$

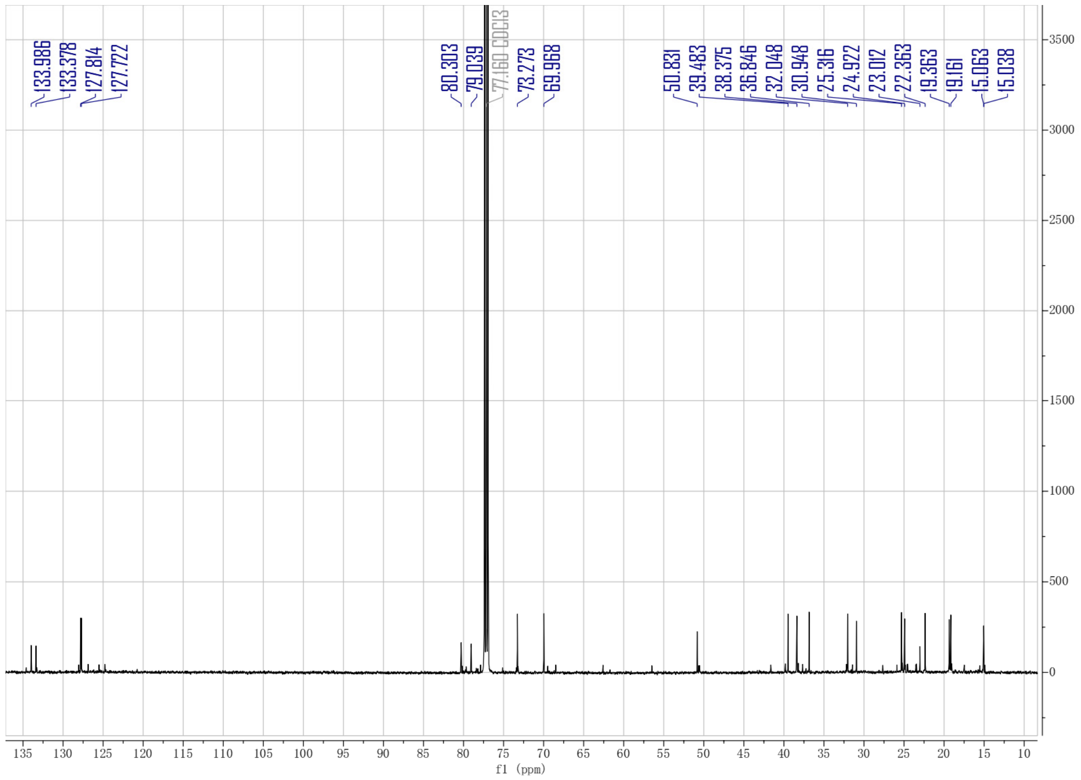

Figure S23. ^{13}C NMR spectrum (150 MHz) of **3** in $CDCl_3$

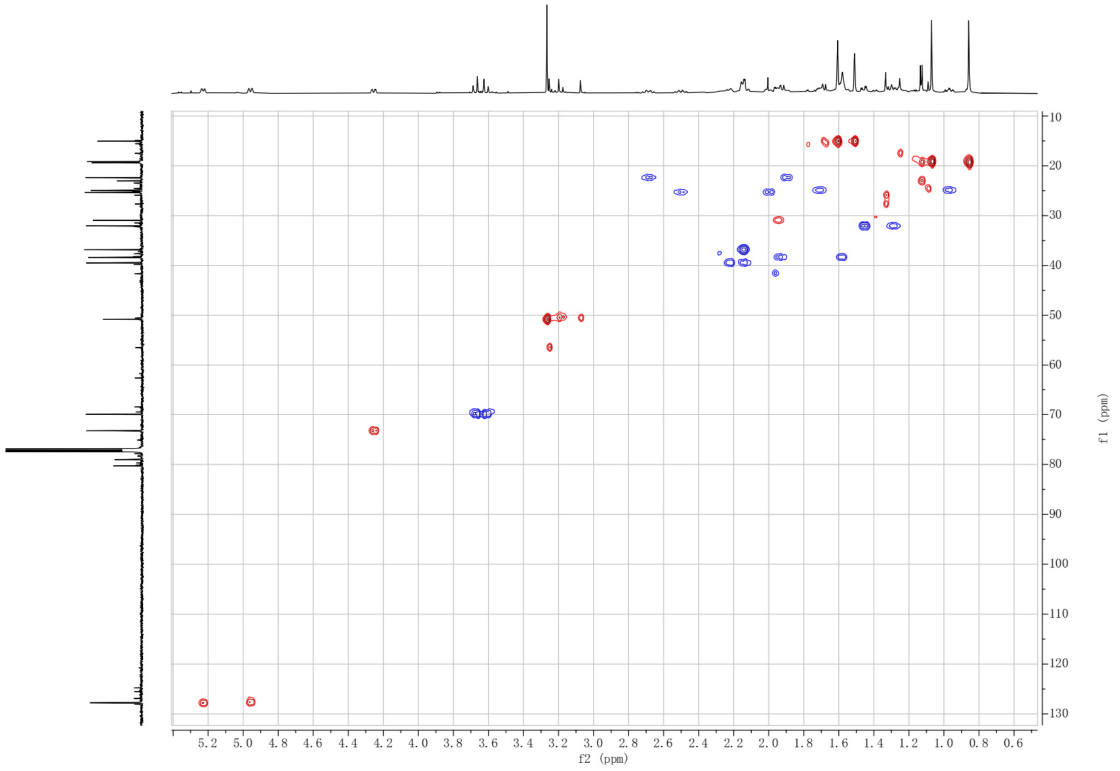

Figure S24. HSQC spectrum (600 MHz) of **3** in $CDCl_3$

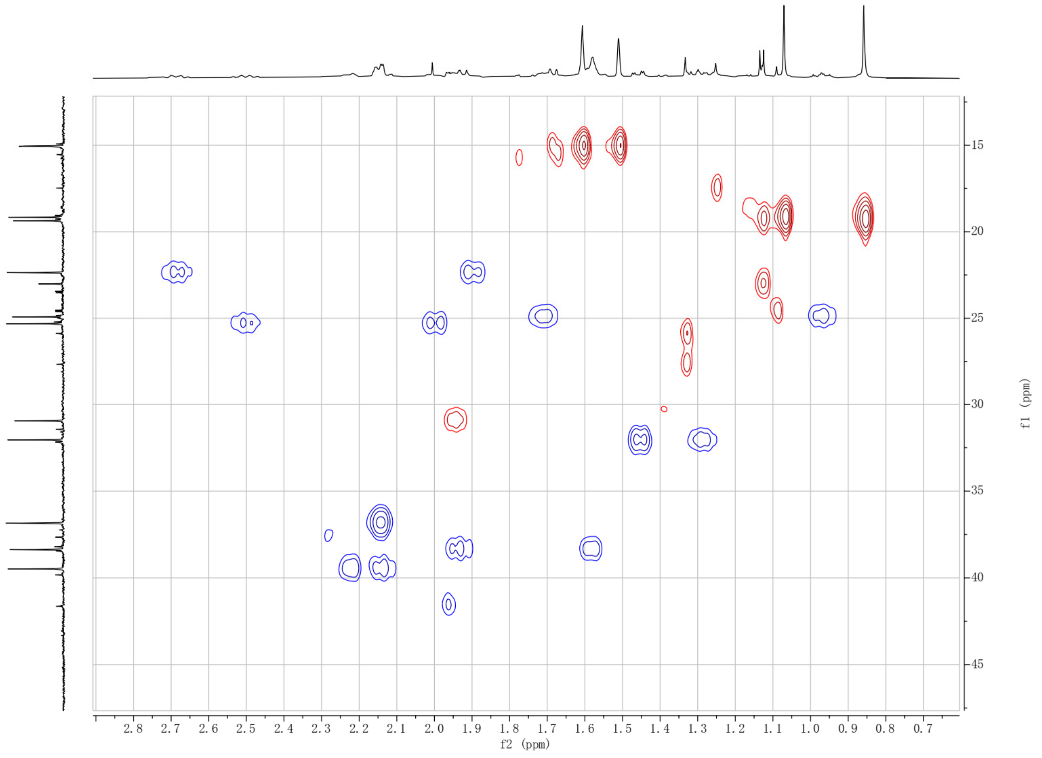

Figure S25. Localized amplified HSQC spectrum (600 MHz) of **3** in $CDCl_3$

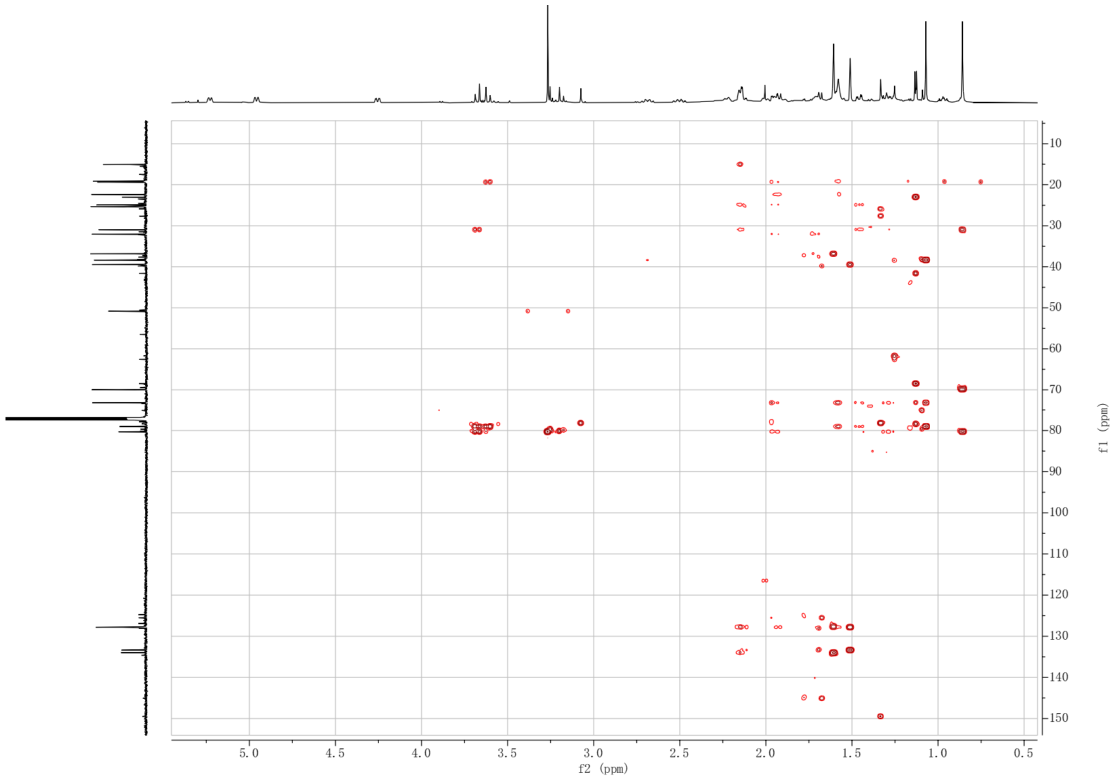

Figure S26. HMBC spectrum (600 MHz) of **3** in $CDCl_3$

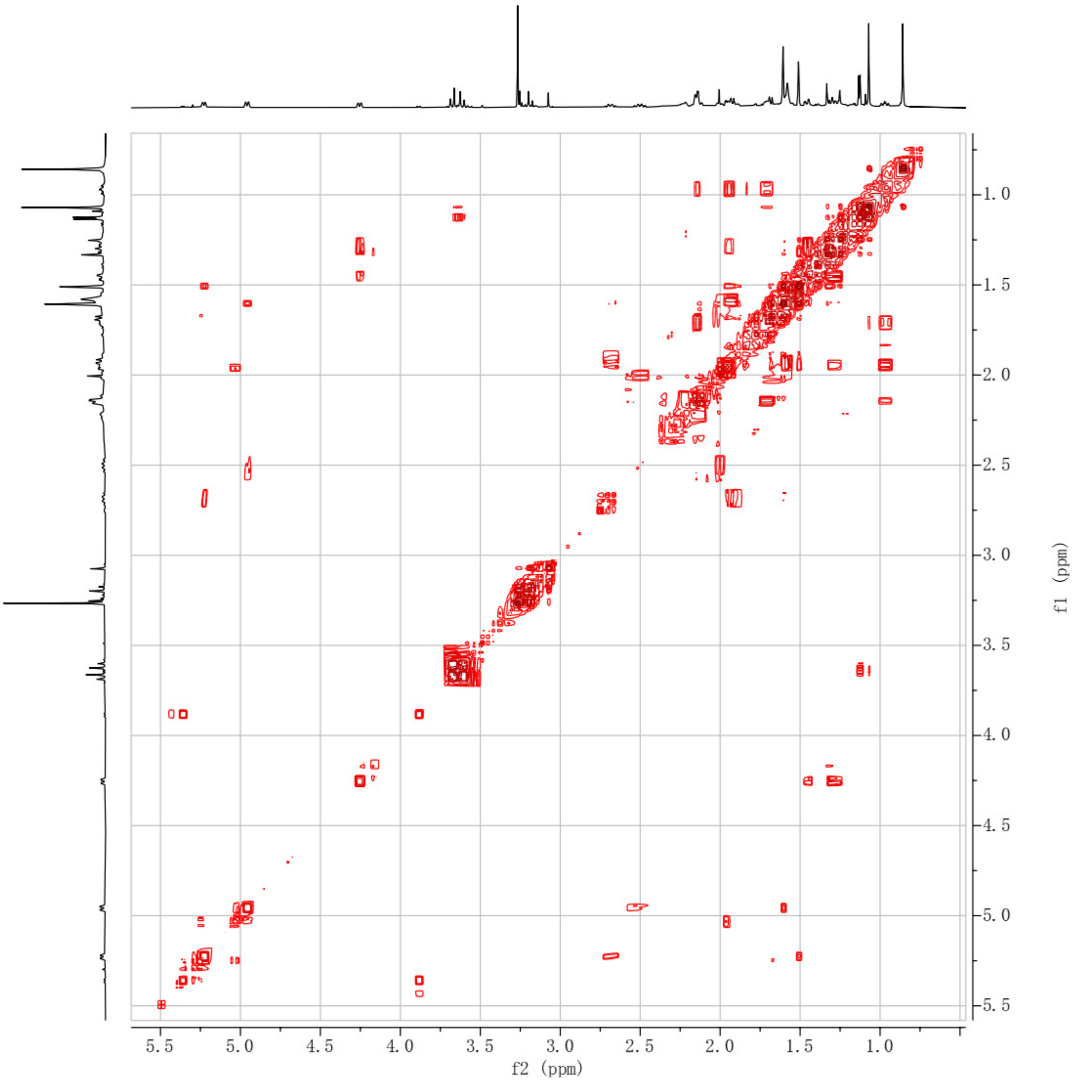

Figure S27. ^{1}H–^{1}H COSY spectrum (600 MHz) of **3** in $CDCl_3$

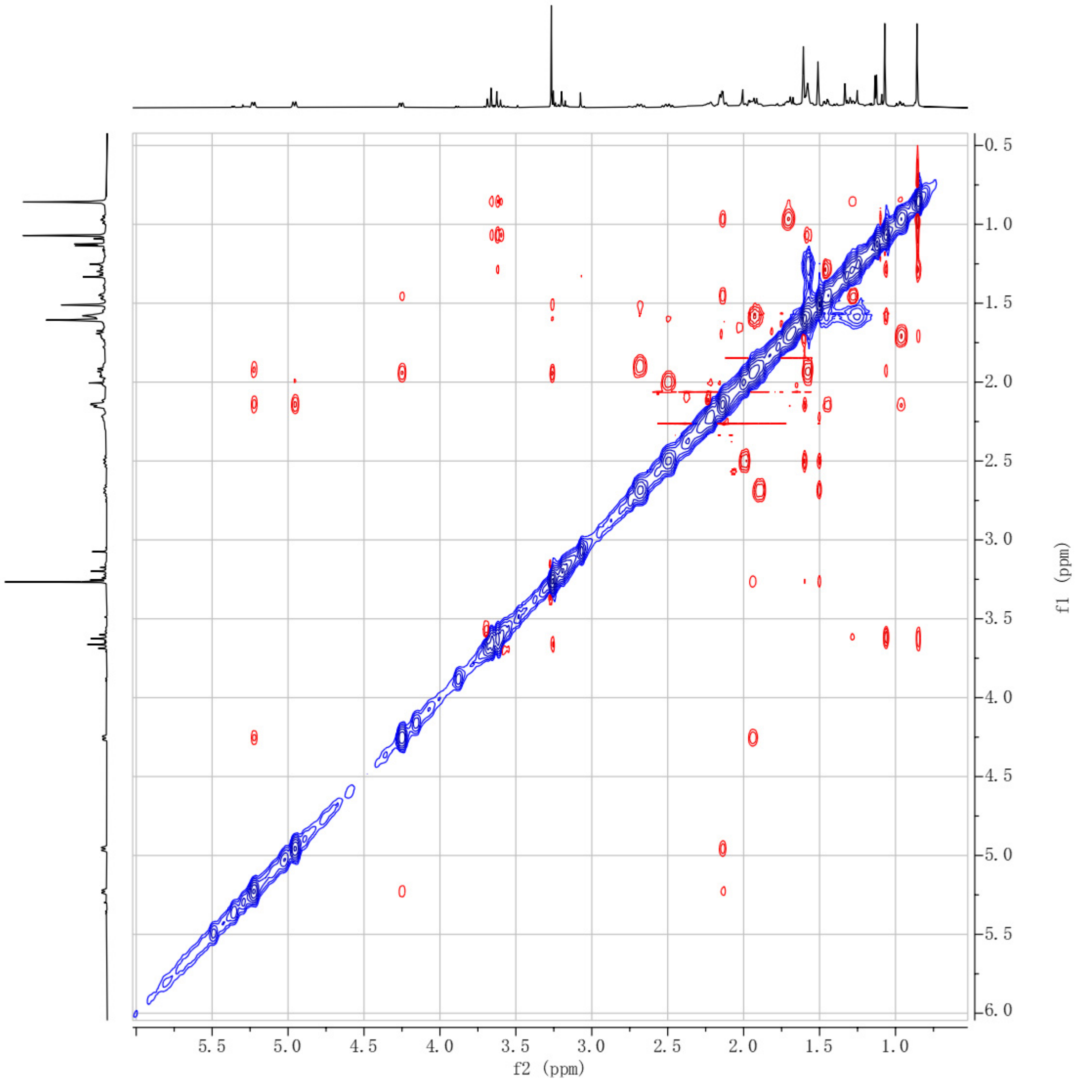

Figure S28. NOESY spectrum (600 MHz) of **3** in $CDCl_3$

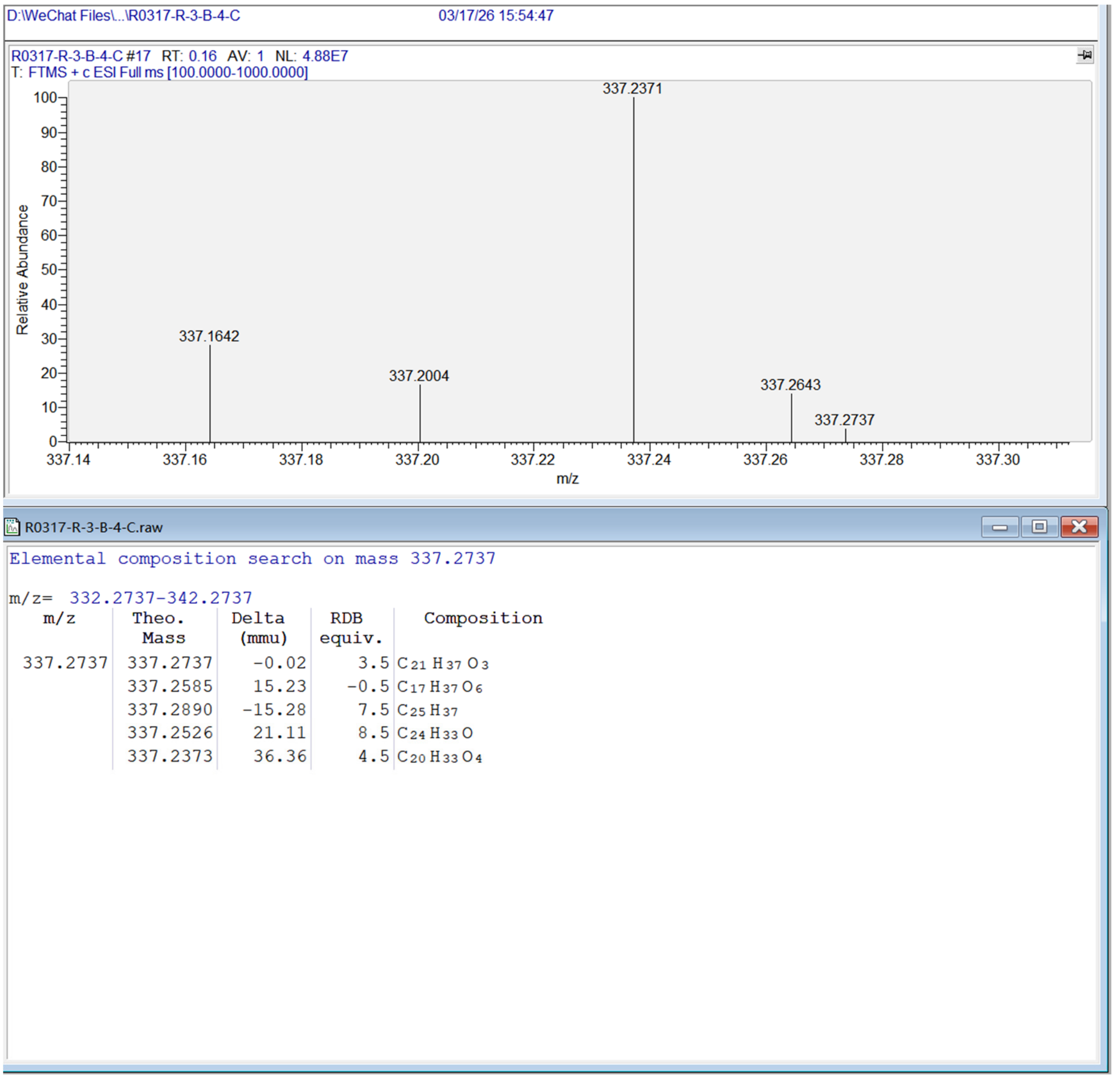

Measured *m/z*	Cal *m/z*	Error (mmu)	Ion Formula	Ion
337.2737	337.2737	-0.02	$C_{21}H_{37}O_3$	$[M+H]^+$

Figure S29. HR-ESIMS spectrum of **3**

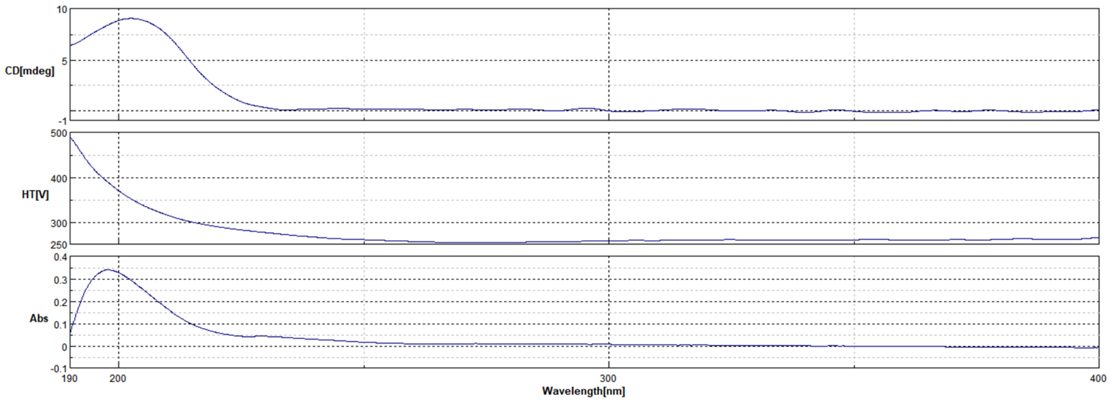

Figure S30. ECD and UV spectra of **3**

4. Original spectra of **4**.

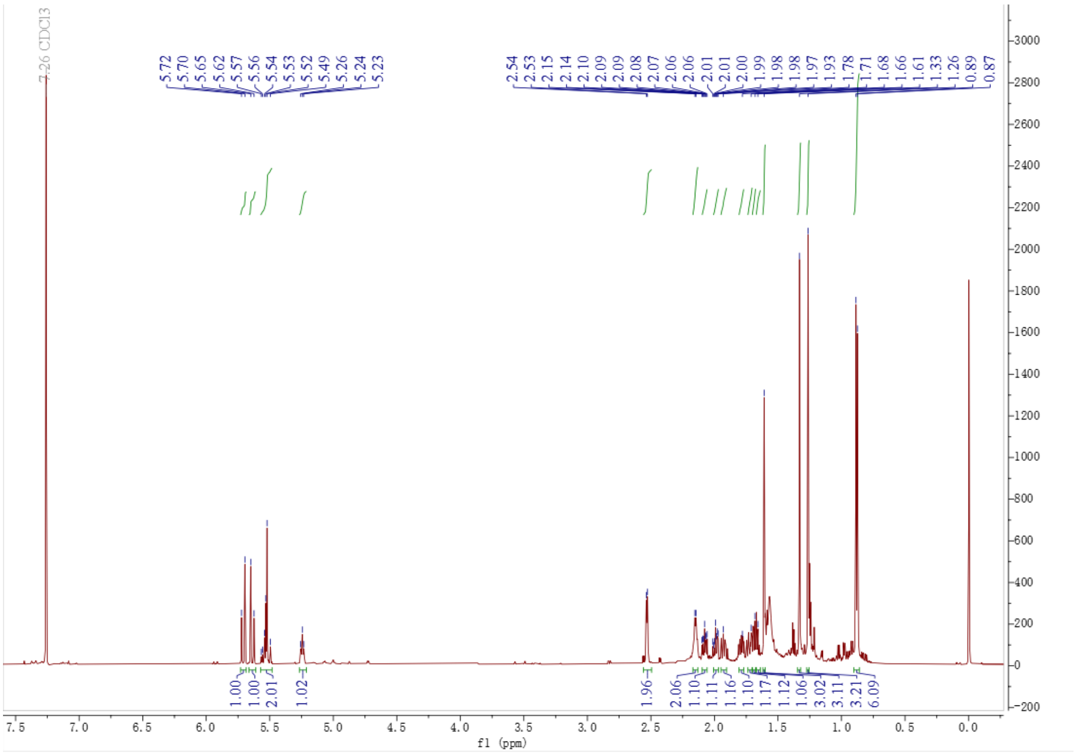

Figure S31. ^{1}H NMR spectrum (600 MHz) of **4** in $CDCl_3$

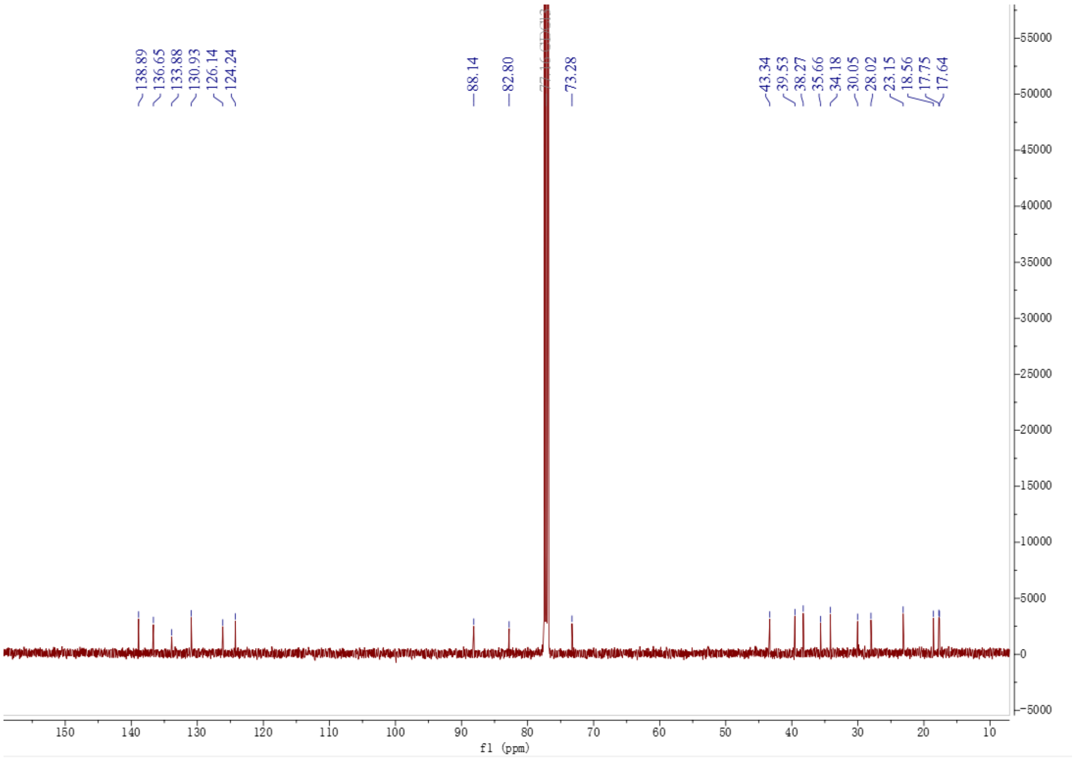

Figure S32. ^{13}C NMR spectrum (150 MHz) of **4** in $CDCl_3$

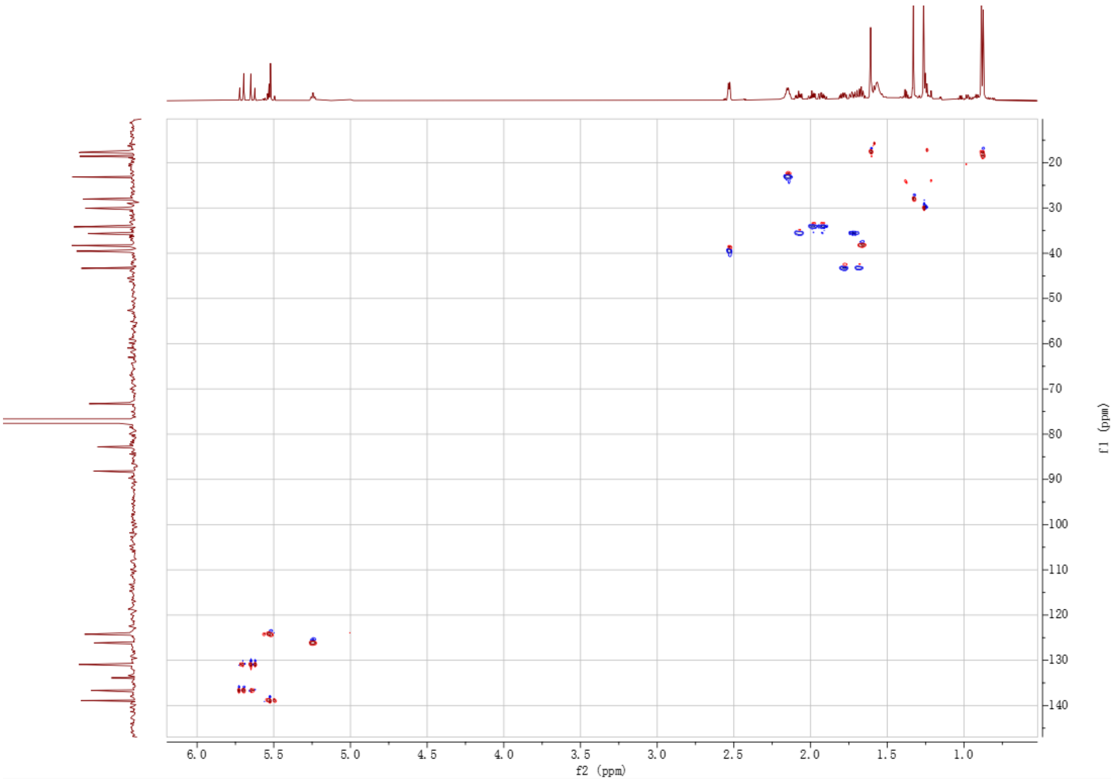

Figure S33. HSQC spectrum (600 MHz) of **4** in $CDCl_3$

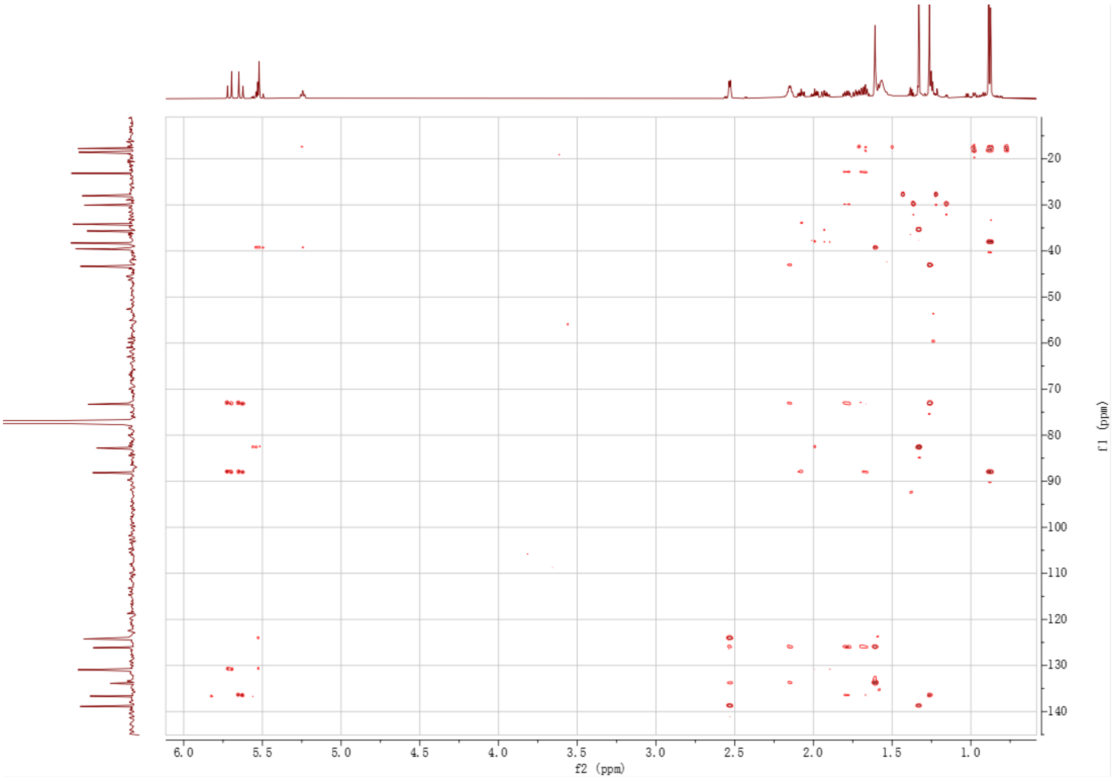

Figure S34. HMBC spectrum (600 MHz) of **4** in $CDCl_3$

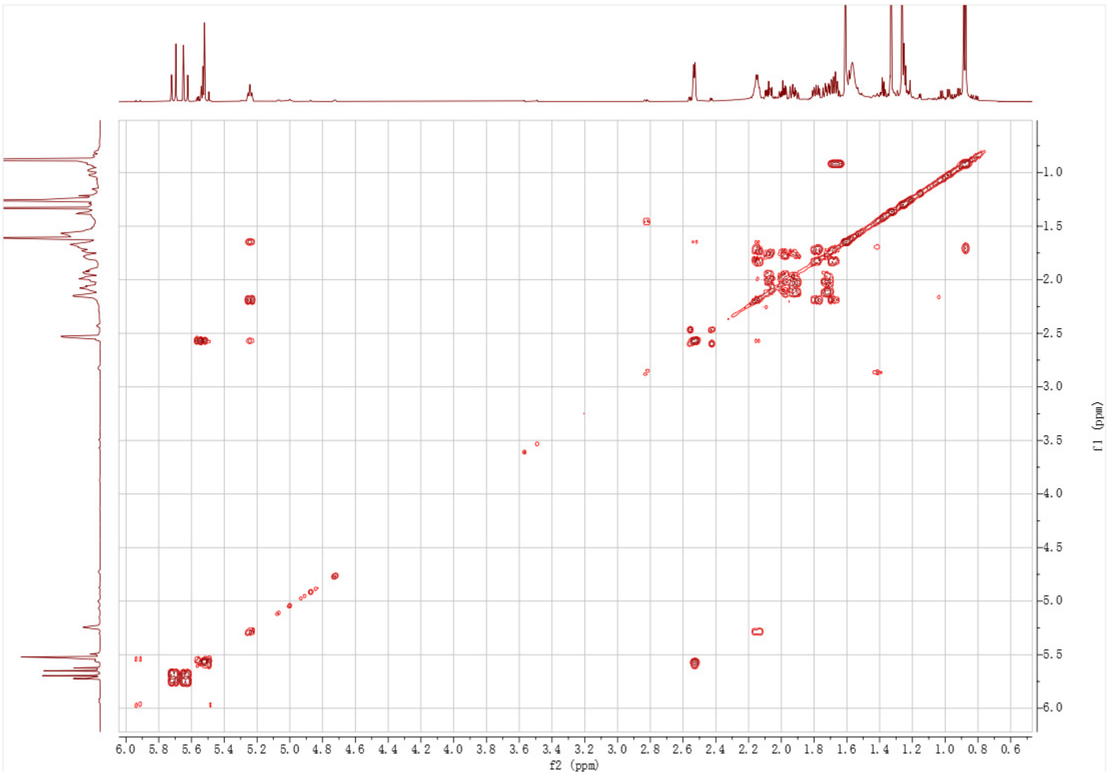

Figure S35. ^{1}H–^{1}H COSY spectrum (600 MHz) of **4** in $CDCl_3$

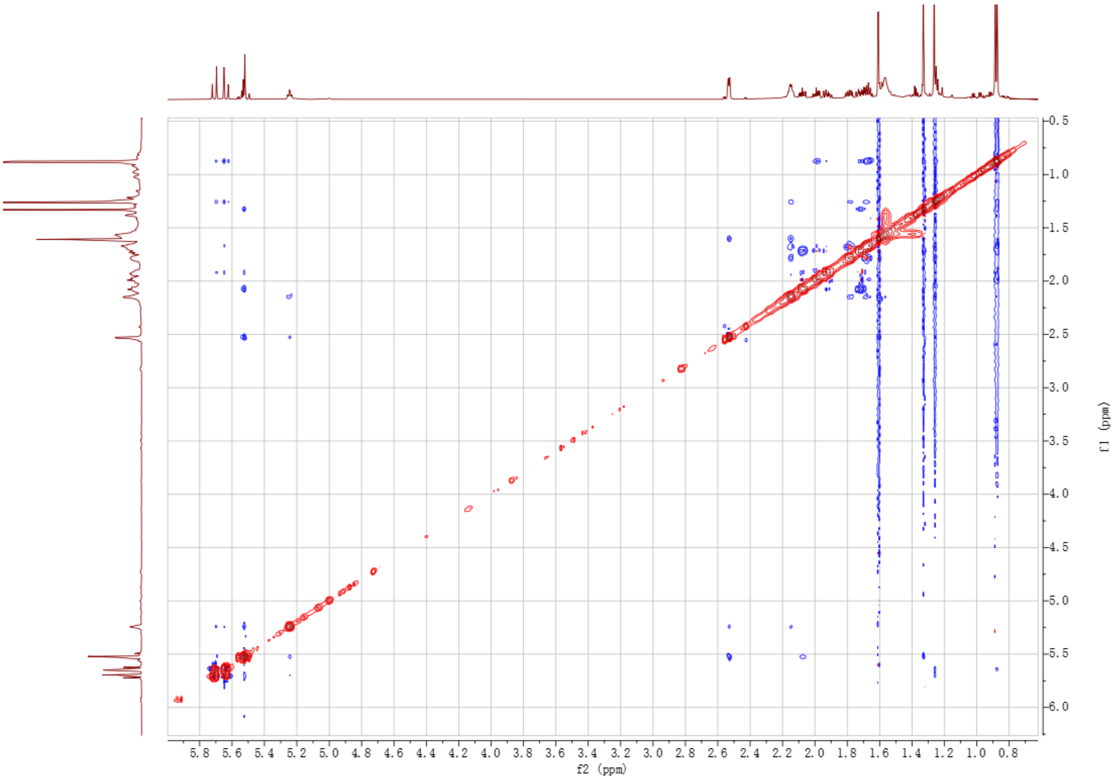

Figure S36. NOESY spectrum (600 MHz) of **4** in $CDCl_3$

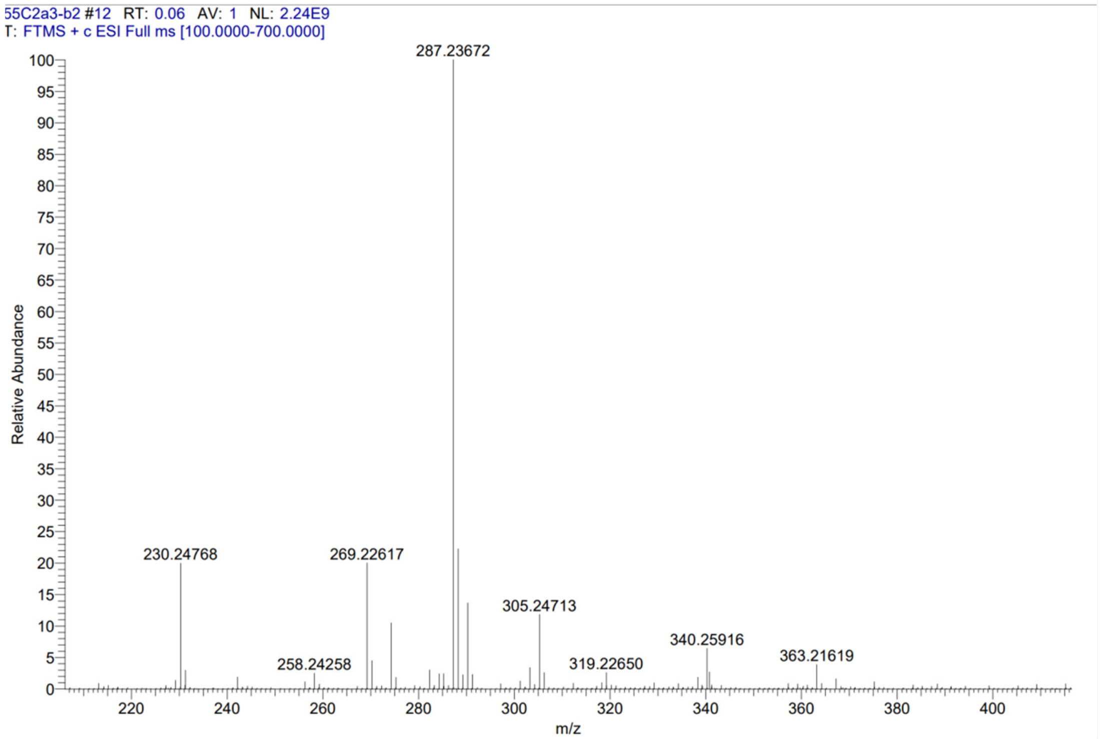

Measured *m/z*	Cal *m/z*	Error (mmu)	Ion Formula	Ion
305.2471	305.2475	-0.4	$C_{20}H_{33}O_2$	$[M+H]^+$
287.2367	287.2369	-0.2	$C_{20}H_{31}O$	$[M-H_2O+H]^+$

Figure S37. HR-ESIMS spectrum of **4**

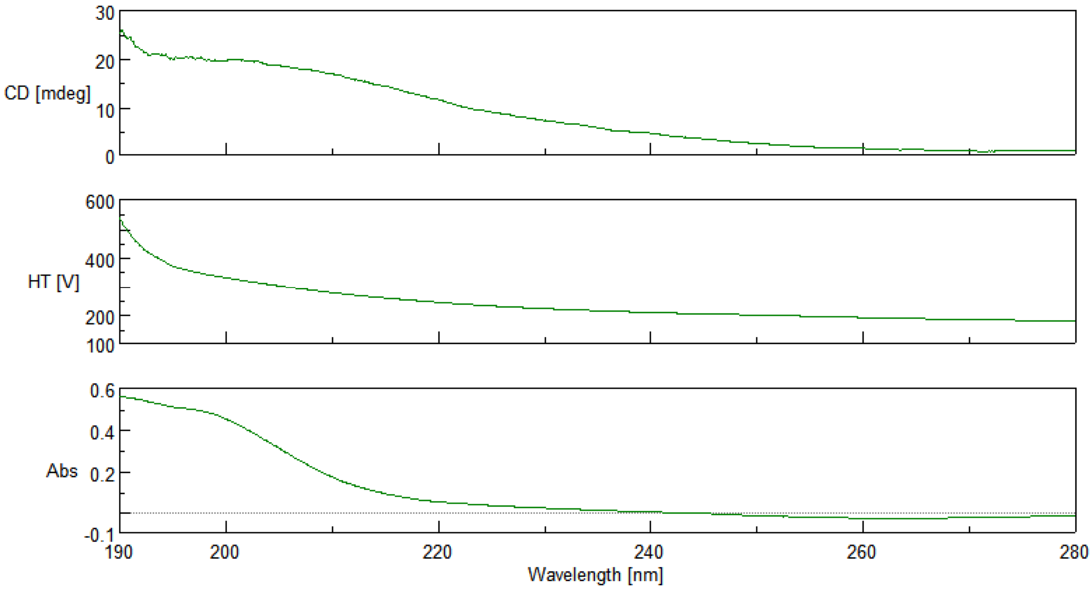

Figure S38. ECD and UV spectra of **4**

5. Original spectra of **5**.

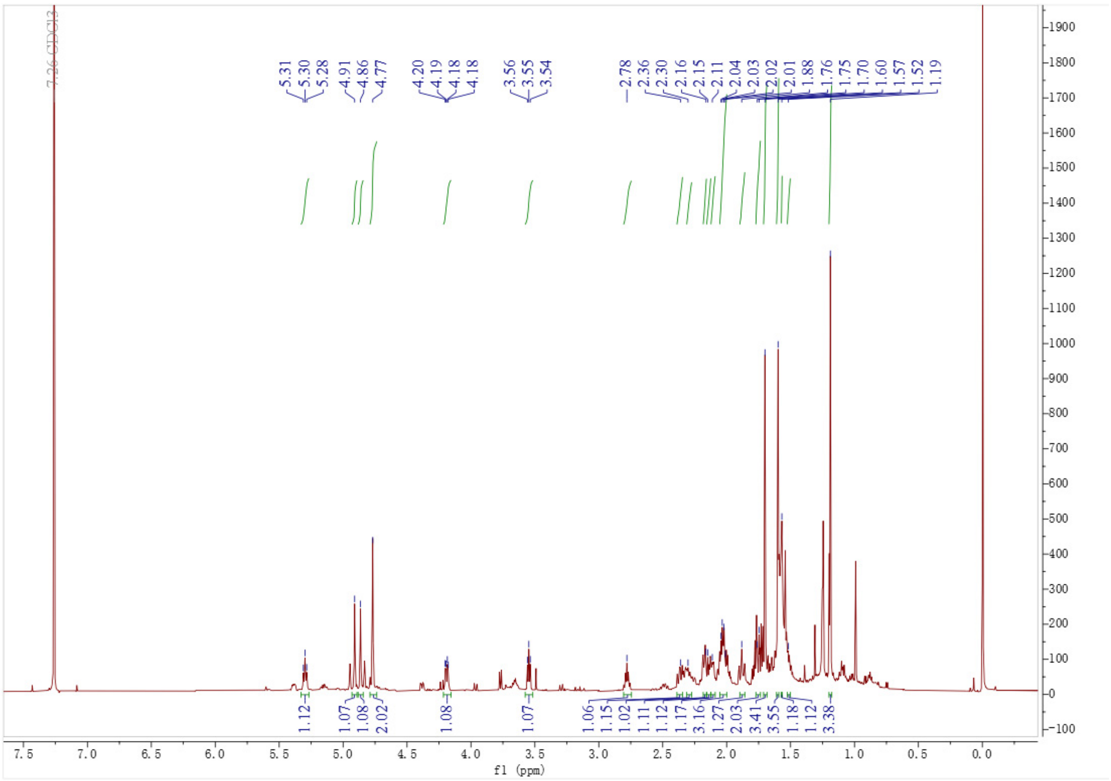

Figure S39. ^{1}H NMR spectrum (600 MHz) of **5** in $CDCl_3$

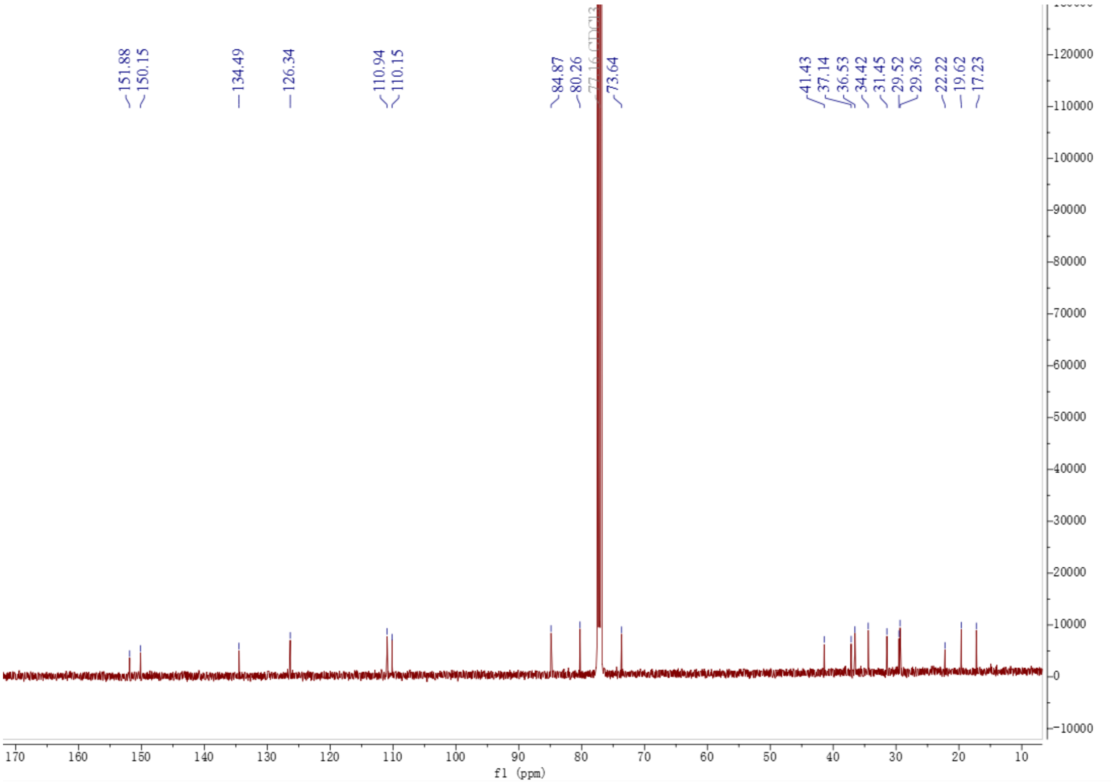

Figure S40. ^{13}C NMR spectrum (150 MHz) of **5** in $CDCl_3$

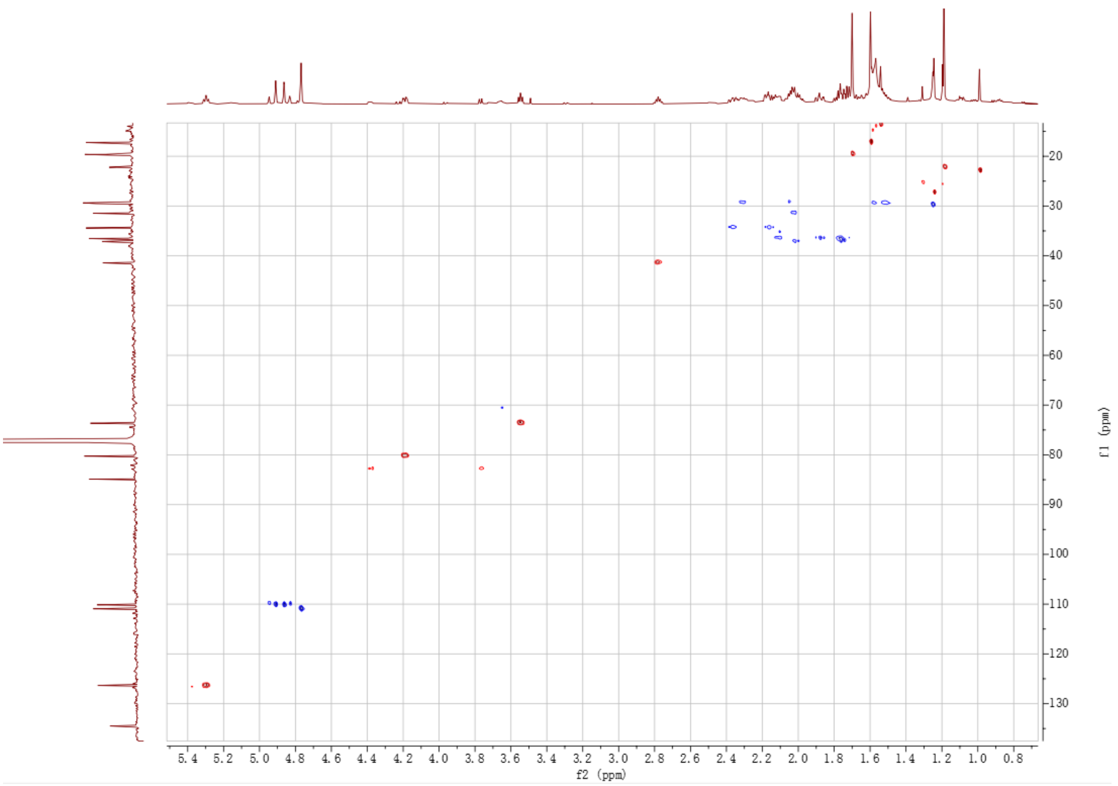

Figure S41. HSQC spectrum (600 MHz) of **5** in $CDCl_3$

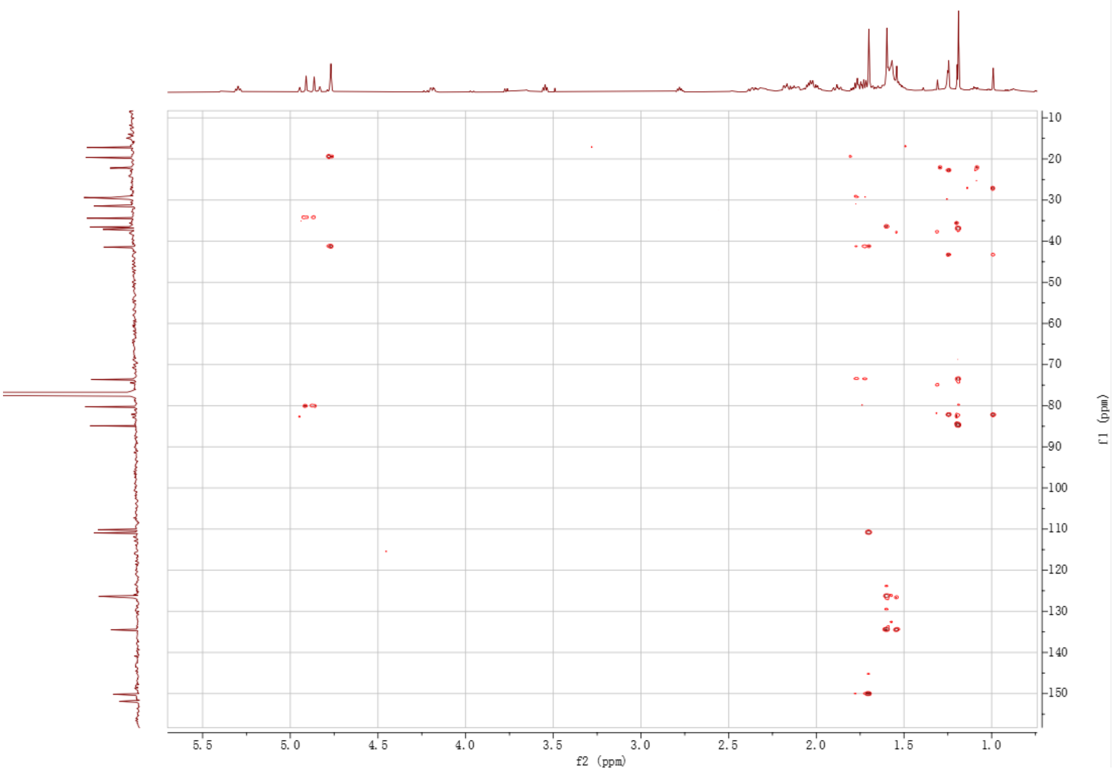

Figure S42. HMBC spectrum (600 MHz) of **5** in $CDCl_3$

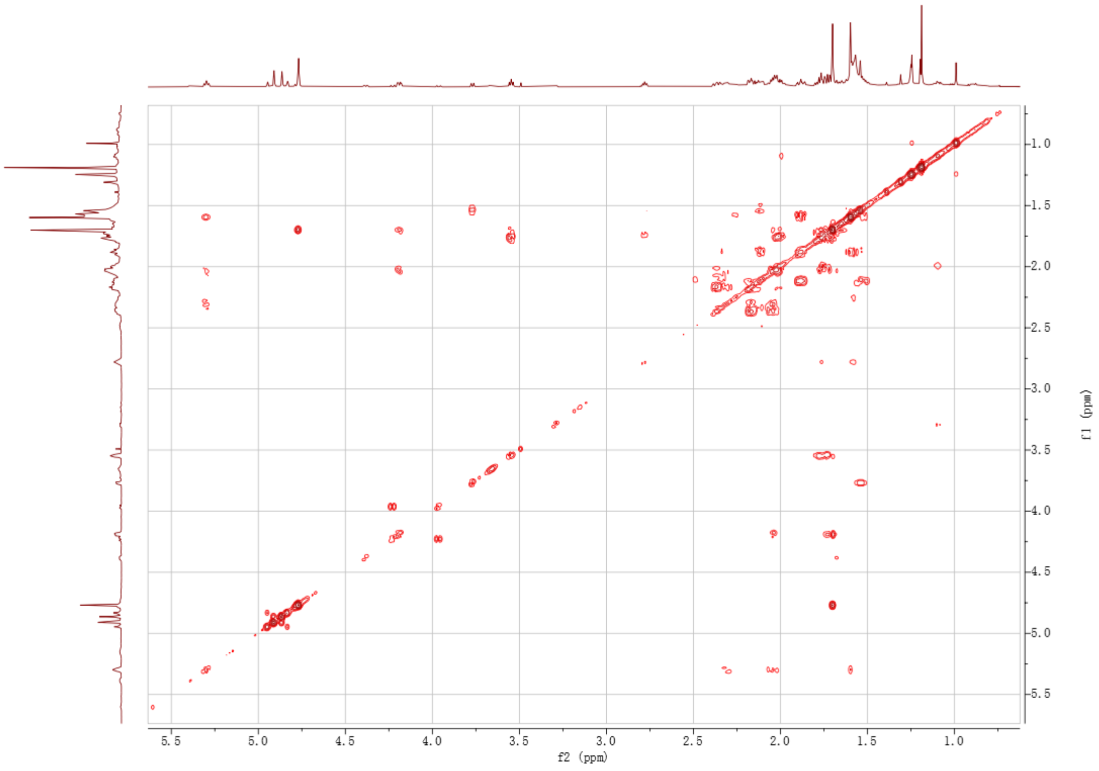

Figure S43. ^{1}H–^{1}H COSY spectrum (600 MHz) of **5** in $CDCl_3$

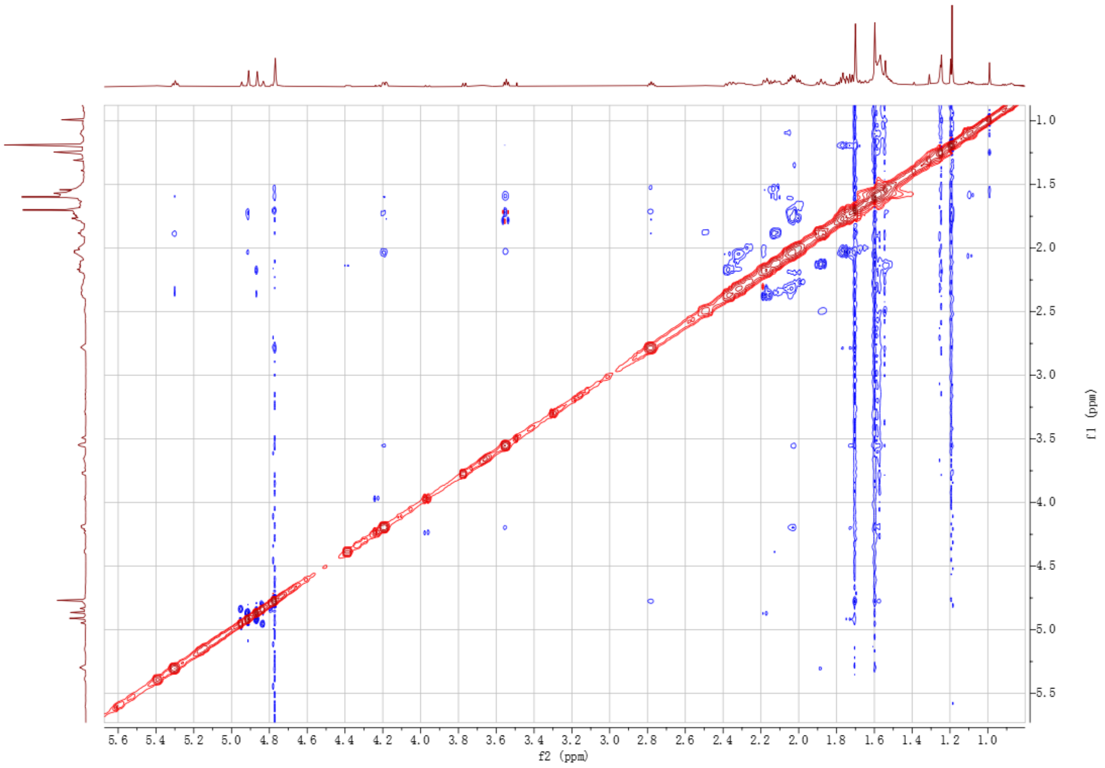

Figure S44. NOESY spectrum (600 MHz) of **5** in $CDCl_3$

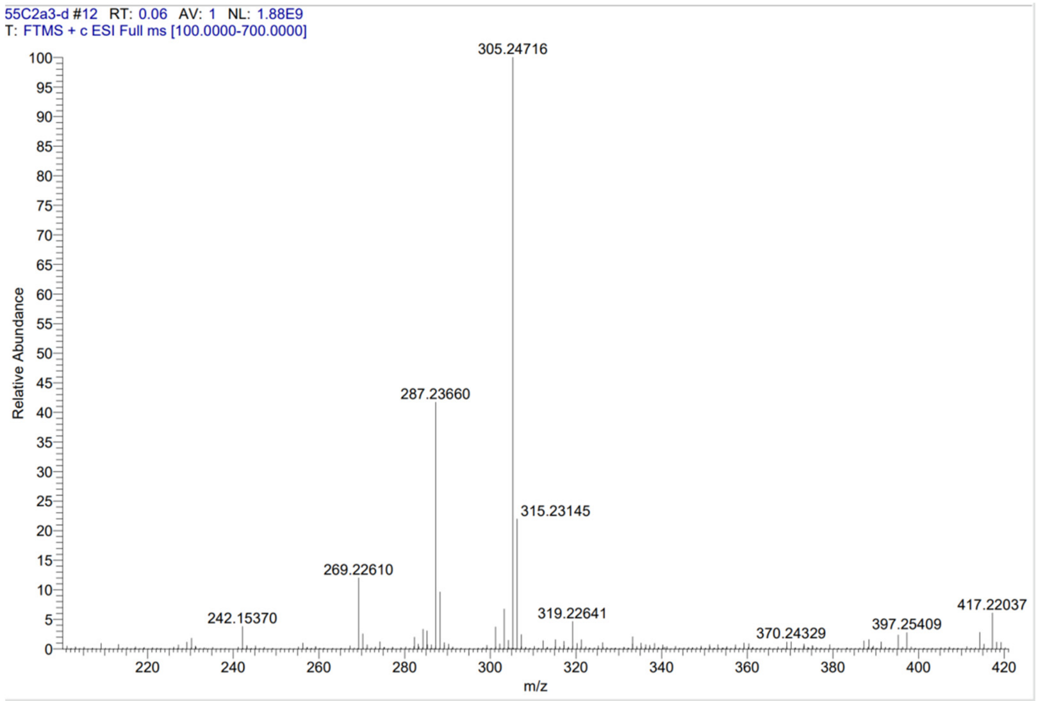

Measured *m/z*	Cal *m/z*	Error (mmu)	Ion Formula	Ion
305.2472	305.2475	-0.3	$C_{20}H_{33}O_2$	$[M+H]^+$

Figure S45. HR-ESIMS spectrum of **5**

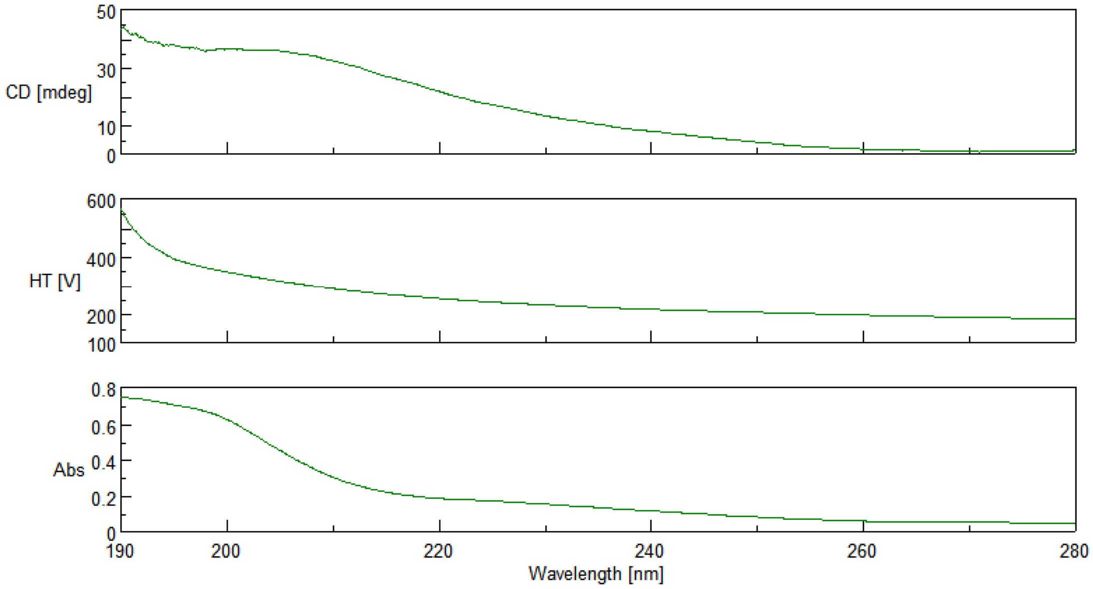

Figure S46. ECD and UV spectra of **5**

6. Original spectra of **7**.

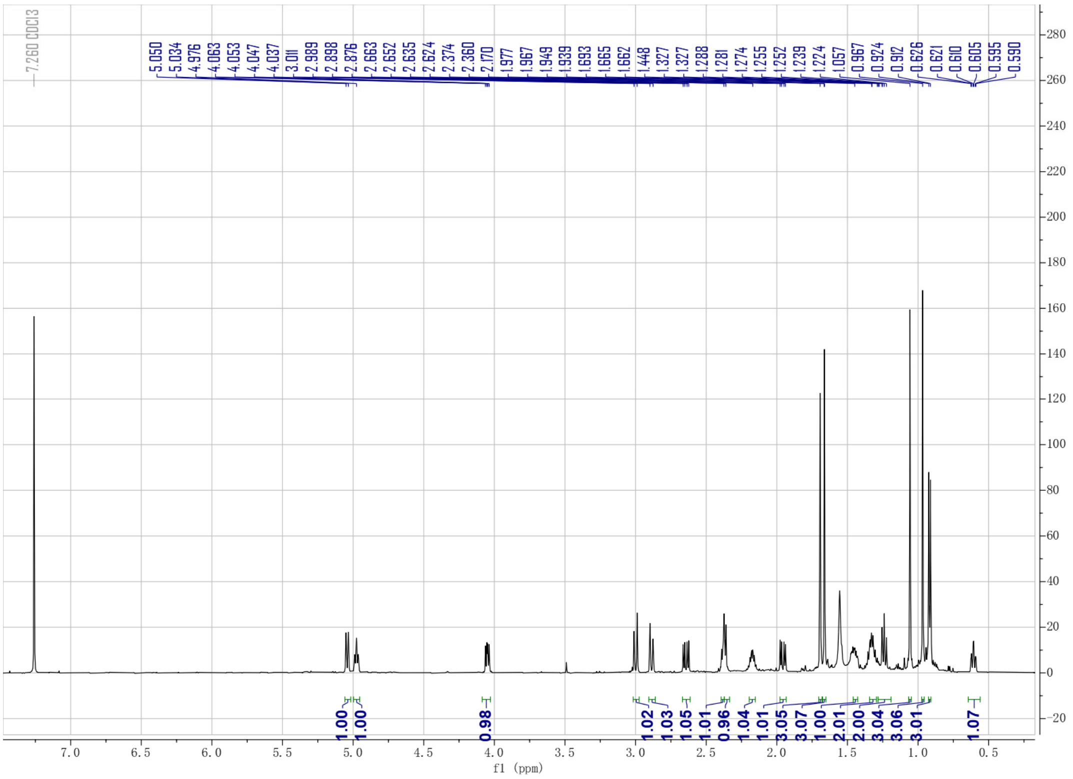

Figure S47. ^{1}H NMR spectrum (600 MHz) of **7** in $CDCl_3$

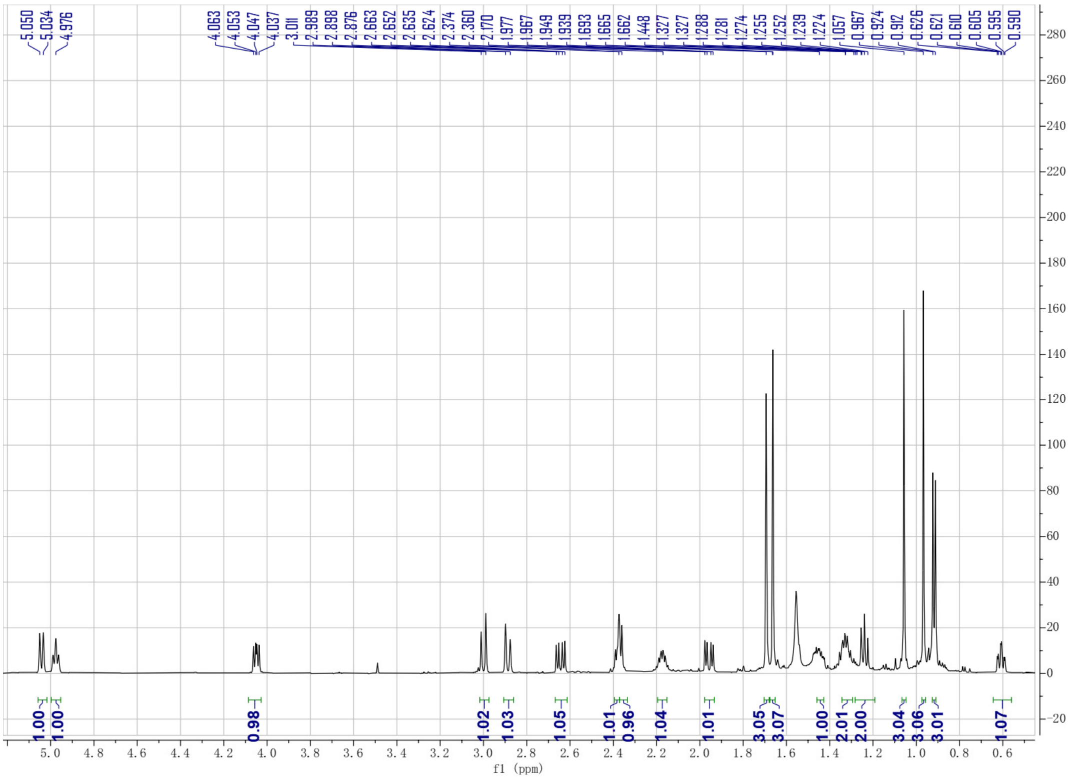

Figure S48. Localized amplified ^{1}H NMR spectrum (600 MHz) of **7** in $CDCl_3$

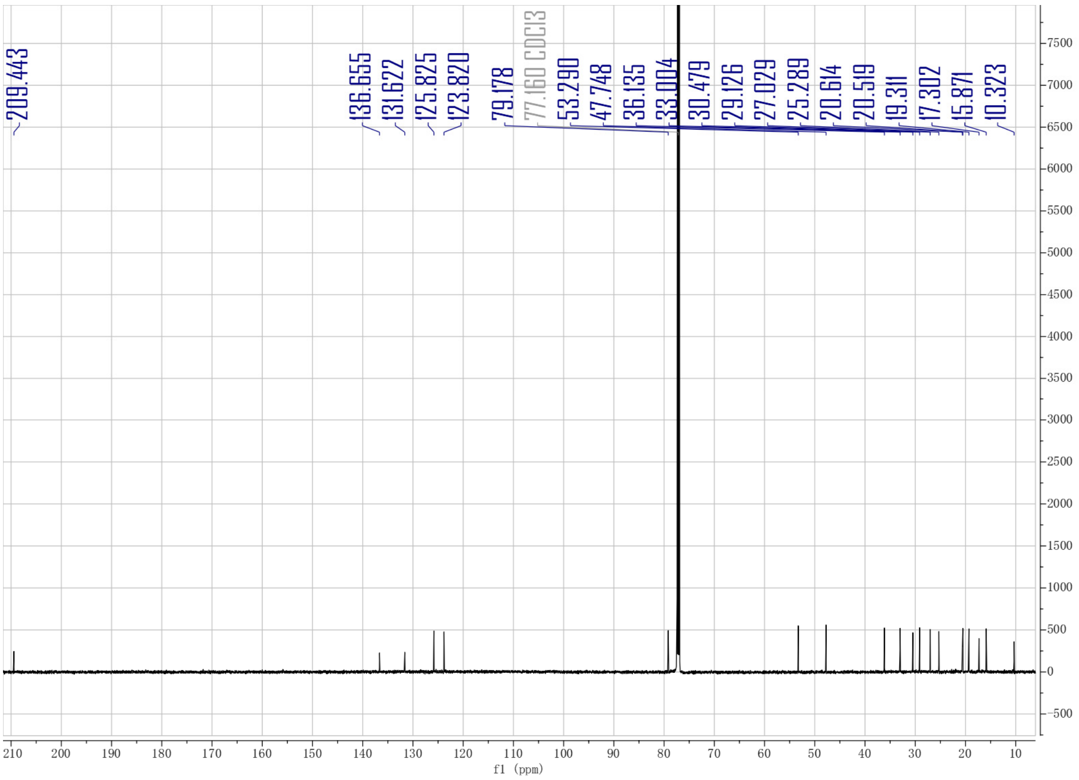

Figure S49. ^{13}C NMR spectrum (150 MHz) of **7** in $CDCl_3$

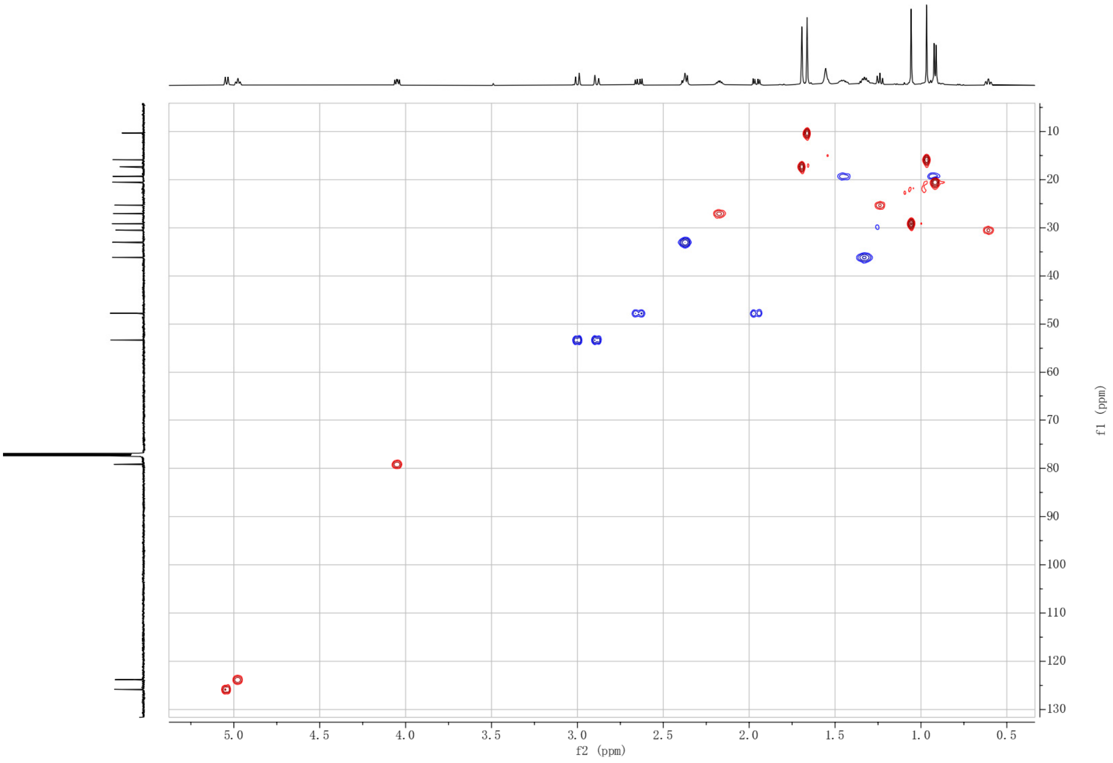

Figure S50. HSQC spectrum (600 MHz) of **7** in $CDCl_3$

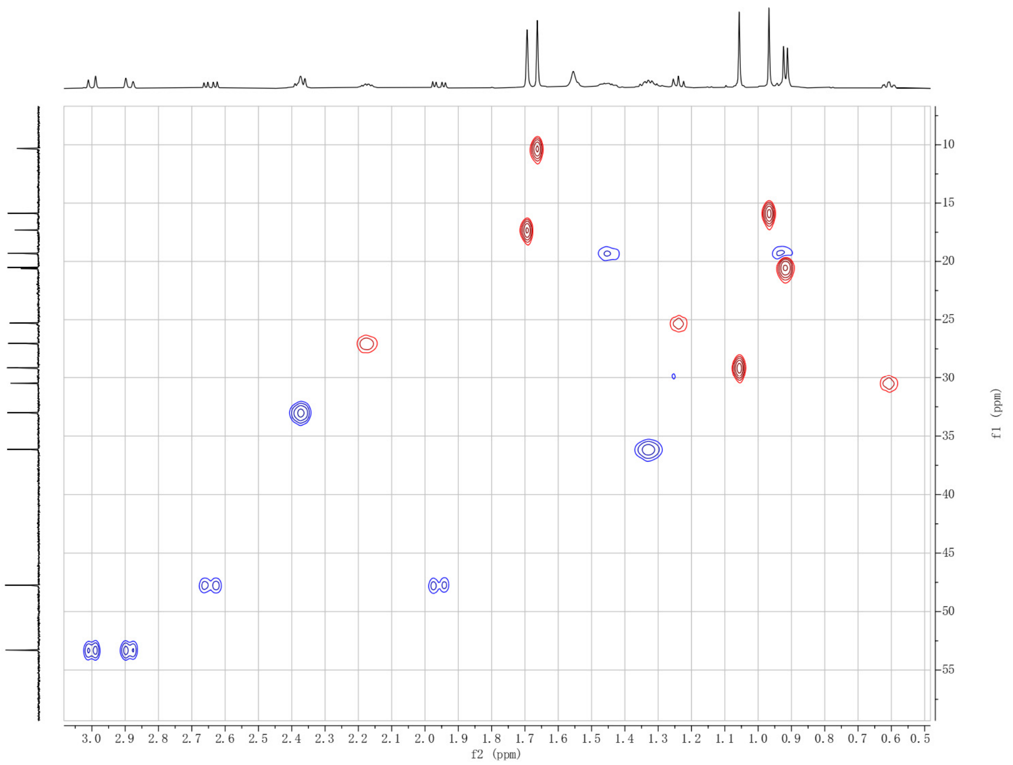

Figure S51. Localized amplified HSQC spectrum (600 MHz) of **7** in $CDCl_3$

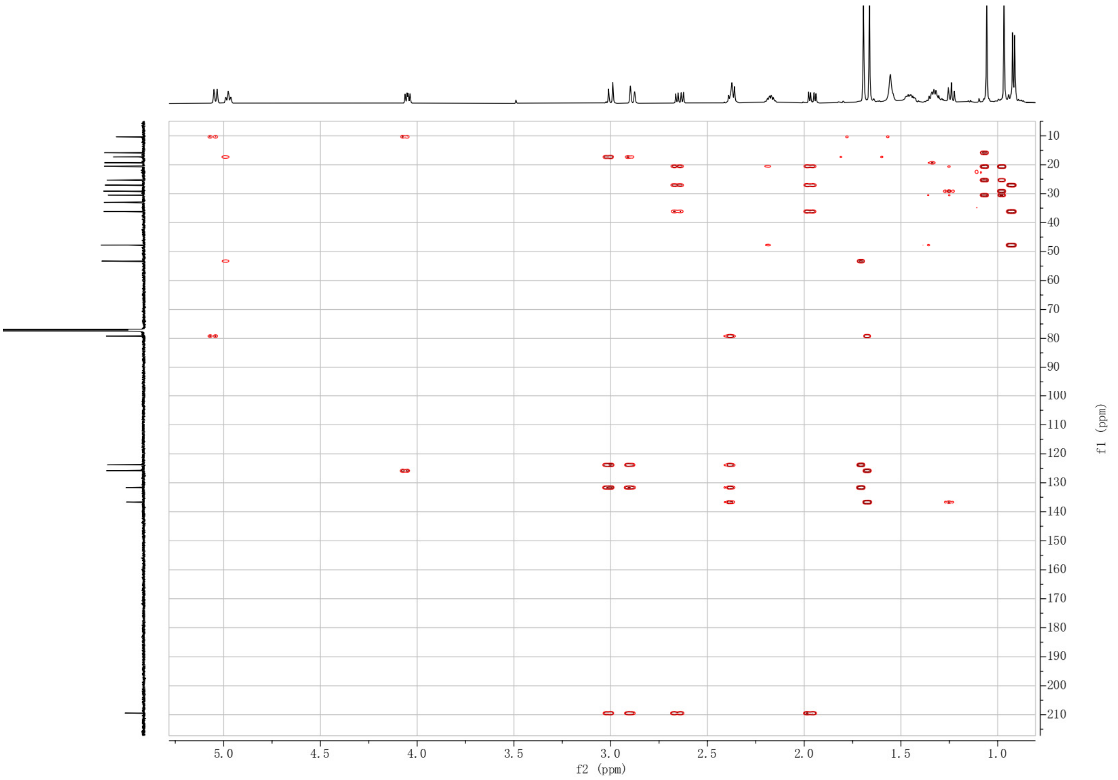

Figure S52. HMBC spectrum (600 MHz) of **7** in $CDCl_3$

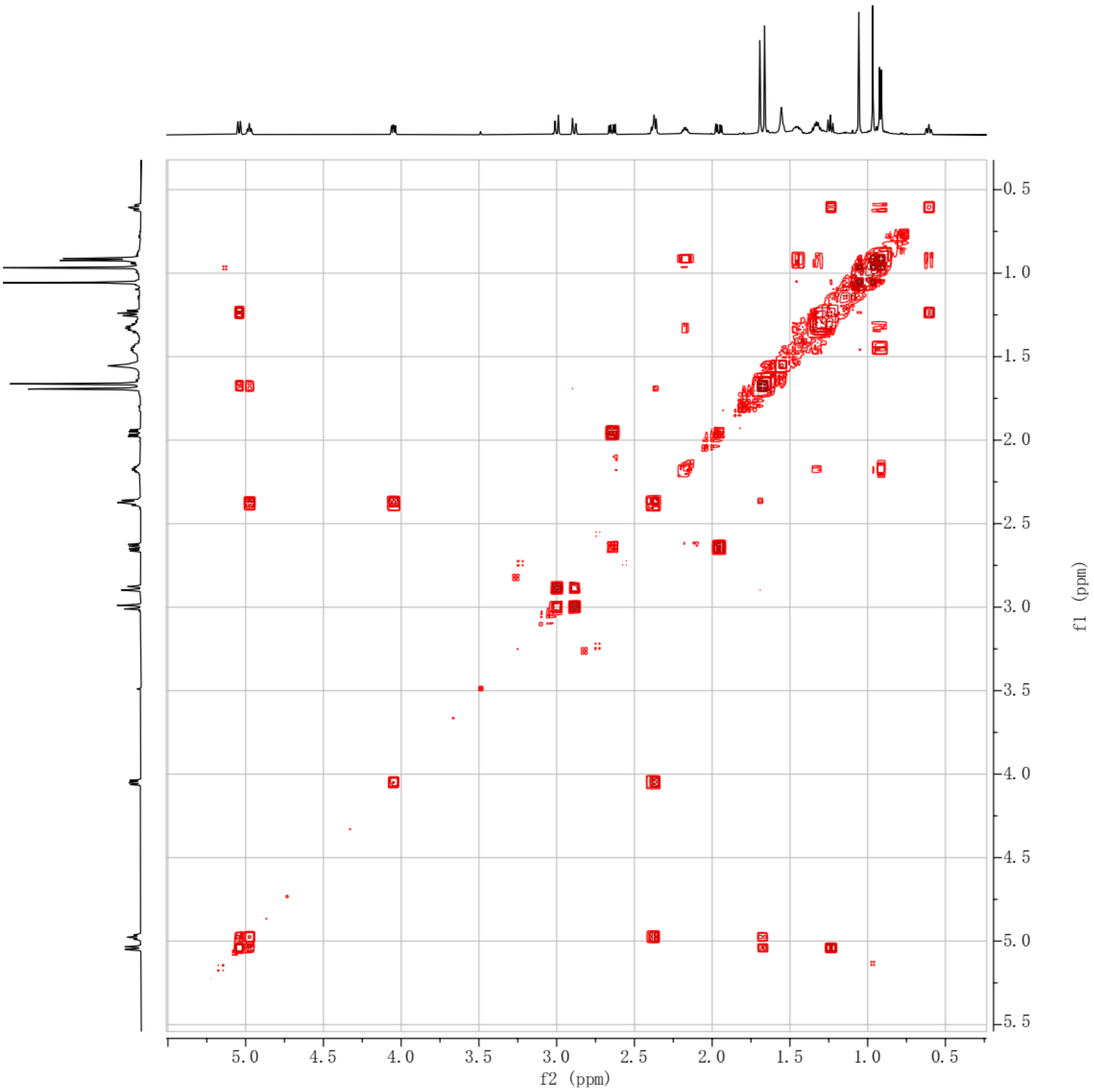

Figure S53. ^{1}H–^{1}H COSY spectrum (600 MHz) of **7** in $CDCl_3$

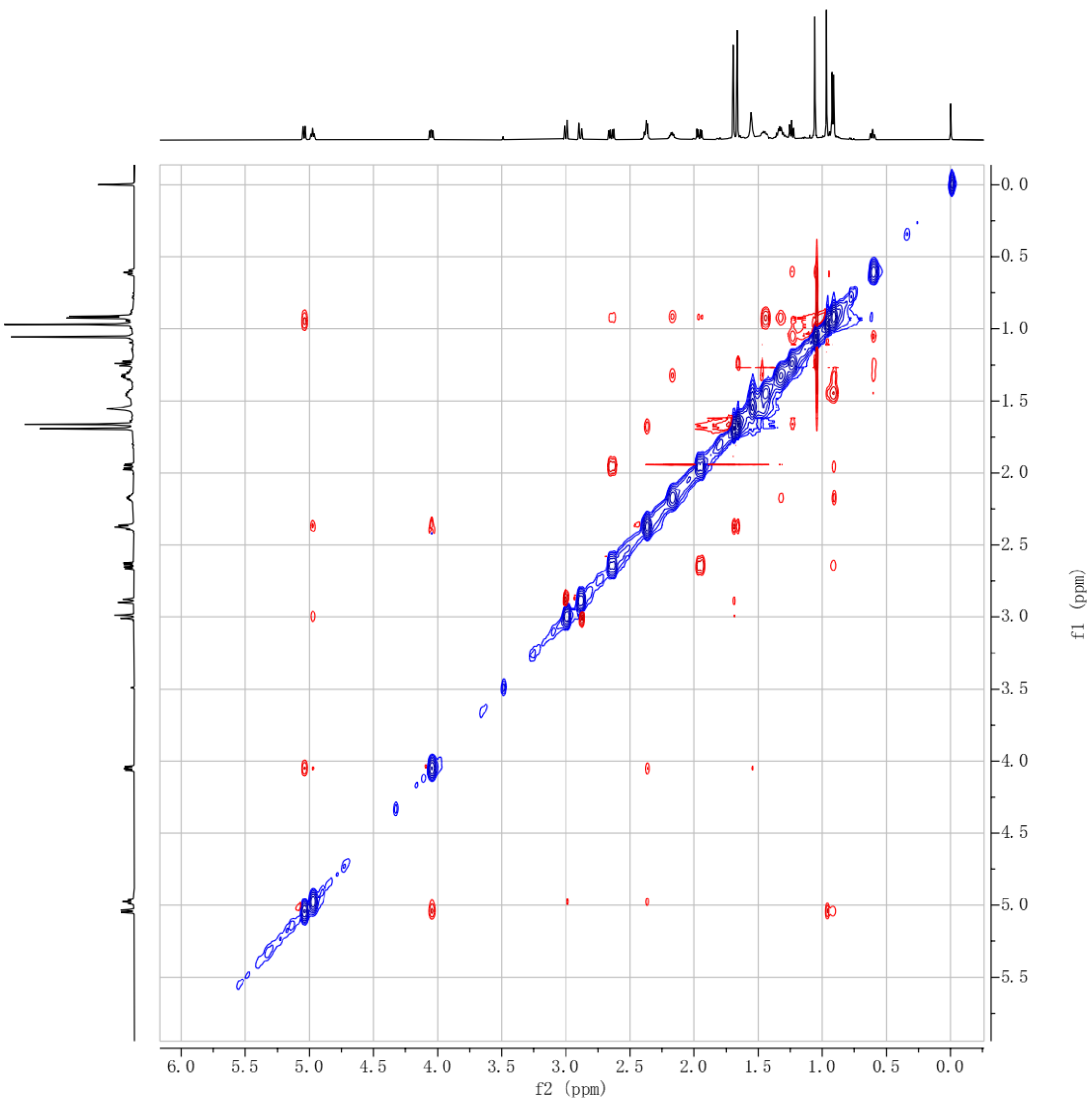

Figure S54. NOESY spectrum (600 MHz) of **7** in $CDCl_3$

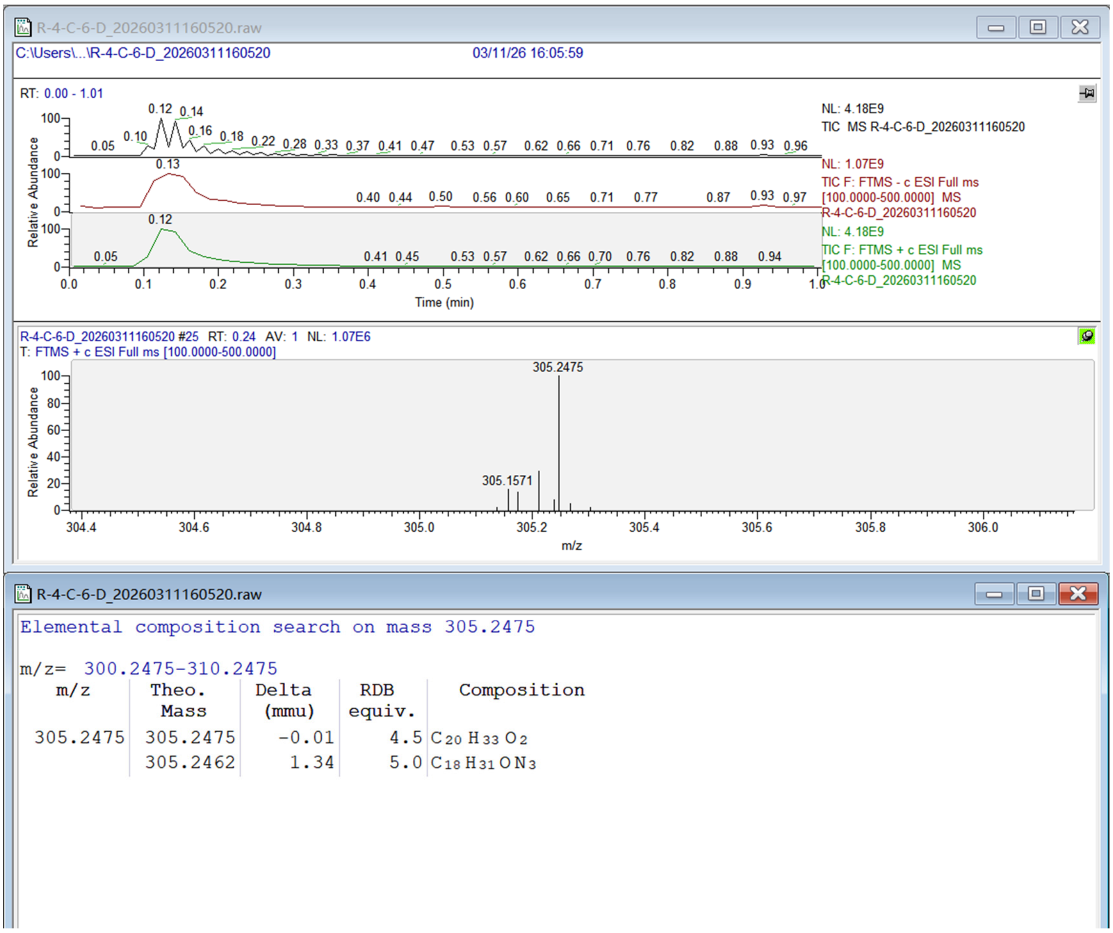

Measured *m/z*	Cal *m/z*	Error (mmu)	Ion Formula	Ion
305.2475	305.2475	-0.01	$C_{20}H_{33}O_2$	$[M+H]^+$

Figure S55. HR-ESIMS spectrum of **7**

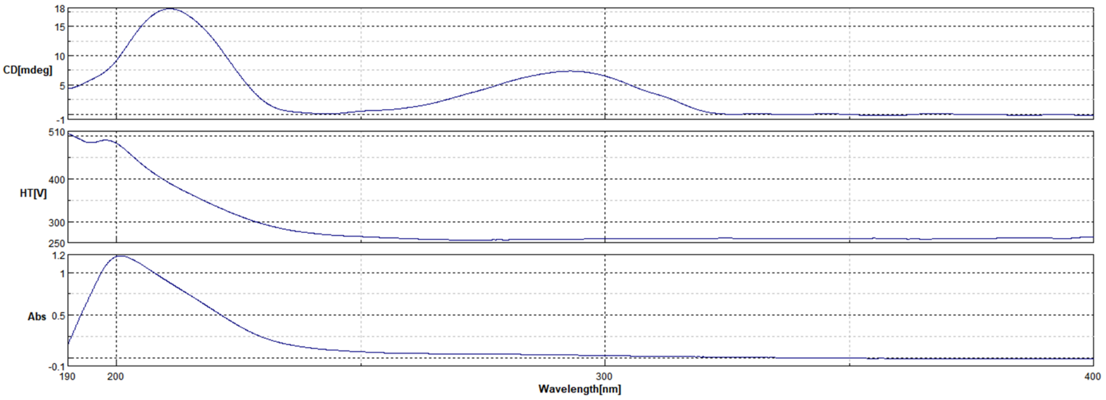

Figure S56. ECD and UV spectra of **7**

7. QM-NMR calculation and DP4+ analysis of **2-5**

Torsional sampling (MCMM) conformational searches using OPLS_2005 force field were carried out by the means of conformational search module in Macro model 9.9.223 software (Schrodinger, http://www.schrodinger.com/MacroModel), applying an energy window of 21 kJ/mol (5.02 kcal/mol) for saving structures. The following DFT calculations were performed using Gaussian 09, and the dominant conformers were re-optimized at B3LYP/6-311G** level of theory. Magnetic shielding constants (σ) were calculated by means of the gauge including atomic orbitals (GIAO) method at mPW1PW91/6-31G* or mPW1PW91/6-311G** level of theory as recommended for DP4+ analysis.

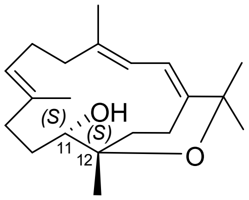

(11*S**,12*S**)-**2**

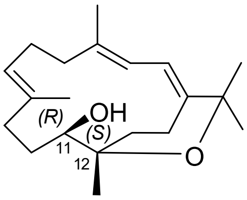

(11*R**,12*S**)-**2**

Figure S57. Structures of studied isomers for compound **2**

Functional		Solvent?		Basis Set		Type of Data
mPW1PW91		PCM		6-31G(d)		Shielding Tensors
	Isomer 1	Isomer 2	Isomer 3	Isomer 4	Isomer 5	Isomer 6
sDP4+ (H data)	40.81%	59.19%	–	–	–	–
sDP4+ (C data)	99.99%	0.01%	–	–	–	–
sDP4+ (all data)	99.98%	0.02%	–	–	–	–
uDP4+ (H data)	8.57%	91.43%	–	–	–	–
uDP4+ (C data)	99.99%	0.01%	–	–	–	–
uDP4+ (all data)	99.93%	0.07%	–	–	–	–
DP4+ (H data)	6.07%	93.93%	–	–	–	–
DP4+ (C data)	100.00%	0.00%	–	–	–	–
DP4+ (all data)	100.00%	0.00%	–	–	–	–

Figure S58. DP4+ results obtained using experimental NMR data of **2** *versus* calculated NMR data of isomers 1 [(11*S**,12*S**)-**2**] and 2 [(11*R**,12*S**)-**2**]

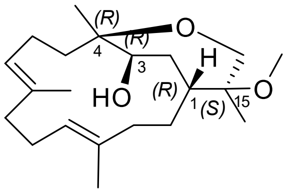

(1*R**,3*R**,4*R**,15*S**)-**3**

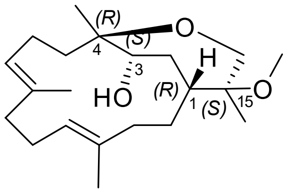

(1*R**,3*S**,4*R**,15*S**)-**3**

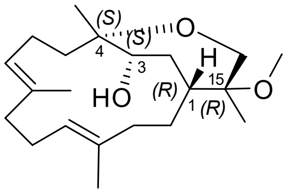

(1*R**,3*S**,4*S**,15*R**)-**3**

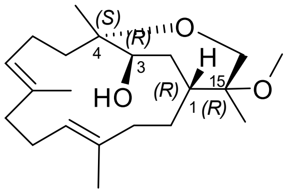

(1*R**,3*R**,4*S**,15*R**)-**3**

Figure S59. Structures of studied isomers for compound **3**

Functional	Solvent?	Basis Set	Type of Data		
mPW1PW91	PCM	6-31G(d)	Shielding Tensors		

	Isomer 1	Isomer 2	Isomer 3	Isomer 4	Isomer 5	Isomer 6
sDP4+ (H data)	0.00%	0.00%	100.00%	0.00%	–	–
sDP4+ (C data)	0.00%	0.00%	100.00%	0.00%	–	–
sDP4+ (all data)	0.00%	0.00%	100.00%	0.00%	–	–
uDP4+ (H data)	0.00%	0.00%	100.00%	0.00%	–	–
uDP4+ (C data)	0.00%	0.00%	100.00%	0.00%	–	–
uDP4+ (all data)	0.00%	0.00%	100.00%	0.00%	–	–
DP4+ (H data)	0.00%	0.00%	100.00%	0.00%	–	–
DP4+ (C data)	0.00%	0.00%	100.00%	0.00%	–	–
DP4+ (all data)	0.00%	0.00%	100.00%	0.00%	–	–

Figure S60. DP4+ results obtained using experimental NMR data of **3** *versus* calculated NMR data of isomers 1 [(1*R**,3*R**,4*R**,15*S**)-**3**], 2 [(1*R**,3*S**,4*R**,15*S**)-**3**], 3 [(1*R**,3*S**,4*S**,15*R**)-**3**] and 4 [(1*R**,3*R**,4*S**,15*R**)-**3**]

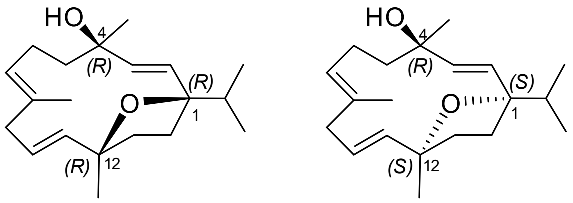

(1*R**,4*R**,12*R**)-**4** (1*S**,4*R**,12*S**)-**4**

Figure S61. Structures of studied isomers for compound **4**

Functional	Solvent?	Basis Set	Type of Data		
mPW1PW91	PCM	6-31G(d)	Shielding Tensors		

	Isomer 1	Isomer 2	Isomer 3	Isomer 4	Isomer 5	Isomer 6
sDP4+ (H data)	21.70%	78.30%	–	–	–	–
sDP4+ (C data)	15.81%	84.19%	–	–	–	–
sDP4+ (all data)	4.94%	95.06%	–	–	–	–
uDP4+ (H data)	0.08%	99.92%	–	–	–	–
uDP4+ (C data)	56.90%	43.10%	–	–	–	–
uDP4+ (all data)	0.11%	99.89%	–	–	–	–
DP4+ (H data)	0.02%	99.98%	–	–	–	–
DP4+ (C data)	19.86%	80.14%	–	–	–	–
DP4+ (all data)	0.01%	99.99%	–	–	–	–

Figure S62. DP4+ results obtained using experimental NMR data of **4** *versus* calculated NMR data of isomers 1 [(1*R**,4*R**,12*R**)-**4**] and 2 [(1*S**,4*R**,12*S**)-**4**]

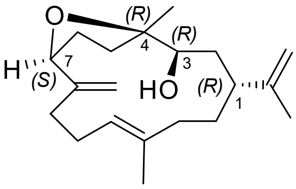

(1*R**,3*R**,4*R**,7*S**)-**5**

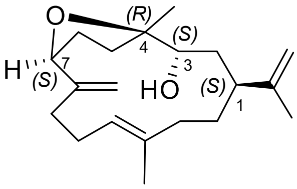

(1*S**,3*S**,4*R**,7*S**)-**5**

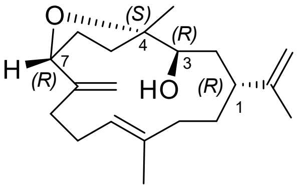

(1*R**,3*R**,4*S**,7*R**)-**5**

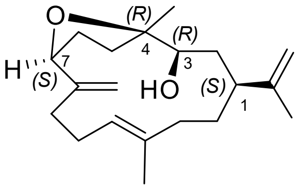

(1*S**,3*R**,4*R**,7*S**)-**5**

Figure S63. Structures of studied isomers for compound **5**

Functional	Solvent?	Basis Set	Type of Data
mPW1PW91	PCM	6-31G(d)	Shielding Tensors

	Isomer 1	Isomer 2	Isomer 3	Isomer 4	Isomer 5	Isomer 6
sDP4+ (H data)	0.00%	90.98%	8.88%	0.14%	-	-
sDP4+ (C data)	0.00%	0.30%	99.57%	0.12%	-	-
sDP4+ (all data)	0.00%	3.03%	96.97%	0.00%	-	-
uDP4+ (H data)	0.00%	1.16%	98.60%	0.23%	-	-
uDP4+ (C data)	0.00%	28.52%	71.46%	0.02%	-	-
uDP4+ (all data)	0.00%	0.47%	99.53%	0.00%	-	-
DP4+ (H data)	0.00%	10.76%	89.23%	0.00%	-	-
DP4+ (C data)	0.00%	0.12%	99.88%	0.00%	-	-
DP4+ (all data)	0.00%	0.01%	99.99%	0.00%	-	-

Figure S64. DP4+ results obtained using experimental NMR data of **5** *versus* calculated NMR data of isomers 1 [(1*R**,3*R**,4*R**,7*S**)-**5**], 2 (1*S**,3*S**,4*R**,7*S**)-**5**], 3 (1*R**,3*R**,4*S**,7*R**)-**5**] and 4 (1*S**,3*R**,4*R**,7*S**)-**5**]

8. TDDFT-ECD calculations of **2-5** and **7**

Conformational searches were carried out using the torsional sampling (MCMM) method OPLS_2005 force field. Conformers above 1% population were re-optimized at the B3LYP/6-311G** level with IEFPCM (Polarizable Continuum Model using the Integral Equation Formalism variant) solvent model for acetonitrile. For the resulting geometries, ECD spectra were obtained by TDDFT calculations performed with the B3LYP/6-311G** level with IEFPCM solvent model for acetonitrile. Finally, the Boltzmann-averaged ECD spectra were obtained with SpecDis 1.71.

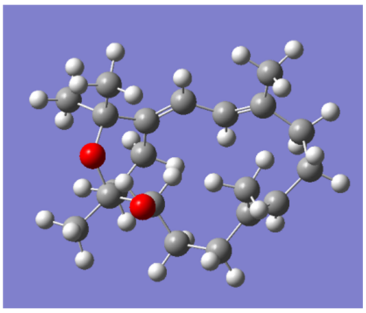

Conf. 1
17.77%
-931.793993 a. u.

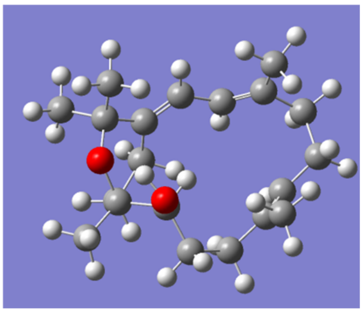

Conf. 2
13.93%
-931.793370 a. u.

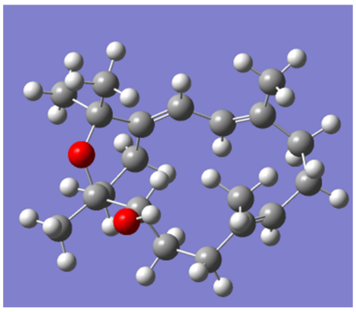

Conf. 3
0.08%
-931.785538 a. u.

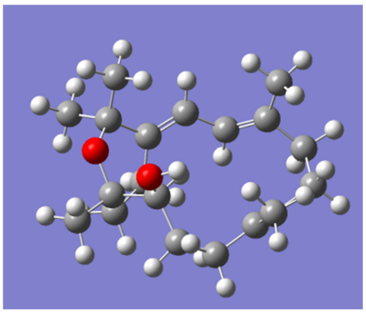

Conf. 4
0.00%
-931.781810 a. u.

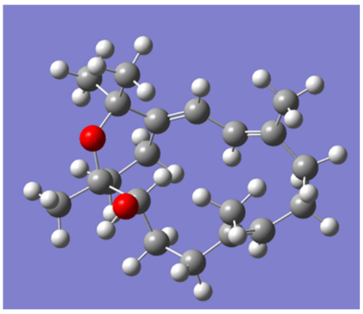

Conf. 5
0.50%
-931.787779 a. u.

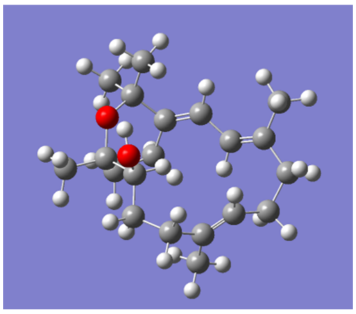

Conf. 6
14.80%
-931.792517 a. u.

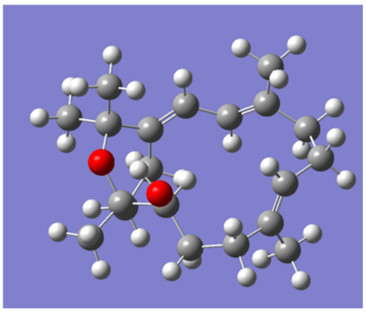

Conf. 7
38.12%
-931.793624 a. u.

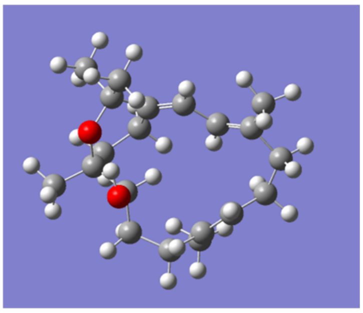

Conf. 8
14.80%
-931.792517 a. u.

Figure S65. Re-optimized conformers of (11*S*,12*S*)-**2** calculated at the B3LYP/6-311G(d,p) level

Table S1. Cartesian coordinates for the re-optimized conformers of (11*S*,12*S*)-**2**

Conf. 1	Coordinates (Angstroms)		
Atom	X	Y	Z
C	-4.07534000	0.14740200	-0.43395200
C	-3.01433400	1.20105300	-0.64420200
C	-3.72834800	-1.27181500	-0.98819200
C	-2.26015400	1.81858600	0.28188300
C	-1.28405000	2.93033900	-0.10163800
C	0.09665600	2.48545300	-0.65142300
C	0.82750500	1.45689100	0.21021500
C	-2.32724000	1.53418500	1.76497200
C	2.22851900	0.98764900	-0.30315800
C	2.21933000	0.40196100	-1.72016700
C	-2.57575700	-1.91804000	-0.25346300
C	-1.33254300	-1.80974500	-0.77280700
C	-0.06880400	-2.02051400	-0.08544200
C	1.34061500	-0.86505400	-1.83402800
C	1.12695500	-1.53372700	-0.48909800
C	-2.90582600	-2.54374200	1.07722800
C	2.33594800	-1.43857100	0.43821200
C	3.56844200	-2.15182900	-0.15459000
C	2.09822000	-1.96388300	1.85698500
O	0.97877700	1.98590700	1.53238300
C	3.26629100	2.10991300	-0.20305700
O	2.68652600	-0.02402300	0.63946600
H	-4.99398100	0.47387800	-0.94325200
H	-4.33567200	0.06239500	0.62591300
H	-2.88014000	1.50508000	-1.68580100
H	-4.63345800	-1.89130200	-0.91400200
H	-3.48901200	-1.18186400	-2.05531900
H	-1.11234500	3.56277200	0.77645200
H	-1.74291600	3.57221600	-0.86453000
H	-0.05184800	2.04683700	-1.64604000
H	0.71521600	3.38265800	-0.78399600
H	0.18901100	0.56968000	0.25898000
H	-1.31570600	1.42427300	2.17101100
H	-2.78070100	2.38461300	2.29508500
H	-2.90512700	0.64063500	2.01237900
H	3.25891500	0.17294600	-1.97942100
H	1.88914600	1.16120700	-2.43703700
H	-1.27684600	-1.36669100	-1.76318800
H	-0.12972100	-2.46675900	0.90223400
H	0.38079500	-0.60386800	-2.28242800
H	1.81245800	-1.57113200	-2.53200400
H	-3.63751900	-3.35279800	0.94315300
H	-2.03490400	-2.96666100	1.58324600
H	-3.36956100	-1.81919900	1.76030600
H	3.39981700	-3.23421000	-0.19548400
H	3.80028700	-1.80817200	-1.16578900
H	4.44247100	-1.95741900	0.47564000
H	1.25412900	-1.45482700	2.33150100
H	1.90788300	-3.04238700	1.86022800
H	2.99154400	-1.77256100	2.45951900
H	1.53097900	1.33004400	1.99441800
H	3.04119500	2.91123700	-0.91529700
H	3.27504500	2.54045400	0.80051300
H	4.26162900	1.71478400	-0.42743800

Conf. 2	Coordinates (Angstroms)		
Atom	X	Y	Z
C	-4.05162000	0.12117300	-0.35327600
C	-2.90004800	0.98652100	-0.79708000
C	-3.84415800	-1.38700900	-0.69913400
C	-2.18245000	1.88044700	-0.09543700
C	-1.06144700	2.65336100	-0.77766100
C	0.26204300	2.78157000	0.01411500
C	0.88418500	1.45865400	0.51141400
C	-2.43058200	2.23337200	1.35212700
C	2.19787900	0.98922000	-0.19401900
C	1.97902400	0.58294800	-1.65468300
C	-2.62032900	-1.96094500	-0.02046300
C	-1.43832300	-1.92196700	-0.67330500
C	-0.11309800	-2.09693100	-0.09345500
C	1.04305600	-0.64210200	-1.80119000
C	1.01182300	-1.50528100	-0.55061700
C	-2.80117400	-2.38724100	1.41340900
C	2.32151900	-1.51709600	0.22667500
C	3.46512900	-2.13624500	-0.60462100
C	2.25849600	-2.22993800	1.58112300
O	1.15430600	1.61831900	1.90804400
C	3.31086500	2.03531600	-0.07077200
O	2.69743600	-0.14393400	0.57364900
H	-4.97411100	0.44736600	-0.85783000
H	-4.23839200	0.22536700	0.72043200
H	-2.62193700	0.84367400	-1.84418900
H	-4.75100100	-1.93567400	-0.41022300

H	-3.74641900	-1.48155900	-1.78818900
H	-1.40906400	3.67996300	-0.97581700
H	-0.86912700	2.20743300	-1.76226200
H	0.96852100	3.34868200	-0.60281800
H	0.09292900	3.39227300	0.90682800
H	0.14352500	0.65911500	0.38818100
H	-2.59186400	3.31536300	1.46153200
H	-3.30490400	1.72738800	1.76829600
H	-1.56274700	1.98318700	1.97516800
H	2.96148900	0.37397900	-2.09259700
H	1.57327600	1.43392400	-2.21420900
H	-1.47481800	-1.59281600	-1.70865300
H	-0.05984300	-2.65170400	0.83804400
H	0.03304700	-0.29670100	-2.02994600
H	1.36460500	-1.23654900	-2.66842900
H	-3.57211300	-3.16669200	1.48633800
H	-1.88700100	-2.77605000	1.86756200
H	-3.14735500	-1.54949800	2.03474500
H	3.28517600	-3.20596300	-0.76188100
H	3.56838200	-1.66494400	-1.58520200
H	4.41335000	-2.01547700	-0.07033300
H	1.47727800	-1.80235900	2.21678700
H	2.07097300	-3.30221400	1.46347500
H	3.21825100	-2.10435400	2.09173500
H	1.70595900	0.84804000	2.13726800
H	3.10180300	2.91833700	-0.68307900
H	3.41430100	2.35405400	0.96964600
H	4.25916100	1.60237700	-0.40281600

Conf. 3	Coordinates (Angstroms)		
Atom	X	Y	Z
C	-4.14763500	0.22881300	-0.38959700
C	-3.07299000	1.26179400	-0.61930800
C	-3.80867300	-1.18917400	-0.95232500
C	-2.23322400	1.81928000	0.27020800
C	-1.20994700	2.86103700	-0.17720300
C	0.13393700	2.31510200	-0.72334300
C	0.94508000	1.47418800	0.27841000
C	-2.24437000	1.50132600	1.74876600
C	2.31469300	0.92883000	-0.25348100
C	2.24444300	0.38952800	-1.69201700
C	-2.64894500	-1.83856900	-0.23066300
C	-1.40633500	-1.71290300	-0.74786900
C	-0.14031400	-1.95991600	-0.07673100
C	1.29493500	-0.82504100	-1.82174500
C	1.06727600	-1.50974300	-0.48768200
C	-2.97602700	-2.49160500	1.08797300
C	2.29894900	-1.50557400	0.41561600
C	3.45528000	-2.31300400	-0.21280500
C	2.05176000	-2.02359100	1.83652500
O	1.22501900	2.23816900	1.46001800
C	3.40493600	2.00021400	-0.14660700
O	2.76624600	-0.13446000	0.61439800
H	-5.06950500	0.56463700	-0.88560200
H	-4.39167400	0.13971000	0.67418400
H	-2.97581700	1.58265000	-1.65959100
H	-4.71361100	-1.80777100	-0.87184400
H	-3.58073200	-1.09447900	-2.02143600
H	-0.99429900	3.54446400	0.65424000
H	-1.65285200	3.47750900	-0.96899800
H	-0.08425000	1.71275700	-1.61245800
H	0.73760700	3.17204400	-1.05139200
H	0.34660400	0.59965400	0.54585700
H	-1.32853200	0.97684700	2.05684400
H	-2.30178500	2.42360700	2.34284100
H	-3.08131800	0.86530800	2.04559500
H	3.26557700	0.10204100	-1.96433400
H	1.95450200	1.18083500	-2.39088400
H	-1.35094600	-1.24658000	-1.72788700
H	-0.20095300	-2.42466000	0.90245800
H	0.34343500	-0.50789400	-2.25262300
H	1.72023700	-1.54149200	-2.53897400
H	-3.69412100	-3.31007700	0.93820100
H	-2.10123700	-2.90851800	1.59211400
H	-3.45526000	-1.78567100	1.78050600
H	3.20214400	-3.37878300	-0.25800200
H	3.69015800	-1.97710700	-1.22632400
H	4.35597900	-2.19208500	0.39792600
H	1.26180000	-1.45141500	2.33125500
H	1.78358100	-3.08579800	1.84247900
H	2.96917600	-1.89726000	2.41908400
H	0.44970300	2.18472800	2.03602200
H	3.14607200	2.88563100	-0.73811100
H	3.53018300	2.30391100	0.89342600
H	4.35304100	1.59763300	-0.51663300

Conf.4	Coordinates (Angstroms)		
Atom	X	Y	Z
C	-4.06126100	0.17170100	-0.38228200
C	-2.86437500	0.97353900	-0.82659100
C	-3.89248000	-1.35508000	-0.66075500
C	-2.14535500	1.88559600	-0.15117800
C	-0.96409800	2.57440800	-0.82205800
C	0.32453100	2.73076000	0.02139200
C	0.98994000	1.44094900	0.58311400
C	-2.47273700	2.35176600	1.24812800
C	2.25000900	0.93582800	-0.19467300
C	1.93411100	0.57150900	-1.65300100
C	-2.67572200	-1.92179600	0.03771400
C	-1.49438100	-1.91302800	-0.61632800
C	-0.16537200	-2.08536300	-0.04227000
C	0.96011700	-0.62747400	-1.76510300
C	0.95350600	-1.49631000	-0.51726500
C	-2.85723700	-2.29603600	1.48584700
C	2.28978000	-1.55742000	0.21121500
C	3.33738800	-2.30082900	-0.64713000
C	2.23081100	-2.20045000	1.60141600
O	1.43460000	1.66247000	1.92856400
C	3.38821800	1.96286900	-0.11878200
O	2.80060300	-0.21888700	0.46378800
H	-4.95743300	0.50932700	-0.92513700
H	-4.27224100	0.32485600	0.68121600
H	-2.54313500	0.75315800	-1.84691600
H	-4.81075900	-1.86848600	-0.34491100
H	-3.80205400	-1.50029000	-1.74476500
H	-1.27111600	3.59500200	-1.10253900
H	-0.74070700	2.06149700	-1.76527400
H	1.04220200	3.31027000	-0.57027800
H	0.10017000	3.36484000	0.88767700
H	0.24879500	0.63283400	0.57981200
H	-2.55982600	3.44670300	1.28106900
H	-3.40853200	1.93484800	1.62639900
H	-1.68186400	2.08454500	1.96243300
H	2.89004600	0.33170000	-2.13182800
H	1.53132900	1.43712500	-2.19136700
H	-1.53090700	-1.62024700	-1.66280900
H	-0.09997000	-2.64158500	0.88777700
H	-0.04962100	-0.25633500	-1.95157800
H	1.22987900	-1.22736800	-2.64628000
H	-3.61714400	-3.08334700	1.58698500
H	-1.93841800	-2.65369600	1.95613000
H	-3.21881700	-1.44088800	2.07456300
H	3.06940000	-3.35813400	-0.75638800
H	3.42636500	-1.86734500	-1.64761600
H	4.31740700	-2.23408400	-0.16300900
H	3.21929800	-2.12087500	2.06350500
H	1.52038300	-1.66945500	2.24105500
H	1.95787700	-3.26020200	1.55342100
H	0.64430300	1.76971800	2.47849700
H	3.13608200	2.89283600	-0.63844500
H	3.61099700	2.19279100	0.92521700
H	4.28403700	1.54078600	-0.58389600

Conf.5	Coordinates (Angstroms)		
Atom	X	Y	Z
C	-4.11091400	0.18459800	-0.38461300
C	-3.03501400	1.21033500	-0.64036400
C	-3.79137600	-1.24098100	-0.93739600
C	-2.24397000	1.83511700	0.24874400
C	-1.23259700	2.88084300	-0.21369900
C	0.11992800	2.33124400	-0.73345700
C	0.90669300	1.46797700	0.26899600
C	-2.29724700	1.62116400	1.74399400
C	2.29303500	0.93342000	-0.25282600
C	2.24202300	0.39143900	-1.69139300
C	-2.62595900	-1.88591100	-0.22206100
C	-1.38749300	-1.75856900	-0.74847300
C	-0.11931300	-1.98486100	-0.07532400
C	1.30126200	-0.83012600	-1.81846800
C	1.08244500	-1.51854000	-0.48390000
C	-2.94085300	-2.52610400	1.10560200
C	2.30858700	-1.49578100	0.42541000
C	3.47848200	-2.29107100	-0.19252600
C	2.06014400	-2.00463000	1.84909500
O	1.05459100	2.13755200	1.52567500
C	3.37108600	2.01733500	-0.13432400
O	2.75931500	-0.11645100	0.61740300
H	-5.03897300	0.52082300	-0.87005900
H	-4.33913600	0.11243900	0.68362600
H	-2.90674300	1.47223800	-1.69422500
H	-4.69819600	-1.85409500	-0.83851900
H	-3.57601500	-1.15924400	-2.01039200
H	-1.03586200	3.57486000	0.61203200

H	-1.66942300	3.47678700	-1.02506100
H	-0.07722000	1.74011400	-1.63455500
H	0.73443200	3.18744300	-1.05457900
H	0.29850800	0.59669400	0.51009700
H	-1.28802800	1.50195800	2.15261800
H	-2.72435100	2.50649000	2.23798000
H	-2.89702900	0.75626400	2.03708800
H	3.26704200	0.10863400	-1.95477800
H	1.95143100	1.17664800	-2.39727500
H	-1.33971900	-1.30182600	-1.73348400
H	-0.17542300	-2.44507500	0.90597000
H	0.34514400	-0.51709800	-2.24246800
H	1.72795000	-1.54010700	-2.54128000
H	-3.66456000	-3.34217300	0.97035500
H	-2.06268300	-2.94225100	1.60426200
H	-3.40864300	-1.81118100	1.79645700
H	3.24034600	-3.36058600	-0.22751200
H	3.71026300	-1.96276900	-1.20946200
H	4.37634700	-2.15206300	0.41868100
H	1.26579400	-1.43251900	2.33600700
H	1.79971900	-3.06868700	1.86122700
H	2.97514900	-1.86890000	2.43352200
H	1.47902800	2.99399900	1.35894300
H	3.09831500	2.92068800	-0.69380100
H	3.52721100	2.27708600	0.91623300
H	4.32037700	1.64520300	-0.53036500

Conf.6	Coordinates (Angstroms)		
Atom	X	Y	Z
C	-3.84195900	0.12778400	-0.47782200
C	-2.90313300	0.97816200	0.35478300
C	-3.90669900	-1.34297900	-0.01014800
C	-2.20478900	2.06096000	-0.02642300
C	-1.27112500	2.77276500	0.93933000
C	0.23075800	2.71523800	0.55468000
C	0.85393400	1.33272400	0.78259100
C	-2.30087400	2.66794500	-1.40837600
C	2.15159300	1.02565700	-0.03281900
C	1.88054600	0.86665300	-1.53346000
C	-2.58841000	-2.09209100	0.08839600
C	-1.48811000	-1.66064000	-0.56575800
C	-0.11057900	-2.03323900	-0.28812400
C	0.95051300	-0.33351300	-1.83820300
C	0.98574200	-1.38606600	-0.74584200
C	-2.61405800	-3.27491400	1.02353300
C	2.34894200	-1.51551300	-0.07654000
C	3.45275100	-1.93507700	-1.06922400
C	2.39018700	-2.46492100	1.12473800
O	1.13750500	1.22229700	2.17753600
C	3.23521800	2.07265500	0.23285300
O	2.70651700	-0.21295400	0.50142600
H	-3.57069200	0.16920400	-1.53917600
H	-4.86567700	0.52877400	-0.42138600
H	-2.78493800	0.64719500	1.38771900
H	-4.36582600	-1.36261600	0.99040400
H	-4.61121200	-1.89469800	-0.65265500
H	-1.38415600	2.35506400	1.94599600
H	-1.56206200	3.83244100	0.99979700
H	0.35304300	3.02611200	-0.48952100
H	0.78225100	3.43934500	1.16524000
H	0.10997000	0.57511800	0.51046000
H	-1.33738400	2.64740800	-1.93566400
H	-3.03575500	2.16708800	-2.04317000
H	-2.59043100	3.72597700	-1.34091300
H	2.84712500	0.74566400	-2.03436900
H	1.44848400	1.78948300	-1.93534900
H	-1.62266400	-0.82241700	-1.23927900
H	0.03025100	-2.78081400	0.48593600
H	-0.07122200	0.02053400	-1.98916700
H	1.24966500	-0.78594000	-2.79454600
H	-3.46102500	-3.93225600	0.78233500
H	-1.70607500	-3.88078500	0.97728500
H	-2.75907700	-2.95459500	2.06541700
H	3.29259200	-2.96851200	-1.39799700
H	3.48199100	-1.30166700	-1.95877700
H	4.42950600	-1.87277300	-0.57830400
H	2.20840900	-3.50193300	0.82330900
H	3.38177000	-2.41110700	1.58456300
H	1.65050800	-2.18210100	1.87928400
H	1.69023900	0.42345000	2.24923900
H	2.94462700	3.04894700	-0.17016300
H	3.39724500	2.17902600	1.30880300
H	4.17564600	1.76708500	-0.23604500

Conf.7	Coordinates (Angstroms)		
Atom	X	Y	Z

C	4.05472300	-0.34831700	-0.23822400
C	2.98547900	0.62783000	-0.68287200
C	3.57320000	-1.53531100	0.66042100
C	2.56225600	1.74599200	-0.06885600
C	1.53960200	2.65862200	-0.72890300
C	0.10633400	2.63642300	-0.13505900
C	-0.67946500	1.39155000	-0.55426200
C	3.08806700	2.21463100	1.26785200
C	-2.08213700	1.19894200	0.11166800
C	-2.00766200	0.88310900	1.60989800
C	2.30286400	-2.18080900	0.15900100
C	1.14477300	-1.89376200	0.79447700
C	-0.20308300	-2.07302400	0.28111100
C	-1.28770900	-0.45796500	1.89938800
C	-1.28859500	-1.38063800	0.69458900
C	2.41625400	-2.98729200	-1.10885300
C	-2.56539800	-1.28997200	-0.13266200
C	-3.81514700	-1.68110700	0.68204600
C	-2.55284500	-2.10459800	-1.42962300
O	-0.85149500	1.45446800	-1.97116400
C	-2.99286900	2.40104700	-0.15075600
O	-2.73097900	0.09683600	-0.59061200
H	4.85502000	0.16852200	0.30529900
H	4.52554400	-0.76333500	-1.13860200
H	2.51770600	0.39097300	-1.63892400
H	4.39495200	-2.26421700	0.72021000
H	3.40603800	-1.15499900	1.67484500
H	1.46100100	2.42045200	-1.79412700
H	1.91230400	3.69150000	-0.66273700
H	0.15133400	2.70098600	0.95976200
H	-0.42697100	3.52703100	-0.48764800
H	-0.07018500	0.51483000	-0.30801100
H	3.54492500	3.21053900	1.18144300
H	2.27632300	2.31188300	2.00184300
H	3.83341700	1.53743300	1.69351700
H	-3.03437300	0.85998300	1.99164800
H	-1.50764800	1.70229300	2.13803500
H	1.24154800	-1.31570800	1.70917100
H	-0.29887500	-2.68618000	-0.60940700
H	-0.26499500	-0.26017000	2.22370200
H	-1.77857900	-0.95369600	2.74873800
H	3.19592600	-3.75406400	-1.00453000
H	1.48675300	-3.49409300	-1.37904500
H	2.71182200	-2.35765700	-1.95988700

H	-3.79188500	-2.74994300	0.92451100
H	-3.89362900	-1.12514700	1.61939800
H	-4.71500300	-1.47631500	0.09285700
H	-1.70864500	-1.82401000	-2.06625000
H	-2.50097000	-3.17969200	-1.22757300
H	-3.47539400	-1.90426800	-1.98314700
H	-1.51642700	0.76904300	-2.16345900
H	-2.64551800	3.28292200	0.39822300
H	-3.00385900	2.64122100	-1.21715300
H	-4.01370800	2.17133200	0.16920900

Conf.8	Coordinates (Angstroms)		
Atom	X	Y	Z
C	-3.84211900	-0.12781500	0.47770500
C	-2.90333500	-0.97831100	-0.35483700
C	-3.90669800	1.34293200	0.01000700
C	-2.20480800	-2.06094500	0.02649900
C	-1.27119000	-2.77281700	-0.93925700
C	0.23071300	-2.71526700	-0.55470900
C	0.85385200	-1.33271000	-0.78259800
C	-2.30059300	-2.66767200	1.40858500
C	2.15154100	-1.02568500	0.03277800
C	1.88055900	-0.86667000	1.53343300
C	-2.58838900	2.09204200	-0.08835700
C	-1.48807000	1.66043200	0.56564200
C	-0.11055100	2.03313200	0.28805900
C	0.95067300	0.33359100	1.83825600
C	0.98581200	1.38606800	0.74582100
C	-2.61408600	3.27513900	-1.02315100
C	2.34899300	1.51552700	0.07650200
C	3.45285200	1.93499900	1.06913900
C	2.39025300	2.46492400	-1.12478700
O	1.13733100	-1.22214900	-2.17752200
C	3.23513700	-2.07271000	-0.23292700
O	2.70651900	0.21290800	-0.50148500
H	-3.57094800	-0.16926500	1.53907800
H	-4.86586500	-0.52871500	0.42116000
H	-2.78534400	-0.64754900	-1.38786400
H	-4.36565700	1.36258600	-0.99062300
H	-4.61128200	1.89471800	0.65237700
H	-1.38429400	-2.35518200	-1.94594200
H	-1.56213800	-3.83249700	-0.99962700
H	0.35308400	-3.02618200	0.48947000

H	0.78216700	-3.43933500	-1.16534800
H	0.10987000	-0.57516300	-0.51036100
H	-2.59012000	-3.72573000	1.34138400
H	-1.33699200	-2.64700800	1.93566500
H	-3.03535600	-2.16672900	2.04344500
H	2.84717500	-0.74577600	2.03429200
H	1.44842200	-1.78945900	1.93533700
H	-1.62257000	0.82201100	1.23892800
H	0.03022500	2.78069600	-0.48602400
H	-0.07107300	-0.02032500	1.98944600
H	1.25005200	0.78606000	2.79450800
H	-3.46103000	3.93241900	-0.78170300
H	-1.70608800	3.88098000	-0.97678300
H	-2.75917800	2.95511800	-2.06511400
H	3.29276500	2.96844300	1.39792900
H	3.48208600	1.30158800	1.95869300
H	4.42958500	1.87264700	0.57818800
H	1.65054400	2.18212300	-1.87930600
H	2.20851600	3.50194100	-0.82335100
H	3.38182100	2.41106300	-1.58463500
H	1.69029100	-0.42344200	-2.24913100
H	2.94455600	-3.04899400	0.17011000
H	3.39711500	-2.17908700	-1.30888600
H	4.17558800	-1.76713800	0.23591900

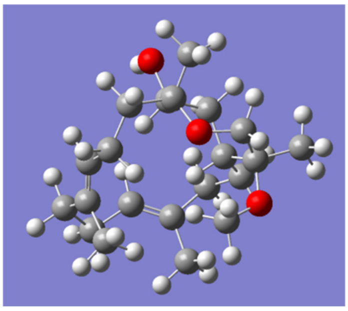

Conf. 1
53.94%
-1047.583302 a. u.

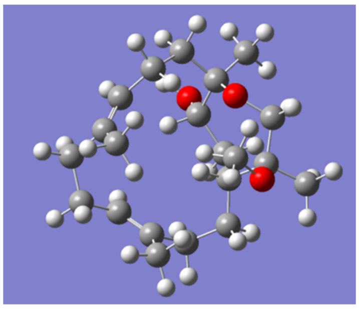

Conf. 2
43.45%
-1047.581529 a. u.

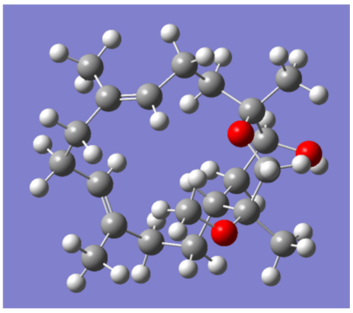

Conf. 3
0.02%
-1047.574371 a. u.

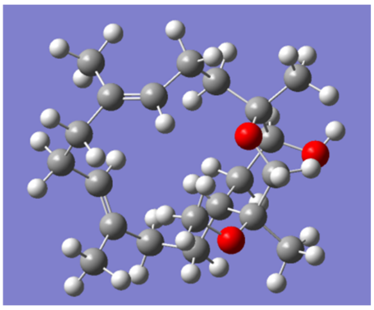

Conf. 4
0.03%
-1047.575094 a. u.

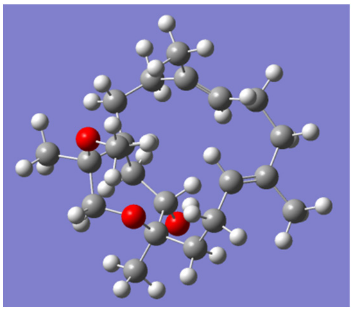

Conf. 5
1.35%
-1047.578070 a. u.

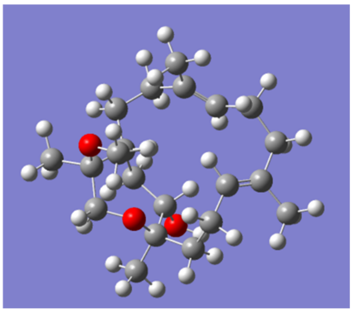

Conf. 6
1.21%
-1047.576910 a. u.

Figure S66. Re-optimized conformers of (1*R*,3*S*,4*S*,15*R*)-**3** calculated at the B3LYP/6-311G(d,p) level

Table S2. Cartesian coordinates for the re-optimized conformers of (1*R*,3*S*,4*S*,15*R*)-**3**

Conf. 1	Coordinates (Angstroms)		
Atom	X	Y	Z
C	-2.9351	0.431	-7.0253
C	-1.8944	-0.6589	-7.096
C	-3.8262	0.5503	-8.2796
C	-0.6603	-0.6443	-6.5588
C	0.2657	-1.8349	-6.7239
C	1.6502	-1.4759	-7.2886
C	1.5816	-0.907	-8.6857
C	-0.1158	0.488	-5.7205
C	2.1483	0.2244	-9.1468
C	2.0224	0.6121	-10.6102
C	-3.2206	1.2585	-9.5246
C	-2.1568	0.3747	-10.1957
C	-1.1319	1.0707	-11.1061
C	1.2734	1.9354	-10.8578
C	-0.1679	1.9732	-10.3069
C	-4.4009	1.547	-10.484
C	2.9553	1.1865	-8.3058
O	-2.8067	-0.6302	-10.9403
C	-0.7237	3.4123	-10.1452
O	-0.015	4.0816	-9.1093
O	-2.6685	2.4609	-9.0094
C	-2.2422	3.4389	-9.9305
C	-0.4373	4.2751	-11.3966
C	-0.3822	3.795	-7.7803
H	-0.0939	1.5264	-9.2917
H	-3.5854	0.1623	-6.1591
H	-2.4913	1.4239	-6.7936
H	-2.2139	-1.5694	-7.6299
H	-4.219	-0.4555	-8.5602
H	-4.7173	1.148	-7.9695

H	-0.1958	-2.6166	-7.3721
H	0.3891	-2.3019	-5.7182
H	2.1818	-0.8156	-6.5704
H	2.2642	-2.4058	-7.3494
H	1.0177	-1.5298	-9.4002
H	0.6835	1.0317	-6.2709
H	-0.8803	1.233	-5.4127
H	0.3201	0.0878	-4.7775
H	1.5424	-0.2057	-11.1938
H	3.054	0.6915	-11.0271
H	-1.575	-0.1701	-9.4221
H	-0.5497	0.2685	-11.6134
H	-1.6295	1.6218	-11.935
H	1.2534	2.1384	-11.9547
H	1.8703	2.7543	-10.3931
H	-5.0589	0.6553	-10.5972
H	-5.0423	2.376	-10.1053
H	-4.0755	1.8011	-11.5176
H	2.3903	2.1338	-8.157
H	3.225	0.8067	-7.2983
H	3.9168	1.4273	-8.8128
H	-2.1972	-1.3287	-11.1169
H	-2.5552	4.4188	-9.5039
H	-2.7956	3.3578	-10.8885
H	-0.8269	3.8048	-12.3277
H	-0.9147	5.2779	-11.3054
H	0.6492	4.4666	-11.5454
H	0.4041	4.2453	-7.1348
H	-1.3534	4.2813	-7.5401
H	-0.4104	2.7017	-7.5918

Conf. 2	Coordinates (Angstroms)		
Atom	X	Y	Z
C	-2.9241	0.3751	-7.1033
C	-1.8689	-0.7007	-7.1719
C	-3.7972	0.498	-8.3696
C	-0.6464	-0.6786	-6.609
C	0.2997	-1.8533	-6.7739
C	1.6906	-1.4655	-7.3026
C	1.6427	-0.8758	-8.6918
C	-0.1352	0.4472	-5.7414
C	2.1983	0.2726	-9.1232
C	2.0966	0.6798	-10.5833
C	-3.1785	1.2239	-9.5979
C	-2.0867	0.3665	-10.2593
C	-1.0544	1.0927	-11.1372
C	1.3306	1.9938	-10.8281
C	-0.1221	2.0005	-10.3069
C	-4.3447	1.5021	-10.5773
C	2.9694	1.236	-8.2508
O	-2.6901	-0.6336	-11.0485
C	-0.706	3.4279	-10.1407
O	-0.0331	4.0971	-9.0811
O	-2.6578	2.43	-9.0595
C	-2.2292	3.4254	-9.9604
C	-0.4071	4.3111	-11.3749
C	-0.4218	3.7853	-7.7639
H	-0.062	1.5423	-9.296
H	-3.5841	0.0881	-6.2504
H	-2.4959	1.3705	-6.8537
H	-2.1657	-1.606	-7.7272
H	-4.1791	-0.5081	-8.6629
H	-4.6979	1.0853	-8.0678
H	-0.1371	-2.6303	-7.4445
H	0.4085	-2.3352	-5.7736
H	2.1972	-0.8088	-6.5633
H	2.3196	-2.3852	-7.3646
H	1.106	-1.4974	-9.4279
H	0.2897	0.0376	-4.7974
H	0.6651	1.0129	-6.2677
H	-0.917	1.1747	-5.4348
H	1.6432	-0.1371	-11.189
H	3.1354	0.7826	-10.9766
H	-1.5002	-0.1829	-9.4922
H	-0.45	0.3053	-11.6422
H	-1.5426	1.6466	-11.9699
H	1.3299	2.2116	-11.9223
H	1.9039	2.8159	-10.3399
H	-4.9826	0.5991	-10.7138
H	-5.0092	2.3142	-10.202
H	-4.0037	1.7749	-11.601
H	2.3847	2.1713	-8.1013
H	3.223	0.8461	-7.243
H	3.9375	1.5007	-8.7325
H	-3.0101	-1.3219	-10.4883
H	-2.5689	4.3948	-9.5301
H	-2.7602	3.3451	-10.9311

H	-0.7675	3.8459	-12.3201
H	-0.9039	5.3043	-11.2823
H	0.679	4.5232	-11.4967
H	0.3389	4.247	-7.0961
H	-1.4098	4.2443	-7.5391
H	-0.4263	2.6894	-7.5887

Conf. 3	Coordinates (Angstroms)		
Atom	X	Y	Z
C	-2.9289	-0.0017	-7.0401
C	-1.5348	0.3357	-6.5687
C	-2.9212	-0.3291	-8.5446
C	-0.618	-0.5236	-6.0858
C	0.7783	-0.0433	-5.7369
C	1.8779	-0.7327	-6.5596
C	1.6699	-0.5505	-8.0437
C	-0.8645	-1.9999	-5.8727
C	2.3145	0.305	-8.8577
C	2.0337	0.3132	-10.3488
C	-3.1683	0.8709	-9.4957
C	-2.6405	0.5196	-10.9015
C	-1.1119	0.5161	-11.0977
C	1.2048	1.5215	-10.8187
C	-0.2638	1.5517	-10.3253
C	-4.6879	1.1533	-9.5272
C	3.3701	1.2822	-8.3932
O	-3.2253	1.3452	-11.882
C	-0.8379	2.9957	-10.3196
O	0.	3.8659	-9.5635
O	-2.533	1.997	-8.9165
C	-2.2739	3.077	-9.7817
C	-0.8141	3.6171	-11.7325
C	0.1774	3.5802	-8.1948
H	-0.2616	1.1956	-9.2705
H	-3.3328	-0.8668	-6.4688
H	-3.6213	0.8366	-6.7974
H	-1.2398	1.3879	-6.7046
H	-1.9514	-0.8239	-8.7837
H	-3.696	-1.1043	-8.7532
H	0.9477	-0.2296	-4.6503
H	0.87	1.0587	-5.8831
H	2.8729	-0.3521	-6.2367
H	1.9181	-1.8243	-6.3374
H	0.9025	-1.2063	-8.4883
H	-0.5873	-2.2854	-4.8329
H	-1.9168	-2.3204	-6.0227
H	-0.2547	-2.6059	-6.5785
H	1.5272	-0.6278	-10.663
H	3.017	0.3034	-10.8756
H	-3.0347	-0.4935	-11.1599
H	-0.7419	-0.4995	-10.8224
H	-0.9018	0.6066	-12.1886
H	1.2135	1.545	-11.9338
H	1.7398	2.4461	-10.5063
H	-5.2513	0.2855	-9.9399
H	-5.0889	1.3591	-8.5094
H	-4.9543	2.0338	-10.1531
H	3.1766	2.3024	-8.7909
H	3.4257	1.3985	-7.2898
H	4.3711	0.9571	-8.7563
H	-3.0883	0.969	-12.7364
H	-2.4185	4.0002	-9.1765
H	-3.0546	3.1569	-10.5666
H	-1.3834	3.0094	-12.4699
H	-1.2615	4.6378	-11.7255
H	0.2218	3.7283	-12.1246
H	0.7683	4.4251	-7.7771
H	-0.7964	3.5355	-7.6613
H	0.7705	2.6508	-8.0588

Conf. 4	Coordinates (Angstroms)		
Atom	X	Y	Z
C	-1.5285	0.2823	-6.6195
C	-2.8626	-0.3817	-8.6317
C	-0.6044	-0.5642	-6.1282
C	0.7744	-0.0601	-5.7446
C	1.9043	-0.7153	-6.5541
C	1.722	-0.5166	-8.0395
C	-0.8247	-2.0478	-5.938
C	2.3629	0.3651	-8.8281
C	2.1121	0.3884	-10.3244
C	-3.1185	0.8257	-9.5713
C	-2.554	0.5026	-10.9701
C	-1.0218	0.5345	-11.1328
C	1.2671	1.5851	-10.795
C	-0.2114	1.578	-10.331

C	-4.643	1.0766	-9.6217
C	3.3859	1.3593	-8.3283
O	-3.1135	1.3291	-11.9651
C	-0.8154	3.0096	-10.3185
O	-0.0138	3.8861	-9.5308
O	-2.5188	1.9573	-8.9656
C	-2.2646	3.0527	-9.8129
C	-0.7723	3.6529	-11.7211
C	0.1386	3.5845	-8.1626
H	-0.2227	1.208	-9.2811
H	-3.3027	-0.9583	-6.5724
H	-3.6196	0.7436	-6.8814
H	-1.2523	1.3418	-6.7365
H	-1.8779	-0.8523	-8.8586
H	-3.6161	-1.1705	-8.8663
H	0.9259	-0.2578	-4.6574
H	0.846	1.0454	-5.8742
H	2.8846	-0.3188	-6.2062
H	1.9628	-1.8088	-6.3459
H	0.9789	-1.1832	-8.5087
H	-0.5639	-2.3404	-4.8959
H	-1.8666	-2.3879	-6.1146
H	-0.1874	-2.6324	-6.6376
H	1.6329	-0.5588	-10.6619
H	3.106	0.4072	-10.8308
H	-2.9058	-0.5189	-11.2565
H	-0.6333	-0.4761	-10.8645
H	-0.7958	0.6456	-12.2189
H	1.2972	1.6238	-11.9092
H	1.7757	2.5165	-10.4595
H	-5.1804	0.2071	-10.0646
H	-5.0658	1.249	-8.6066
H	-4.9174	1.9676	-10.2289
H	3.1762	2.3808	-8.7145
H	3.4173	1.46	-7.2224
H	4.4012	1.0632	-8.6763
H	-3.9799	1.0284	-12.1869
H	-2.4436	3.9656	-9.2011
H	-3.0291	3.1233	-10.6146
H	-1.3101	3.0443	-12.481
H	-1.2426	4.6632	-11.7097
H	0.2699	3.7931	-12.0866
H	0.7014	4.4358	-7.7197
H	-0.8459	3.5116	-7.6524
H	0.7487	2.6663	-8.0259

Conf. 5	Coordinates (Angstroms)		
Atom	X	Y	Z
C	-2.5282	0.8638	-6.7793
C	-1.2111	0.133	-6.8692
C	-3.4849	0.6709	-7.9728
C	-1.0055	-1.174	-6.6248
C	0.3569	-1.8396	-6.7032
C	1.5655	-0.9904	-7.134
C	1.546	-0.6097	-8.5948
C	-2.1111	-2.1262	-6.2243
C	2.1774	0.4382	-9.1562
C	2.0784	0.699	-10.6479
C	-3.0639	1.2892	-9.3355
C	-2.0435	0.3753	-10.039
C	-1.0975	1.0245	-11.0631
C	1.2927	1.977	-10.9991
C	-0.1314	2.033	-10.4069
C	-4.3535	1.4442	-10.1773
C	3.0114	1.4309	-8.3814
O	-2.7363	-0.6717	-10.6806
C	-0.7262	3.4633	-10.3653
O	0.0177	4.2767	-9.4657
O	-2.5235	2.5563	-8.9938
C	-2.2245	3.4514	-10.0407
C	-0.5602	4.1847	-11.7227
C	-0.1998	4.1025	-8.0849
H	-0.0177	1.698	-9.355
H	-3.0398	0.5381	-5.8437
H	-2.329	1.9481	-6.6109
H	-0.358	0.7605	-7.167
H	-3.7146	-0.4115	-8.1093
H	-4.4465	1.1489	-7.6674
H	0.2785	-2.7245	-7.3776
H	0.5685	-2.2358	-5.6818
H	1.6628	-0.1083	-6.4612
H	2.487	-1.6041	-6.9935
H	0.9787	-1.2878	-9.2533
H	-2.189	-2.9513	-6.968
H	-1.8913	-2.572	-5.2279
H	-3.1174	-1.6606	-6.1563
H	1.6236	-0.1749	-11.1672

H	3.113	0.7701	-11.0587
H	-1.4105	-0.1347	-9.2804
H	-0.5107	0.2034	-11.5337
H	-1.6668	1.4784	-11.9046
H	1.2391	2.0777	-12.1088
H	1.8801	2.8484	-10.626
H	-4.9781	0.5225	-10.1342
H	-4.986	2.2825	-9.8041
H	-4.1529	1.6146	-11.2588
H	2.4919	2.4136	-8.3252
H	3.2393	1.1112	-7.3417
H	3.9938	1.5819	-8.8828
H	-2.1306	-1.3667	-10.8823
H	-2.5542	4.4567	-9.6929
H	-2.8408	3.2408	-10.939
H	-0.9966	3.6029	-12.5656
H	-1.0653	5.1779	-11.708
H	0.5068	4.3842	-11.9698
H	0.5476	4.7472	-7.5715
H	-1.215	4.4591	-7.8037
H	-0.0241	3.0518	-7.7713

Conf. 6	Coordinates (Angstroms)		
Atom	X	Y	Z
C	-2.5271	0.8097	-10.4061
C	-1.2012	0.0937	-10.2753
C	-3.4622	0.6171	-8.3145
C	-0.9855	-1.2137	-10.7994
C	0.3855	-1.8631	-10.3632
C	1.5934	-0.9922	-9.4358
C	1.6015	-0.5845	-9.0458
C	-2.0868	-2.1831	-10.0766
C	2.2226	0.487	-11.7074
C	2.1541	0.7708	-8.0628
C	-3.0264	1.2518	-9.356
C	-1.976	0.365	-5.9257
C	-1.017	1.0457	-6.6686
C	1.3537	2.0398	-7.2017
C	-0.0845	2.0624	-8.2067
C	-4.3026	1.3914	-7.7667
C	3.0157	1.4841	-7.4499
O	-2.6242	-0.6778	-5.7393
C	-0.7068	3.481	-6.453
O	-0.0015	4.2976	-6.9954
O	-2.518	2.5255	-9.2823
C	-2.2122	3.4367	-5.3303
C	-0.5213	4.222	-6.2646
C	-0.2479	4.1012	-7.0801
H	0.0105	1.7164	-11.1595
H	-3.0496	0.4694	-10.9992
H	-2.3425	1.8944	-9.3522
H	-0.3501	0.7339	-11.5636
H	-3.6799	-0.4662	-11.9467
H	-4.4334	1.0829	-12.0745
H	0.3307	-2.7354	-10.5661
H	0.5806	-2.2769	-10.2542
H	1.6653	-0.1223	-9.9053
H	2.5188	-1.5979	-11.3507
H	1.0662	-1.2631	-8.2558
H	-1.8778	-2.6405	-7.2746
H	-3.0988	-1.7287	-8.7887
H	-2.1444	-2.9983	-10.1838
H	1.7284	-0.1025	-9.727
H	3.1968	0.8675	-10.9914
H	-1.3413	-0.1504	-12.5678
H	-0.4066	0.2395	-11.6936
H	-1.5729	1.5009	-11.9252
H	1.3241	2.1558	-7.5238
H	1.9164	2.9159	-7.8019
H	-4.909	0.457	-7.7579
H	-4.9584	2.213	-10.4061
H	-4.0847	1.5786	-10.2753
H	2.4742	2.4546	-8.3145
H	3.2245	1.1524	-10.7994
H	4.0067	1.6638	-10.3632
H	-2.911	-1.3326	-9.4358
H	-2.57	4.4317	-9.0458
H	-2.8026	3.2226	-10.0766
H	-0.9253	3.6426	-11.7074
H	-1.0457	5.2052	-8.0628
H	0.5475	4.4449	-9.356
H	0.4724	4.7557	-5.9257
H	-1.2773	4.4318	-6.6686
H	-0.0562	3.0508	-7.2017

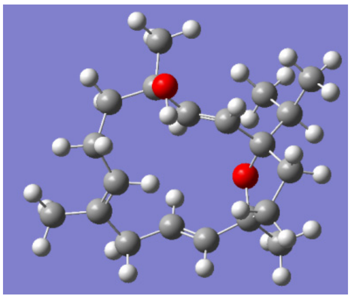

Conf. 1
-931.581021 a. u.

Figure S67. Re-optimized conformers of (1*S*,4*R*,12*S*)-**4** calculated at the B3LYP/6-311G(d,p) level

Table S3. Cartesian coordinates for the re-optimized conformers of (1*S*,4*R*,12*S*)-**4**

Conf. 1	Coordinates (Angstroms)		
Atom	X	Y	Z
C	-3.25325	-1.10173	-0.67472
C	-2.73154	0.28144	-0.40501
C	-3.31378	1.30349	0.24174
C	-2.51429	2.58367	0.51684
C	-1.03824	2.31135	0.59429
C	-0.12457	2.68686	-0.30037
C	-2.53388	-2.19534	0.15355
C	-1.04835	-2.50524	-0.1845
C	-0.09497	-1.36722	0.16986
C	1.31737	2.22308	-0.31535
C	1.72113	1.72399	-1.7261
C	2.68028	0.57156	-1.42565
C	1.03714	-1.10786	-0.49281
C	2.08103	-0.05135	-0.14214
C	3.18839	-0.62471	0.79537
C	2.65798	-0.99874	2.18529
C	3.94293	-1.80403	0.16143
C	-4.72961	1.31621	0.75995
C	-0.62834	-3.7655	0.59397
O	-0.9235	-2.87475	-1.56653
O	1.4931	1.07423	0.5507
C	2.25013	3.3364	0.19178
H	-3.14293	-1.34598	-1.74225
H	-4.32553	-1.17549	-0.46256
H	-1.7063	0.43879	-0.72772
H	-2.73375	3.34208	-0.24973
H	-2.8673	3.00842	1.46902
H	-0.72427	1.64721	1.39692
H	-0.40416	3.34773	-1.1234

H	-2.60533	-1.93718	1.2186
H	-3.08344	-3.13482	0.01488
H	-0.35645	-0.77184	1.04132
H	2.17128	2.51672	-2.33248
H	0.83764	1.3546	-2.2588
H	2.75822	-0.14306	-2.24999
H	3.68802	0.9504	-1.21787
H	1.28294	-1.73885	-1.34707
H	3.89829	0.20458	0.9267
H	1.96504	-1.84588	2.13474
H	3.48953	-1.28701	2.83948
H	2.1372	-0.15411	2.64462
H	3.28655	-2.67187	0.02828
H	4.77041	-2.11441	0.80976
H	4.36783	-1.54841	-0.81614
H	-5.29766	0.42622	0.47712
H	-4.74711	1.39303	1.85636
H	-5.27259	2.19331	0.38039
H	-0.69027	-3.59705	1.67433
H	0.40162	-4.03205	0.34038
H	-1.28244	-4.60456	0.33244
H	-1.00559	-2.05943	-2.08683
H	2.08643	4.26824	-0.36236
H	2.05157	3.52736	1.25073
H	3.30243	3.04954	0.08322

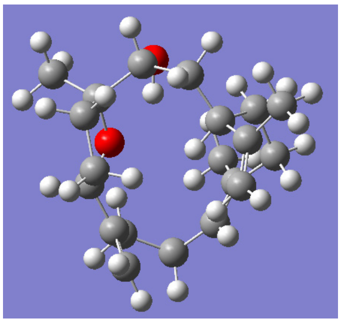

Conf. 1

-931.570618 a. u.

Figure S68. Re-optimized conformers of (1*R*,3*R*,4*S*,7*R*)-**5** calculated at the B3LYP/6-311G(d,p) level

Table S4. Cartesian coordinates for the re-optimized conformers of (1*R*,3*R*,4*S*,7*R*)-**5**

Conf. 1	Coordinates (Angstroms)		
Atom	X	Y	Z
C	2.23976	-0.31208	1.67836

C	2.11541	0.89146	0.73456
C	1.2356	2.05193	1.16156
C	0.78384	2.99746	0.05329
C	-0.71318	3.40136	0.09693
C	-1.65314	2.21942	0.11852
C	2.68402	-1.44087	0.72921
C	2.17128	-1.004	-0.67386
C	1.03772	-1.86404	-1.30665
C	-2.16834	1.54842	-0.92459
C	-3.00765	0.30362	-0.69576
C	-2.23779	-1.01669	-0.94528
C	-0.11283	-2.36244	-0.39855
C	-1.14534	-1.31876	0.11699
C	-1.75723	-1.77309	1.43946
C	-2.47085	-3.10611	1.47499
C	-1.66305	-1.01996	2.5431
C	-1.94508	1.92877	-2.36987
C	0.95445	2.26642	2.45098
O	0.54413	-1.16696	-2.45411
O	1.6039	0.3178	-0.48026
C	3.33357	-0.89167	-1.67324
H	2.95249	-0.14219	2.49121
H	1.26385	-0.53649	2.11958
H	3.12553	1.29102	0.52444
H	1.39596	3.911	0.08788
H	0.98416	2.52643	-0.91216
H	-0.90638	4.04938	-0.76642
H	-0.89822	4.02038	0.98415
H	-1.89423	1.84434	1.11178
H	2.29868	-2.41577	1.03873
H	3.77616	-1.52159	0.71088
H	1.52304	-2.76498	-1.70485
H	-3.39508	0.30148	0.32996
H	-3.88058	0.32353	-1.36439
H	-2.95868	-1.84446	-0.97675
H	-1.76925	-0.99694	-1.93317
H	-0.64208	-3.13407	-0.97352
H	0.33317	-2.88518	0.45709
H	-0.61188	-0.38505	0.31076
H	-3.33115	-3.12577	0.79387
H	-1.81091	-3.92659	1.16479
H	-2.83761	-3.33153	2.48112
H	-2.09282	-1.33894	3.49053
H	-1.15677	-0.0581	2.53924

H	-1.31296	2.81283	-2.48705
H	-1.47851	1.11018	-2.93459
H	-2.90396	2.13196	-2.86719
H	0.37253	3.12705	2.77121
H	1.30032	1.60577	3.24017
H	0.40614	-0.25114	-2.15546
H	4.06793	-0.15788	-1.32304
H	3.84511	-1.85597	-1.78014
H	2.96482	-0.58438	-2.65457

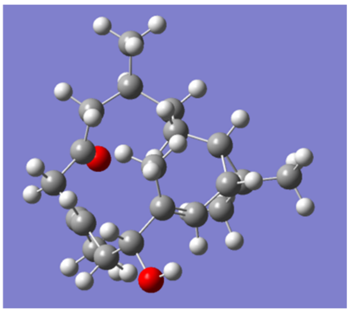

Conf. 1
0.02%
-931.784164 a. u.

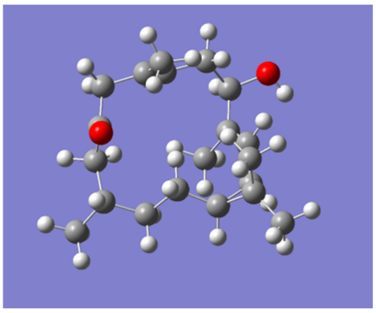

Conf. 2
0.34%
-931.786737 a. u.

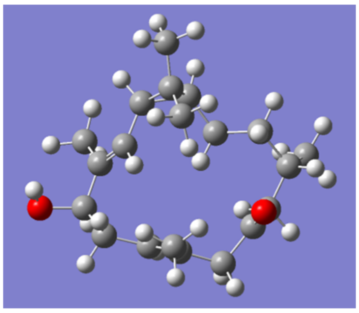

Conf. 3
1.31%
-931.787785 a. u.

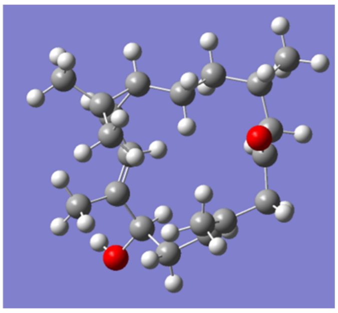

Conf. 4
3.95%
-931.788647 a. u.

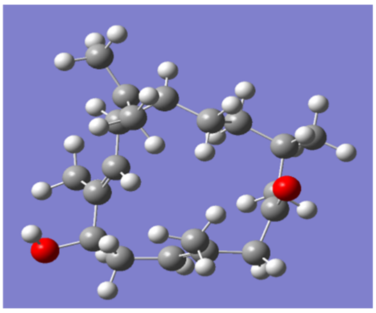

Conf. 5
93.57%
-931.791269 a. u.

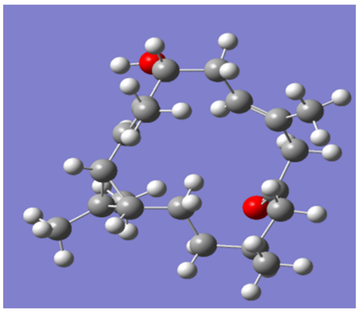

Conf. 6
0.25%
-931.786089 a. u.

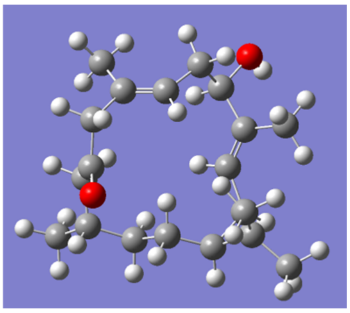

Conf. 7
0.31%
-931.785776 a. u.

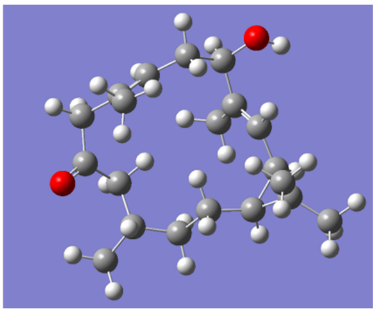

Conf. 8
0.25%
-931.786013 a. u.

Figure S69. Re-optimized conformers of (1*S*,2*R*,5*R*,12*S*)-**7** calculated at the B3LYP/6-311G(d,p) level

Table S5. Cartesian coordinates for the re-optimized conformers of (1*S*,2*R*,5*R*,12*S*)-**7**

Conf. 1	Coordinates (Angstroms)		
Atom	X	Y	Z
C	-2.25107500	-2.28287300	0.10324500
C	-0.79475300	-2.53633000	-0.18587100
C	-2.24846600	0.87131600	-0.11400100
C	-2.30410300	-0.01235400	-1.12351500
C	-2.97178900	-1.37248800	-0.91171400
C	0.84082300	1.34042500	-0.64683800
C	-0.12016000	2.46643800	-0.30317800
C	-1.62273900	2.23123200	-0.11812400
C	0.20144300	-2.63443200	0.71228600
C	1.63074500	-2.91115700	0.25810500
C	2.50222200	-1.65429700	0.41186700
C	2.83724600	-0.89521100	-0.86398400
C	3.31836000	0.55441200	-0.67234300
C	2.24559200	1.47457100	-0.03252900
O	-4.30578300	-1.25349800	-0.40237100
C	-1.76141800	0.24211200	-2.50737100
C	0.03590000	-2.51732700	2.20795200
O	2.90130600	-1.29446300	1.50568600
C	3.82863100	1.11670800	-2.00933800
C	-0.76196700	2.68118600	1.05568900
C	-0.92332000	4.11831200	1.52602200
C	-0.49743600	1.70675900	2.19244500
H	-2.26053900	3.01226700	-0.53759500
H	0.12113300	3.39569800	-0.82196100
H	-2.80583000	-3.23333200	0.12025800
H	-2.38623800	-1.83387200	1.09141000
H	-0.54414200	-2.69312900	-1.23713300
H	-2.72057300	0.57461900	0.82391300
H	-2.99748800	-1.89228600	-1.88513100
H	0.92270600	1.30435300	-1.74232400
H	0.40479100	0.38144300	-0.35062800
H	1.64174300	-3.25370900	-0.78199100
H	2.07383300	-3.69401800	0.88717300
H	1.97787600	-0.94524700	-1.54457200
H	3.62623700	-1.48320300	-1.36220700
H	4.16557200	0.52171400	0.02422300
H	2.18876800	1.26017600	1.03958700
H	2.59202900	2.51379600	-0.12258200
H	-4.72099100	-0.50656400	-0.86076900
H	-1.34485500	1.24628400	-2.60827000
H	-0.97794700	-0.48178900	-2.77114400
H	-2.55420500	0.12420400	-3.25986400
H	0.16381400	-3.50317900	2.67784600
H	0.80878200	-1.86852100	2.63313600
H	-0.94499600	-2.13707300	2.50101500
H	3.02878300	1.18010900	-2.75791100
H	4.23820300	2.12480300	-1.87684700
H	4.62196800	0.48686700	-2.43037400
H	-1.75466300	4.21052500	2.23765200
H	-1.12595400	4.79603000	0.68852800
H	-0.01481700	4.47339600	2.03010300
H	-1.33912900	1.69715400	2.89795000
H	-0.34717800	0.68129200	1.84459400
H	0.39669600	2.00287100	2.75457100

Conf. 2	Coordinates (Angstroms)		
Atom	X	Y	Z
C	-1.37829200	-2.93115400	-0.01034800
C	0.11472700	-2.77059300	-0.07856700
C	-2.28475200	0.13336000	-0.36450600
C	-1.84393400	-0.73786600	-1.28662500
C	-2.15234200	-2.22868400	-1.14552800
C	0.51261200	1.66635000	0.21575500
C	-0.81594400	2.35951500	-0.07300900
C	-2.12606700	1.61987500	-0.38428200
C	0.94541900	-2.45312000	0.92925600
C	2.43876900	-2.29505000	0.67162000
C	2.80939700	-0.80538200	0.68683600
C	2.96887100	-0.13238500	-0.67134000
C	2.97257700	1.40849900	-0.65405100
C	1.56546500	2.00810600	-0.85367900
O	-3.53012600	-2.47701900	-0.84673200
C	-1.06734200	-0.33955200	-2.51535900
C	0.53388400	-2.22097500	2.36239900
O	2.96820700	-0.20897400	1.73689900

C	3.93126600	1.95963100	-1.72211000
C	-1.97458800	2.41348700	0.90306000
C	-2.73902600	3.72285500	1.01715200
C	-1.85972100	1.66644900	2.22198100
H	-2.73295600	2.11082400	-1.14775800
H	-0.69369300	3.27584500	-0.65272500
H	-1.65047700	-3.99634900	-0.05565800
H	-1.77760500	-2.54982900	0.93340900
H	0.55944500	-2.95514400	-1.05883000
H	-2.86052700	-0.27730800	0.46575200
H	-1.88941000	-2.71977800	-2.09876900
H	0.36551700	0.58161100	0.26932700
H	0.89628000	1.97442400	1.19780000
H	2.71125700	-2.74369800	-0.28964800
H	3.01428600	-2.78674700	1.46559800
H	2.20664700	-0.52753800	-1.35700400
H	3.92699200	-0.51160600	-1.06381900
H	3.34168300	1.71566500	0.33278600
H	1.66817500	3.10219900	-0.89644400
H	1.19136800	1.70502100	-1.84336700
H	-4.05336900	-1.86524100	-1.38769700
H	-1.61537900	-0.60991200	-3.42949600
H	-0.86754400	0.73370400	-2.54353500
H	-0.10545300	-0.86793200	-2.56735900
H	0.98917600	-1.30383700	2.75140900
H	-0.54850300	-2.15865200	2.49247400
H	0.89862100	-3.04224600	2.99578400
H	3.62313000	1.65468400	-2.73110400
H	3.95660700	3.05543500	-1.70210000
H	4.95470500	1.59922400	-1.56312400
H	-3.76336500	3.55370400	1.37523100
H	-2.80520200	4.23519100	0.05027200
H	-2.24963800	4.40636700	1.72359800
H	-2.85313200	1.42473200	2.62240800
H	-1.30169100	0.73045700	2.12966000
H	-1.34645800	2.28448000	2.96965000

Conf. 3	Coordinates (Angstroms)		
Atom	X	Y	Z
C	-2.46779700	-2.05390700	0.15694100
C	-1.03169900	-2.38634400	-0.16464100
C	-1.79861600	0.83905500	0.02345500
C	-2.63423200	0.33887900	-0.90330700
C	-3.14643800	-1.09326400	-0.84097000
C	1.10633000	1.28261300	-0.73872500
C	0.25614900	2.44300900	-0.24758100
C	-1.23202400	2.21287900	0.07714400
C	-0.02481200	-2.62234500	0.69399200
C	1.36060100	-2.99171000	0.17267300
C	2.33849600	-1.80953100	0.28648600
C	2.78414100	-1.17210500	-1.02168100
C	3.46453000	0.20529700	-0.91540600
C	2.56615600	1.28672700	-0.25827500
O	-4.56966600	-1.09718400	-0.62099900
C	-3.17129600	1.12539000	-2.07516900
C	-0.13714400	-2.57620400	2.19797800
O	2.73324400	-1.41954200	1.37193200
C	3.96116800	0.64237000	-2.30354200
C	-0.24717300	2.62146500	1.16894000
C	-0.36651800	4.05216400	1.67374100
C	0.13591900	1.64289400	2.26865300
H	-1.89997400	3.00244200	-0.26242600
H	0.46349000	3.37928500	-0.76741700
H	-3.07120700	-2.97242400	0.12280900
H	-2.57492100	-1.66451900	1.17631500
H	-0.81817700	-2.48748100	-1.23081600
H	-1.46420700	0.16605000	0.80655100
H	-3.04393000	-1.53249400	-1.84350900
H	1.06959600	1.29294200	-1.83819000
H	0.63467800	0.34001800	-0.44482500
H	1.29942900	-3.32953700	-0.86687500
H	1.77278500	-3.81227400	0.77493300
H	1.92511200	-1.13872000	-1.70463400
H	3.47956500	-1.89211300	-1.48479500
H	4.34195300	0.08399000	-0.26721000
H	2.57995300	1.14194000	0.82606300
H	3.01757200	2.27096000	-0.44710800
H	-4.72145200	-0.58386800	0.18974500
H	-2.91647500	2.18661000	-2.02497400
H	-2.76878500	0.73272600	-3.02012100
H	-4.26118600	1.02704200	-2.13408400
H	-0.06835200	-3.59205700	2.61332900
H	0.69394200	-2.00663900	2.62755900
H	-1.07903900	-2.14255500	2.54292300
H	3.12888600	0.79717900	-3.00178900
H	4.51849700	1.58393400	-2.23884800
H	4.62604400	-0.10931400	-2.74669200

H	-1.11762200	4.12734900	2.47109800
H	-0.66189200	4.73962800	0.87274000
H	0.58904700	4.40634200	2.08214700
H	-0.66736200	1.55955200	3.01277700
H	0.35889600	0.63854500	1.90110500
H	1.03207500	1.99727600	2.79251400

Conf. 4	Coordinates (Angstroms)		
Atom	X	Y	Z
C	-1.82371700	-2.69922000	-0.27255000
C	-0.32493800	-2.73355500	-0.14834400
C	-1.47568500	0.65115600	-1.10113000
C	-2.41823900	-0.24697000	-0.77140400
C	-2.31318800	-1.66605000	-1.31569900
C	1.12382100	1.71401200	0.14705800
C	-0.04800200	2.63535200	-0.15160800
C	-1.36120200	2.08941900	-0.70779400
C	0.44421700	-2.51106500	0.92991800
C	1.96078400	-2.62636400	0.81449800
C	2.61109500	-1.23668500	0.74870200
C	2.96982700	-0.73403300	-0.64569200
C	3.36789100	0.75066700	-0.75782500
C	2.15377700	1.67427400	-0.99206400
O	-3.57783300	-2.15149900	-1.78489900
C	-3.60707500	0.04698700	0.10992100
C	-0.05569300	-2.14789900	2.30547100
O	2.82878400	-0.59560600	1.76083700
C	4.40113100	0.94422800	-1.87960600
C	-1.29394700	2.64788800	0.71290000
C	-1.95826700	3.99836400	0.93719900
C	-1.35971800	1.74721900	1.93600000
H	-1.85493500	2.75845100	-1.41681300
H	0.25363900	3.60952900	-0.54007300
H	-2.17200300	-3.67683100	-0.63400600
H	-2.31753600	-2.52768700	0.68827300
H	0.18598500	-3.00340800	-1.07541700
H	-0.69484300	0.31122400	-1.78586900
H	-1.59104700	-1.66568100	-2.14827000
H	0.75237800	0.70279900	0.33993900
H	1.62535200	2.03190800	1.07109300
H	2.22823000	-3.20617400	-0.07529600
H	2.36563000	-3.13508200	1.69780500
H	2.14425300	-0.98054200	-1.32889900
H	3.80517900	-1.37538300	-0.97187300
H	3.83399200	1.03249100	0.19536500
H	2.52478700	2.69484500	-1.16566100
H	1.65553200	1.37521300	-1.92768000
H	-3.89985700	-1.51872200	-2.44666800
H	-3.69194800	1.11356100	0.32562000
H	-4.53137700	-0.29230700	-0.36979900
H	-3.54527400	-0.48939100	1.06614400
H	0.12439800	-2.97416800	3.00773600
H	0.49279000	-1.28345600	2.69695100
H	-1.12376100	-1.91946300	2.32301800
H	3.99089500	0.65019000	-2.85488900
H	4.70965200	1.99345100	-1.95443200
H	5.30123500	0.34331100	-1.70235100
H	-3.02751300	3.88373300	1.16088400
H	-1.86936500	4.64427500	0.05604800
H	-1.49845100	4.52524600	1.78375100
H	-0.73430700	2.15318500	2.74168300
H	-2.38531200	1.68546500	2.31902200
H	-1.02124100	0.72991700	1.72944600

Conf. 5	Coordinates (Angstroms)		
Atom	X	Y	Z
C	-1.64260000	-2.66280400	0.19632900
C	-0.14263300	-2.65403900	0.06595600
C	-1.75101400	0.29026000	-0.20929000
C	-2.27532200	-0.50453500	-1.16102000
C	-2.38862100	-2.01365700	-0.98699500
C	0.96600600	1.79667900	0.23335500
C	-0.35685100	2.52299200	0.04591900
C	-1.67312100	1.76937500	-0.21581000
C	0.77806700	-2.44191500	1.02072200
C	2.26211900	-2.48915500	0.67508300
C	2.80621400	-1.06122400	0.53192900
C	2.90266000	-0.52743600	-0.89146300
C	3.10501900	0.99234100	-1.04215700
C	1.76748500	1.76127600	-1.07865600
O	-3.77906900	-2.38864600	-0.93737300
C	-2.85383300	-0.01485800	-2.46841500
C	0.48407500	-2.13302300	2.46791400
O	3.13494400	-0.41096900	1.50808700
C	3.91940400	1.29661800	-2.31046800
C	-1.46294300	2.57535900	1.07123600

C	-2.24714200	3.87471500	1.18007900
C	-1.30384500	1.87435500	2.41149900
H	-2.32291000	2.25355700	-0.94057300
H	-0.25413500	3.43466400	-0.54547700
H	-1.99817500	-3.70286200	0.21849500
H	-1.97385800	-2.21215900	1.13907200
H	0.22091300	-2.89317200	-0.93582500
H	-1.34102300	-0.19420600	0.67078000
H	-2.03006100	-2.49422000	-1.90829200
H	0.79408300	0.77853500	0.59206400
H	1.56549100	2.29439800	1.00812800
H	2.41906100	-3.04656400	-0.25444500
H	2.82251200	-2.98099800	1.47898800
H	2.02303100	-0.86828500	-1.45529900
H	3.75323100	-1.06914100	-1.33768500
H	3.67956100	1.33297600	-0.17088700
H	1.97390400	2.79839900	-1.38053300
H	1.13617100	1.33723200	-1.87423600
H	-4.17682200	-1.86465000	-0.22237000
H	-2.92272700	1.07347100	-2.52787500
H	-2.23386400	-0.35212400	-3.31169500
H	-3.85232800	-0.43750100	-2.62546100
H	0.76817900	-2.98351900	3.10379700
H	1.08111100	-1.27869700	2.80780400
H	-0.57163300	-1.92077900	2.65478000
H	3.39239700	0.95923900	-3.21285600
H	4.09786700	2.37299900	-2.41569400
H	4.89522600	0.79666100	-2.28840900
H	-3.25390500	3.69663000	1.58042400
H	-2.35767400	4.36205300	0.20462800
H	-1.74302500	4.58287100	1.85111400
H	-2.27963400	1.57036700	2.81211600
H	-0.66868800	0.98642900	2.36567600
H	-0.84802000	2.55735200	3.13954600

Conf. 6	Coordinates (Angstroms)		
Atom	X	Y	Z
C	0.64755100	3.28962400	0.25058700
C	1.27495000	2.20234400	-0.58425200
C	-2.06894200	1.08529800	0.00159000
C	-1.51270800	2.00584000	0.80584600
C	-0.89792700	3.27901100	0.23653800
C	-0.43394300	-1.45995700	0.77614400
C	-1.95272000	-1.45982500	0.74345000
C	-2.74068800	-0.18142100	0.42428000
C	2.44336600	1.57560300	-0.36986100
C	2.92769000	0.52359900	-1.36546400
C	2.33903000	-0.88292900	-1.16235200
C	2.50194400	-1.52474300	0.20899400
C	1.74714800	-2.84711300	0.44288600
C	0.21454400	-2.74039700	0.22463200
O	-1.27896900	3.54222500	-1.11116800
C	-1.45884500	1.87880000	2.30755800
C	3.37584200	1.87108300	0.78128600
O	1.78926200	-1.45535800	-2.08659200
C	2.08343900	-3.38237900	1.84496900
C	-2.79806200	-1.37830900	-0.51380800
C	-4.09111400	-2.17965800	-0.52506200
C	-2.14679600	-1.28404700	-1.88532100
H	-3.63008000	-0.03303200	1.03857800
H	-2.39640600	-2.07185200	1.53018500
H	0.98877800	3.25244600	1.29046200
H	0.94816000	4.27297500	-0.14048000
H	0.71773600	1.94963800	-1.48509100
H	-2.03156300	1.27766000	-1.06918200
H	-1.22094400	4.11890100	0.87869800
H	-0.12478900	-1.29810100	1.81979800
H	-0.05972800	-0.59300000	0.22288600
H	4.02310100	0.43820300	-1.30710000
H	2.67764400	0.80808400	-2.39136000
H	2.22302800	-0.78481100	0.96944500
H	3.58183500	-1.68833800	0.35515800
H	2.12273700	-3.57201100	-0.29111300
H	0.00975200	-2.79851600	-0.84728600
H	-0.25897900	-3.62010500	0.68356900
H	-2.24381200	3.44786400	-1.15844600
H	-1.93210700	0.95657900	2.65225300
H	-0.42255000	1.87735000	2.67232800
H	-1.96011700	2.72601800	2.79648000
H	2.94212100	2.54945100	1.52009000
H	3.68007200	0.95538600	1.30427300
H	4.30073100	2.33581200	0.41016600
H	1.70998600	-2.71461100	2.63201600
H	1.63070300	-4.36770600	2.00488300
H	3.16637800	-3.48522900	1.98798500
H	-4.82223900	-1.73977900	-1.21648300
H	-4.55292000	-2.21465600	0.46860300

H	-3.91164400	-3.21408700	-0.84620700
H	-2.78503000	-0.72003800	-2.57910200
H	-1.16567900	-0.80278600	-1.86848900
H	-2.00906700	-2.28519300	-2.31128700

Conf. 7	Coordinates (Angstroms)		
Atom	X	Y	Z
C	0.74721500	3.13384100	0.28407500
C	-0.31889800	2.32245000	0.97726000
C	1.60221000	-0.03575300	-1.00912100
C	2.11620600	1.12718900	-0.57628700
C	1.38933000	2.41774800	-0.92425600
C	-0.27474500	-2.16365600	0.08815000
C	1.14317100	-2.51567900	-0.33140800
C	2.10251800	-1.43126400	-0.81816900
C	-1.63921200	2.32651900	0.72813900
C	-2.56648400	1.43582500	1.54782300
C	-2.63539100	-0.04635800	1.13688400
C	-2.83957000	-0.37252800	-0.34029200
C	-2.73548800	-1.86547200	-0.71846300
C	-1.29062900	-2.29183500	-1.05805800
O	2.27143600	3.38248200	-1.51352900
C	3.38689800	1.26102100	0.22522300
C	-2.30845100	3.19093600	-0.31338400
O	-2.58630600	-0.91108500	1.99176000
C	-3.66769600	-2.18397000	-1.89853200
C	2.34398000	-2.13690600	0.51512300
C	3.49593600	-3.13060000	0.54890000
C	2.13574500	-1.43303100	1.84690800
H	2.77725000	-1.75330400	-1.61493700
H	1.22239800	-3.47586000	-0.84378700
H	0.35061300	4.08323900	-0.09093100
H	1.53949300	3.39240100	0.99688800
H	0.03712900	1.65704400	1.76463100
H	0.69839900	0.03228500	-1.61874300
H	0.57878000	2.17599000	-1.62985300
H	-0.29173100	-1.14134200	0.47595800
H	-0.59076000	-2.80396200	0.92242400
H	-3.59341100	1.82800700	1.49499800
H	-2.28223900	1.43577100	2.60410000
H	-2.14522400	0.23449800	-0.93648400
H	-3.84215100	0.01079500	-0.59005400
H	-3.06813100	-2.44383700	0.15266000
H	-1.30868100	-3.33714600	-1.39902700
H	-0.94443800	-1.70365400	-1.92216800
H	2.68418600	2.95776300	-2.28250100
H	3.94608800	0.32332700	0.25281100
H	4.02301300	2.04189700	-0.20593400
H	3.18284700	1.56299300	1.26116700
H	-2.87583100	2.58184200	-1.02990500
H	-3.03440900	3.86824200	0.15816600
H	-1.60204700	3.79866100	-0.88344000
H	-3.39312300	-1.60659000	-2.79143500
H	-3.61762000	-3.24620100	-2.16476800
H	-4.71169400	-1.94908000	-1.65836800
H	4.44688800	-2.62963100	0.77488200
H	3.61151900	-3.64862100	-0.41016200
H	3.33243600	-3.89340800	1.32153600
H	3.07803300	-1.00564700	2.21014200
H	1.40577000	-0.62261800	1.78865300
H	1.78573100	-2.14588500	2.60453500

Conf. 8	Coordinates (Angstroms)		
Atom	X	Y	Z
C	-0.10450800	3.13454800	0.28522500
C	-1.40154300	2.49460200	-0.12829900
C	2.21788300	0.82857400	-0.23343900
C	1.42293100	1.52754000	-1.06205500
C	1.04532600	2.97315600	-0.73231800
C	0.58674600	-1.87755800	0.34956700
C	2.02060300	-1.84581600	-0.16409900
C	2.77115500	-0.53910700	-0.47496600
C	-2.27821900	1.83982000	0.65144500
C	-3.50826800	1.18098200	0.04627900
C	-3.40526800	-0.35233200	0.12673400
C	-2.31684400	-0.98943700	-0.72474000
C	-1.86249000	-2.39497400	-0.27891100
C	-0.37109300	-2.60115200	-0.61774700
O	2.13199600	3.69952700	-0.15269400
C	0.91154000	0.98995300	-2.37510900
C	-2.14269200	1.66034800	2.14440200
O	-4.13339400	-0.99763400	0.85814700
C	-2.75231500	-3.48699200	-0.88805400
C	3.21286000	-1.43186900	0.67345900
C	4.50352800	-2.21574600	0.49451700
C	2.98432900	-0.96901300	2.10328500

H	3.40015300	-0.60109900	-1.36440700
H	2.23709000	-2.64838400	-0.87061700
H	-0.23808900	4.21781200	0.42514400
H	0.25143600	2.73913800	1.24145700
H	-1.65156900	2.59278700	-1.18651500
H	2.54217500	1.33228800	0.67708500
H	0.73685400	3.46062400	-1.67455600
H	0.23509400	-0.85367800	0.52672300
H	0.54023400	-2.38835300	1.32209100
H	-3.60965700	1.47624300	-1.00615000
H	-4.42055200	1.46958200	0.58095300
H	-1.47246800	-0.29320100	-0.75182600
H	-2.69414200	-1.02577900	-1.76006700
H	-1.97630900	-2.45165200	0.81245700
H	-0.13701900	-3.67491900	-0.60487900
H	-0.18474200	-2.26491600	-1.64879400
H	2.93055600	3.45822600	-0.64794800

H	1.16595400	-0.06400100	-2.51120500
H	-0.17909100	1.08715900	-2.45037400
H	1.33040100	1.55491800	-3.22043200
H	-2.97063900	2.16453300	2.66168600
H	-2.20649400	0.60203200	2.42776600
H	-1.20903000	2.06529500	2.53987800
H	-2.66255300	-3.50382300	-1.98281800
H	-2.47138200	-4.47942800	-0.51608000
H	-3.80238200	-3.31395000	-0.63233800
H	5.37624800	-1.61120500	0.77516300
H	4.63963600	-2.53347200	-0.54570700
H	4.50887000	-3.11718800	1.12145100
H	3.79882200	-0.31318300	2.43759800
H	2.04576600	-0.42131000	2.22652400
H	2.95603900	-1.82939600	2.78405400

9. Origin spectra of known compounds **6** and **8-16.**

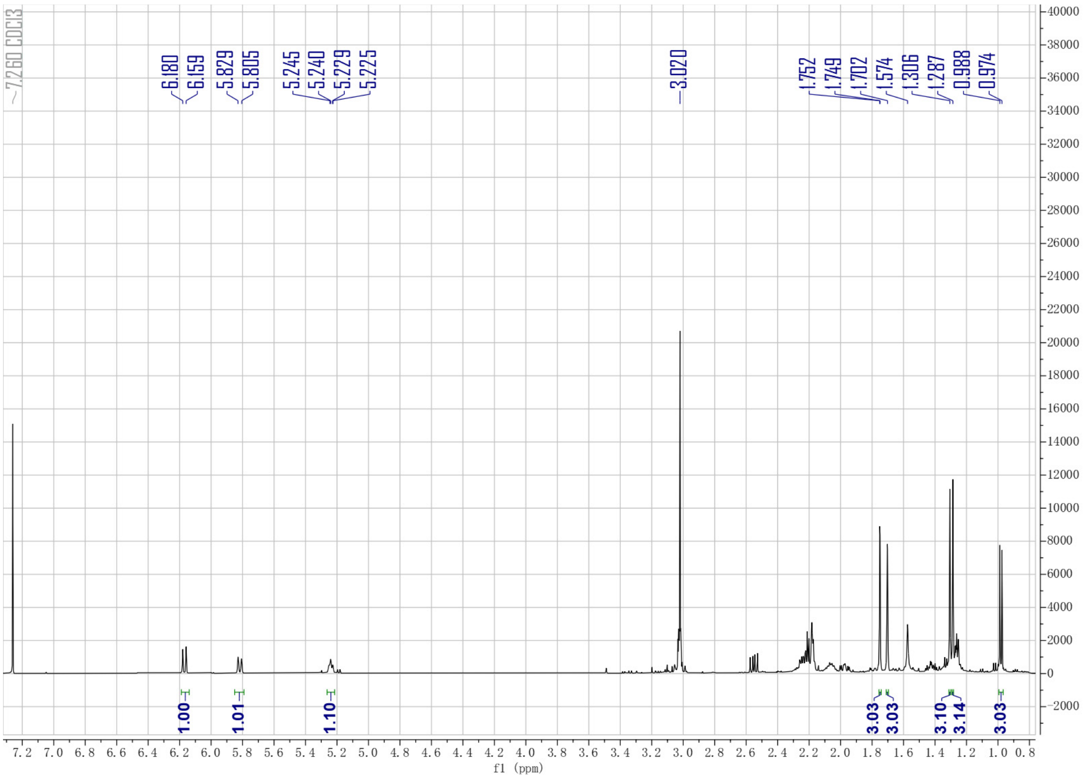

Figure S70. ^{1}H NMR spectrum (500 MHz) of **6** in $CDCl_3$.

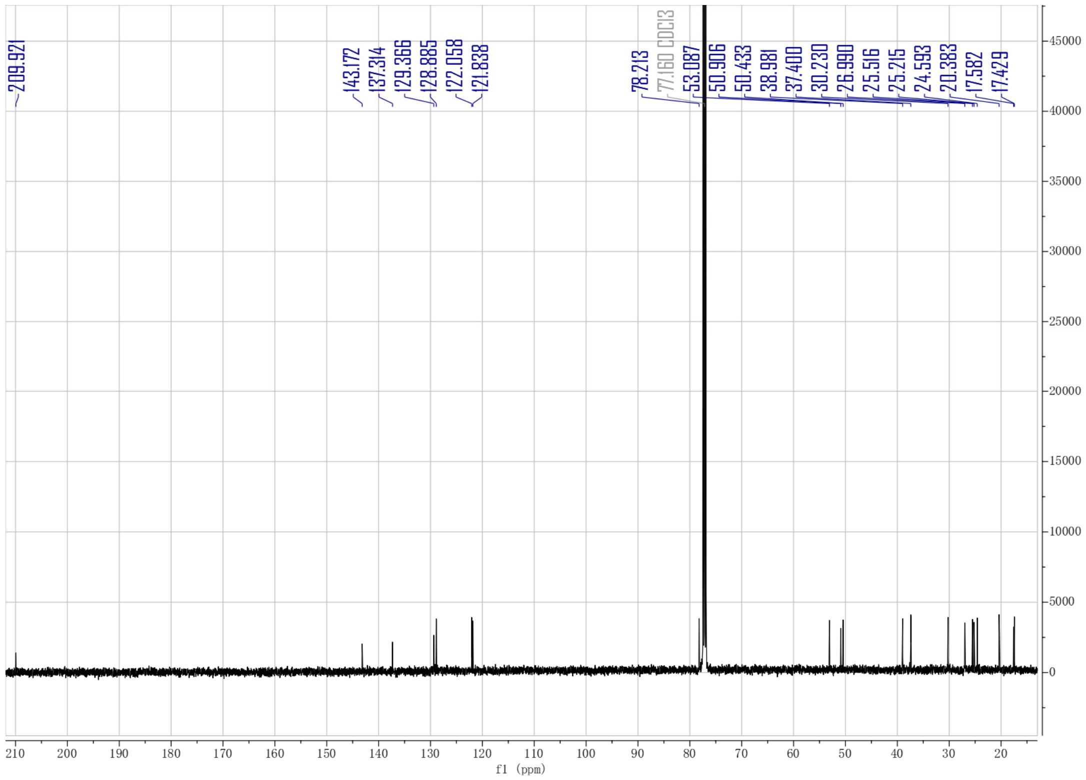

Figure S71. ^{13}C NMR spectrum (125 MHz) of **6** in $CDCl_3$.

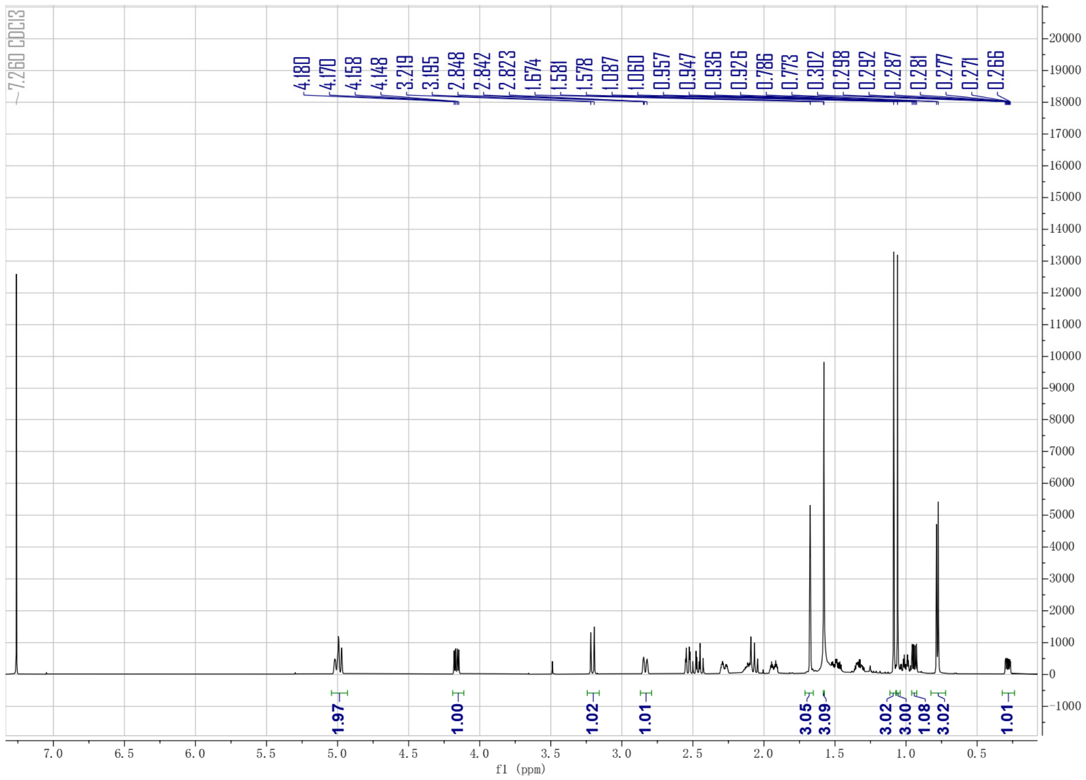

Figure S72. ^{1}H NMR spectrum (500 MHz) of **8** in $CDCl_3$.

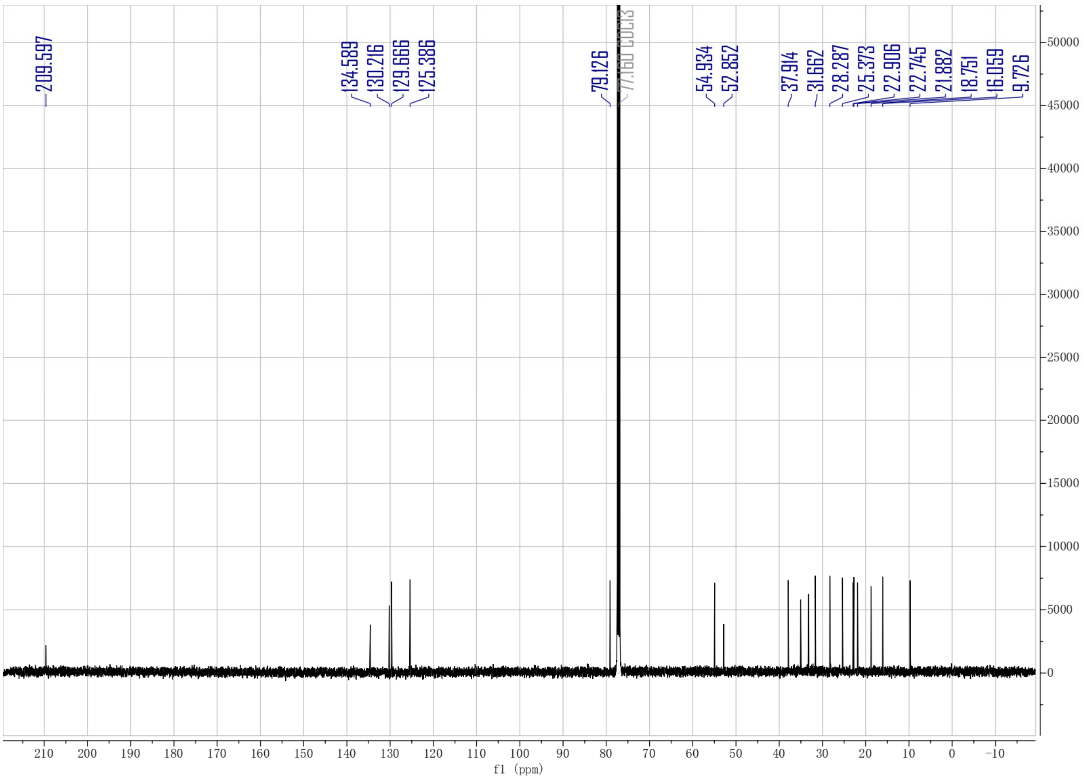

Figure S73. ^{13}C NMR spectrum (125 MHz) of **8** in $CDCl_3$.

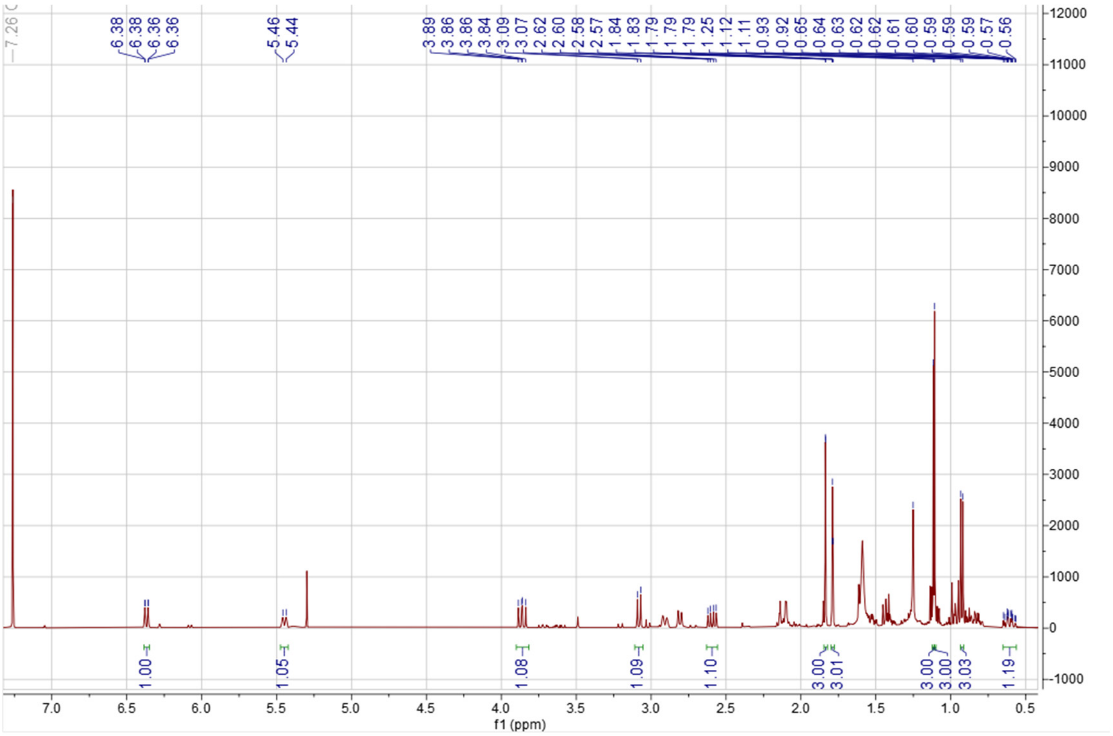

Figure S74. ^{1}H NMR spectrum (500 MHz) of **9** in $CDCl_3$.

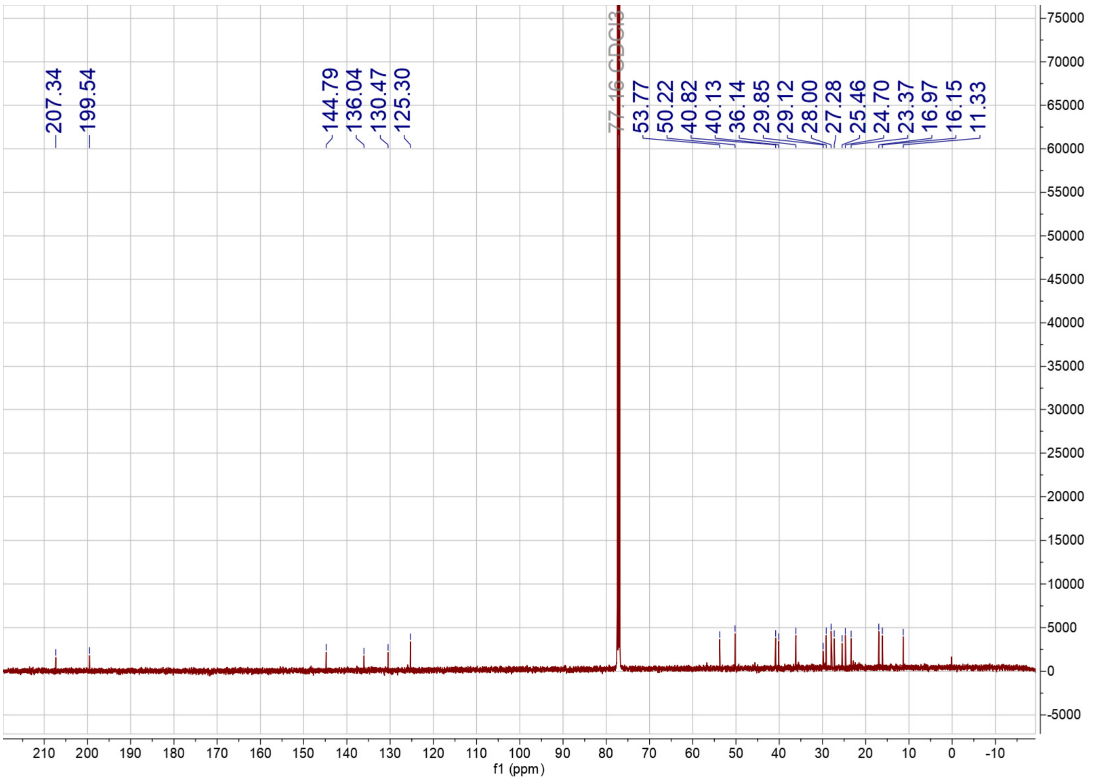

Figure S75. ^{13}C NMR spectrum (125 MHz) of **9** in $CDCl_3$.

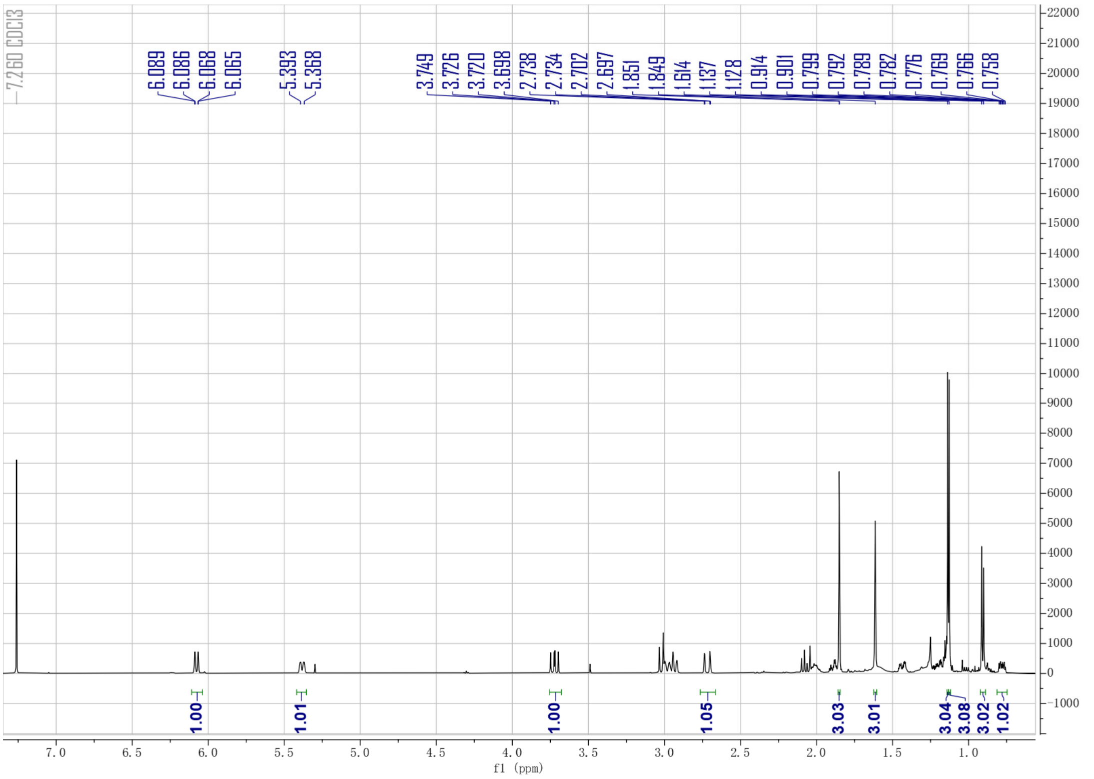

Figure S76. ^{1}H NMR spectrum (500 MHz) of **10** in $CDCl_3$.

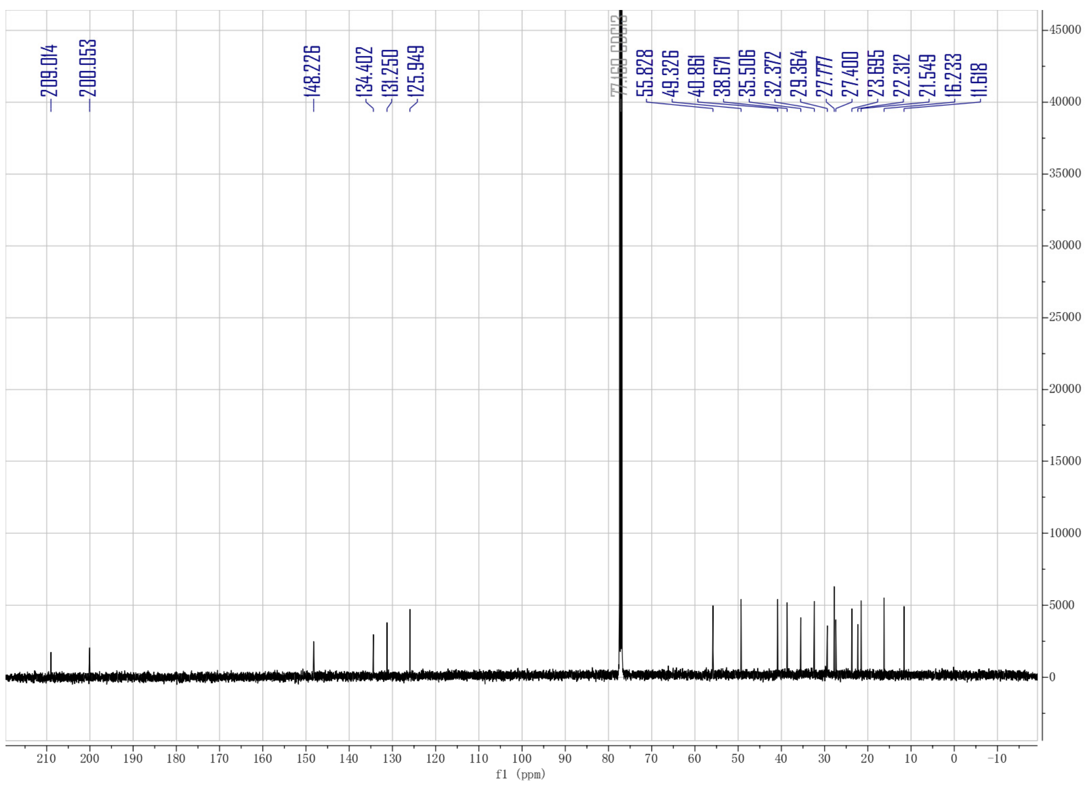

Figure S77. ^{13}C NMR spectrum (125 MHz) of **10** in $CDCl_3$.

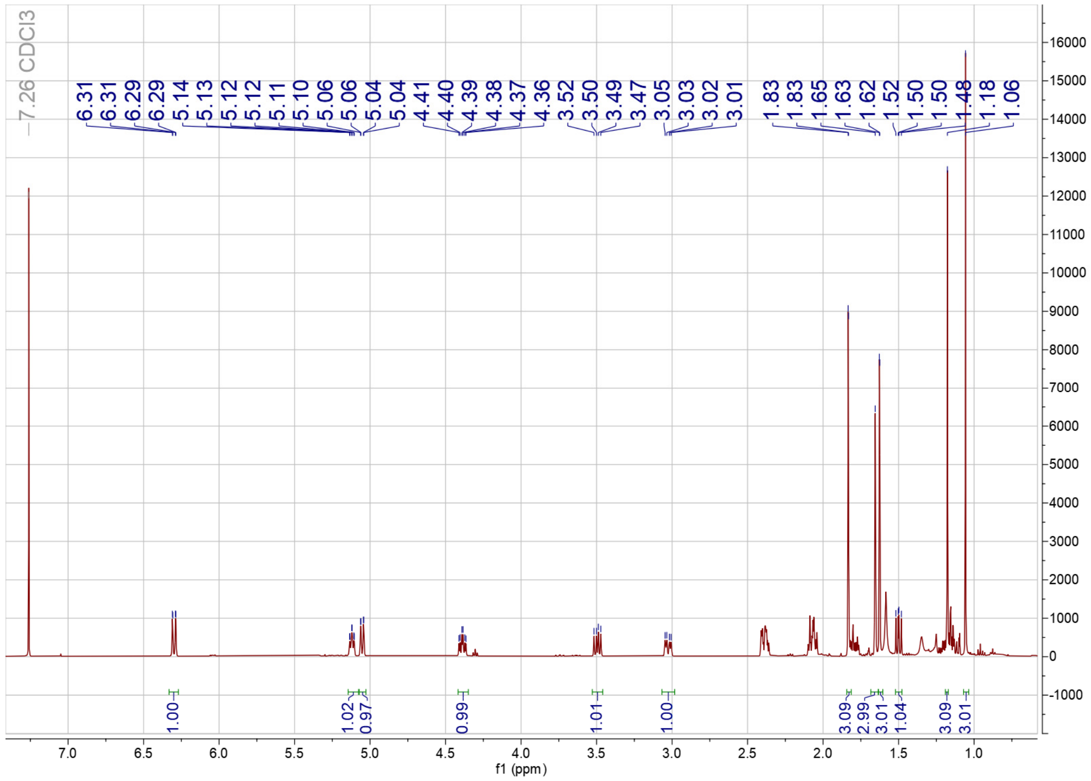

Figure S78. ^{1}H NMR spectrum (500 MHz) of **11** in $CDCl_3$.

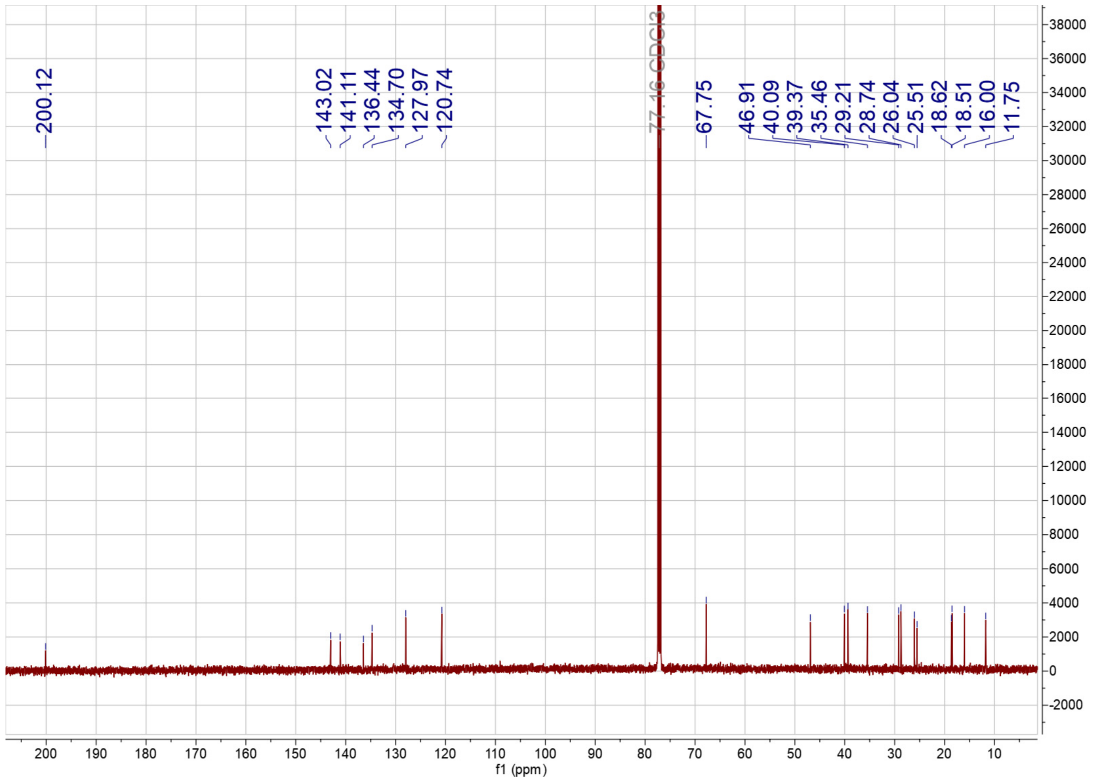

Figure S79. ^{13}C NMR spectrum (125 MHz) of **11** in $CDCl_3$.

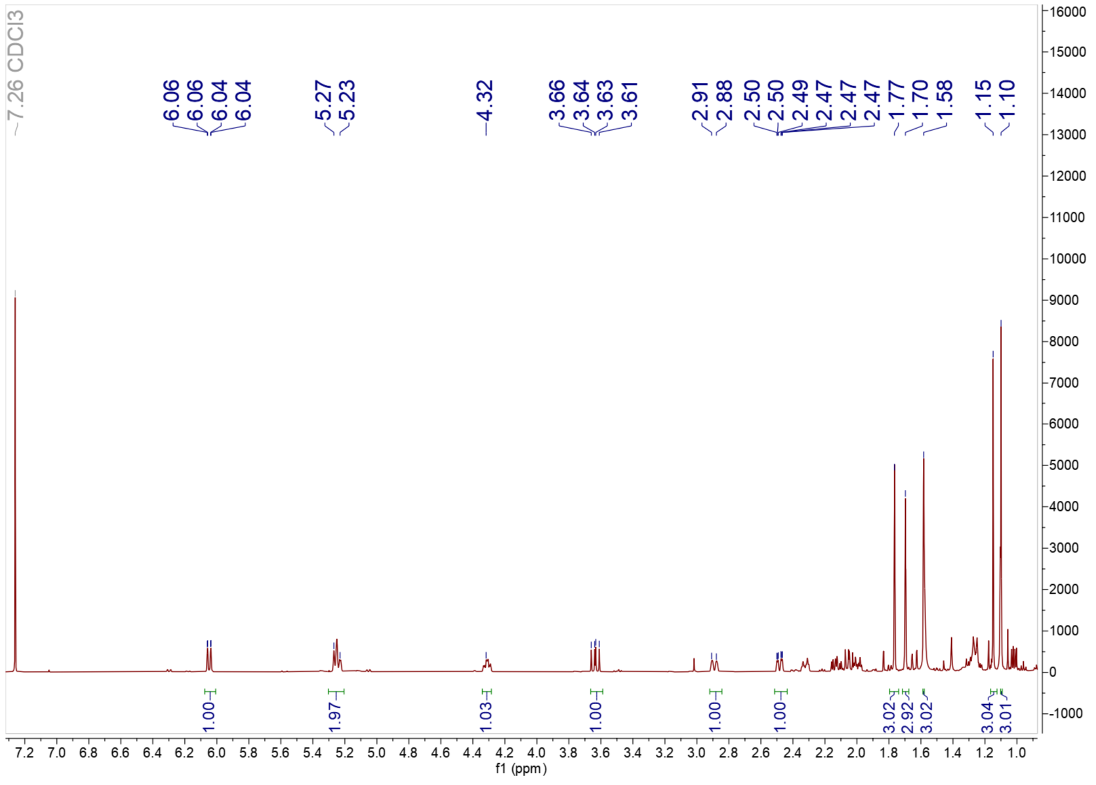

Figure S80. ^{1}H NMR spectrum (500 MHz) of **12** in $CDCl_3$.

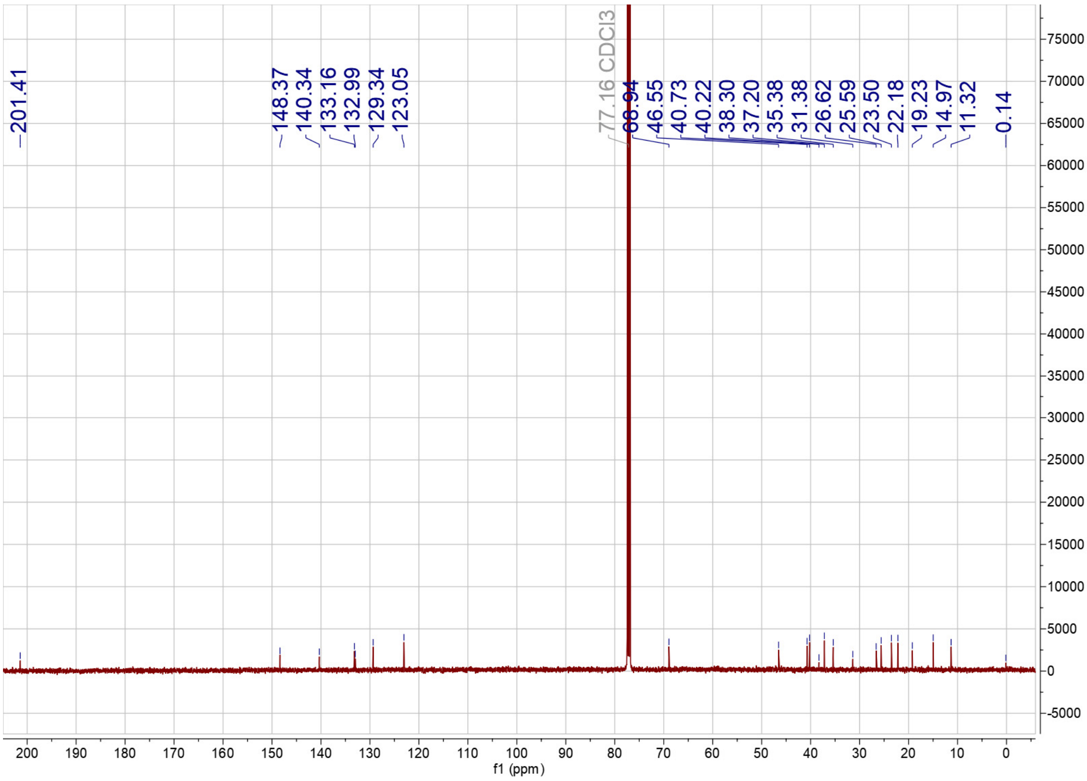

Figure S81. ^{13}C NMR spectrum (125 MHz) of **12** in $CDCl_3$.

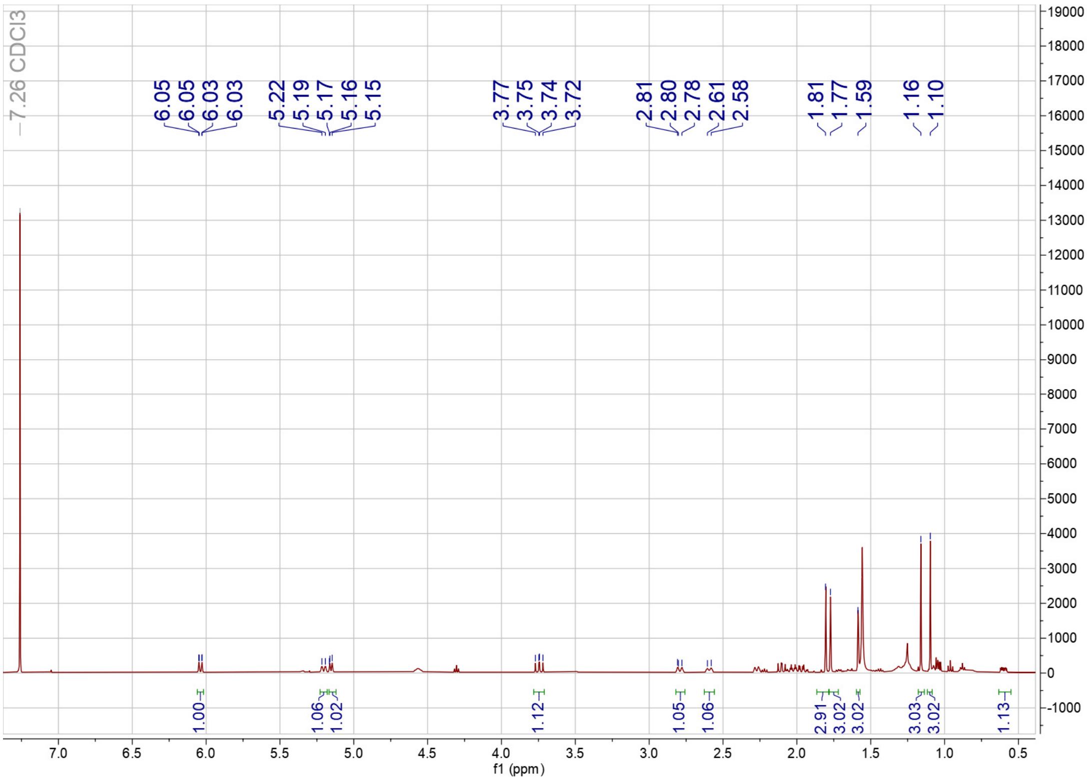

Figure S82. ^{1}H NMR spectrum (500 MHz) of **13** in $CDCl_3$.

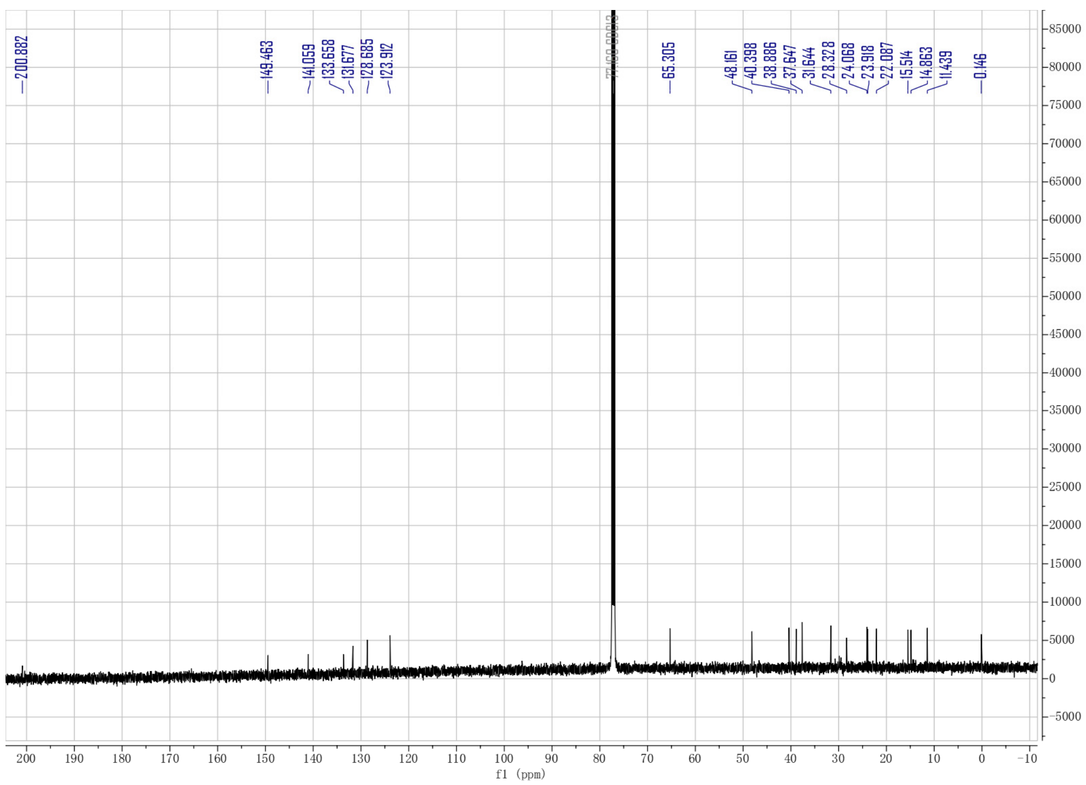

Figure S83. ^{13}C NMR spectrum (125 MHz) of **13** in $CDCl_3$.

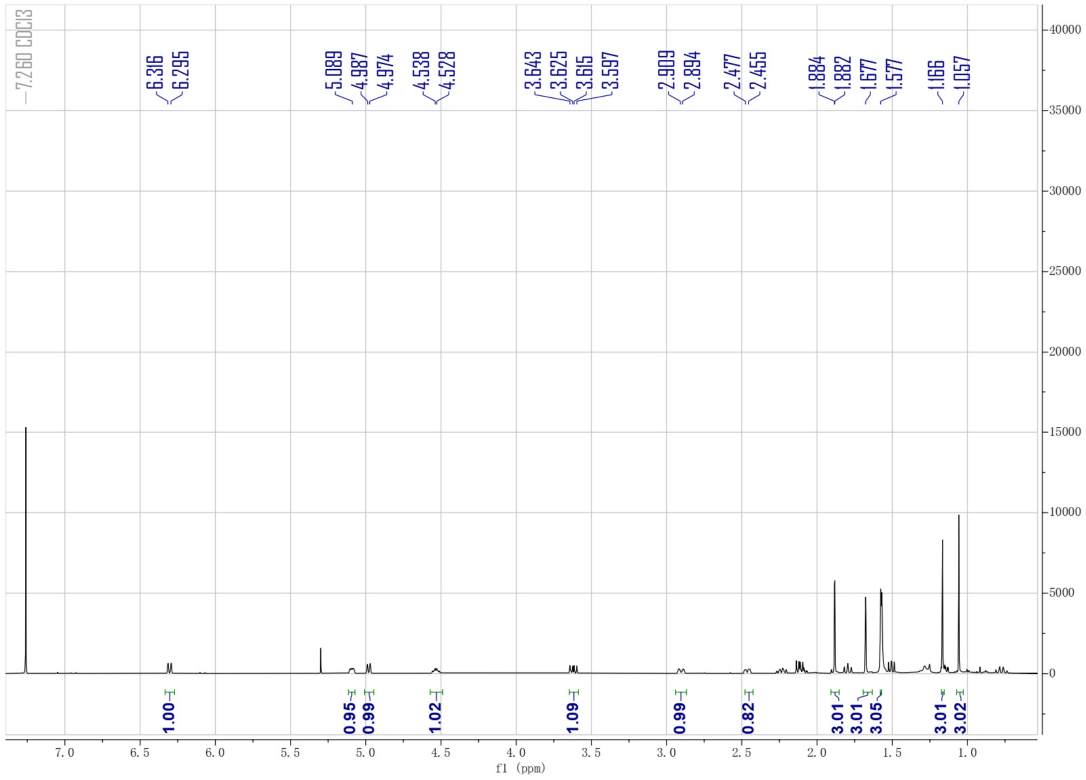

Figure S84. ^{1}H NMR spectrum (500 MHz) of **14** in $CDCl_3$.

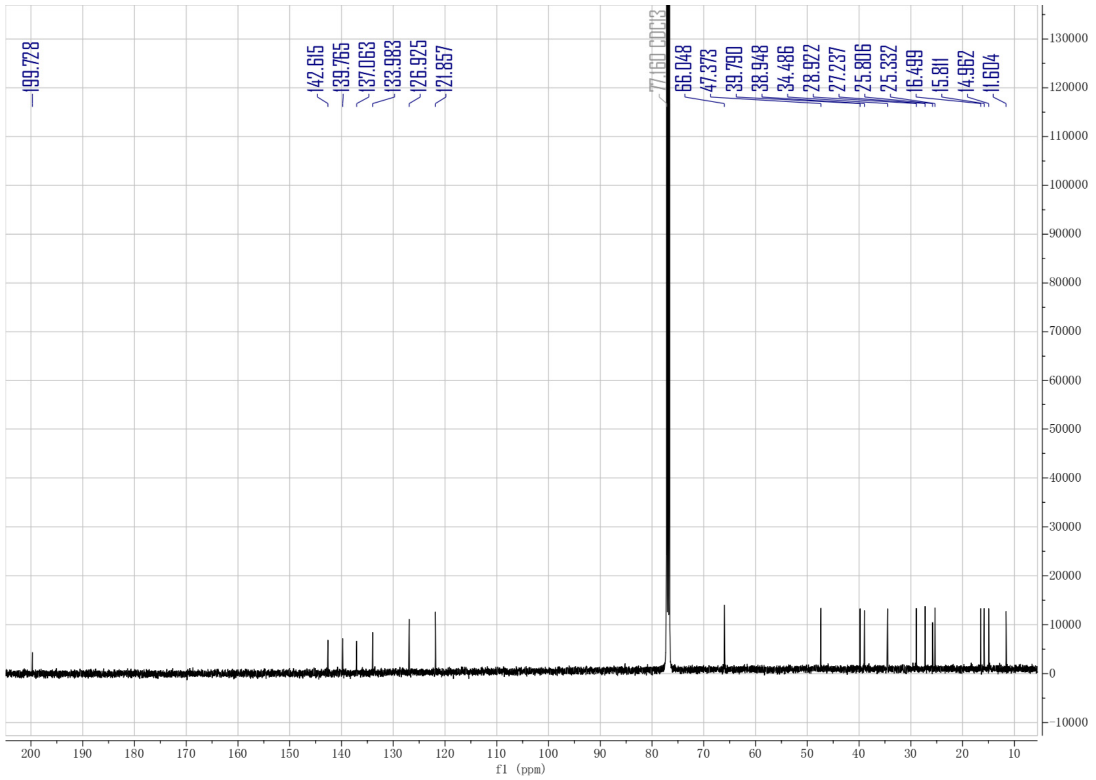

Figure S85. ^{13}C NMR spectrum (125 MHz) of **14** in $CDCl_3$.

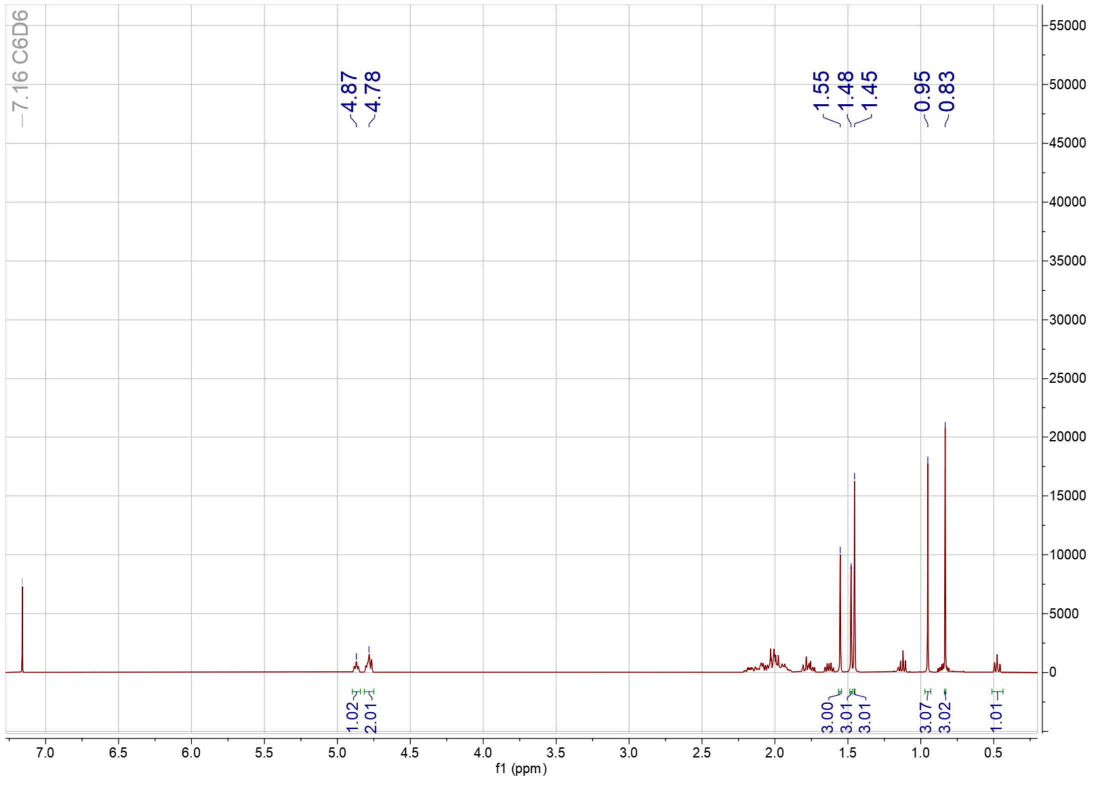

Figure S86. ^{1}H NMR spectrum (500 MHz) of **15** in $CDCl_3$.

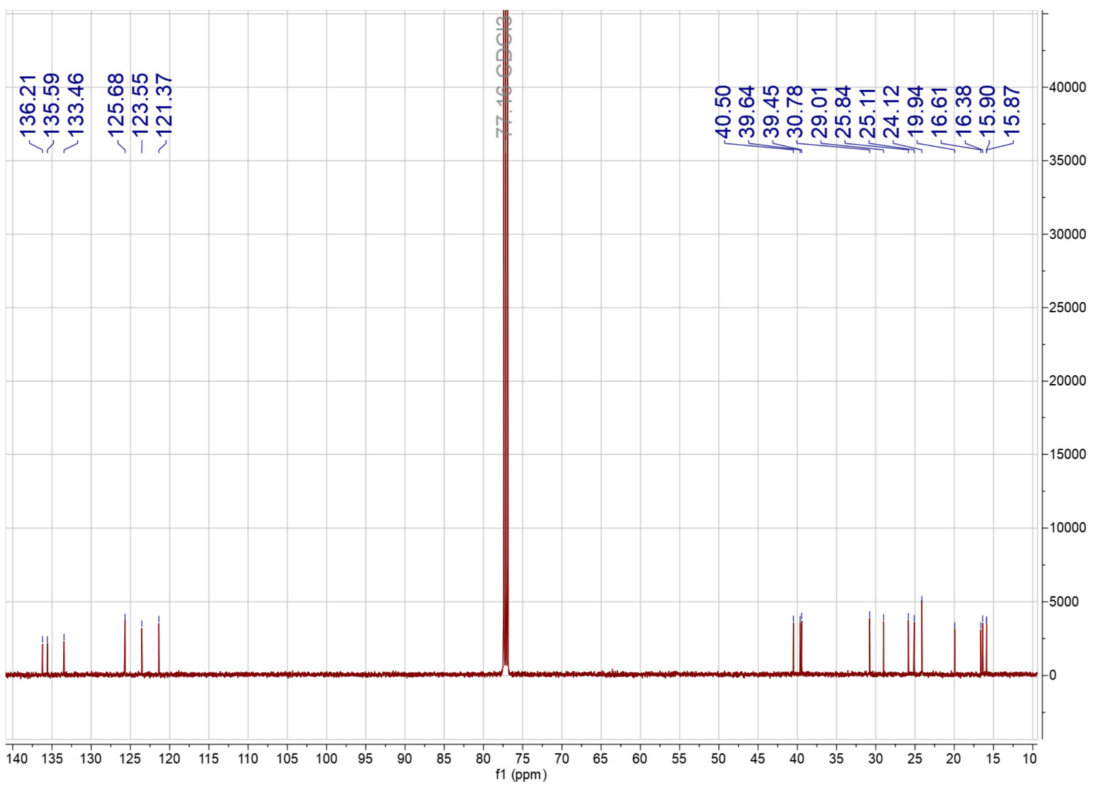

Figure S87. ^{13}C NMR spectrum (125 MHz) of **15** in $CDCl_3$.

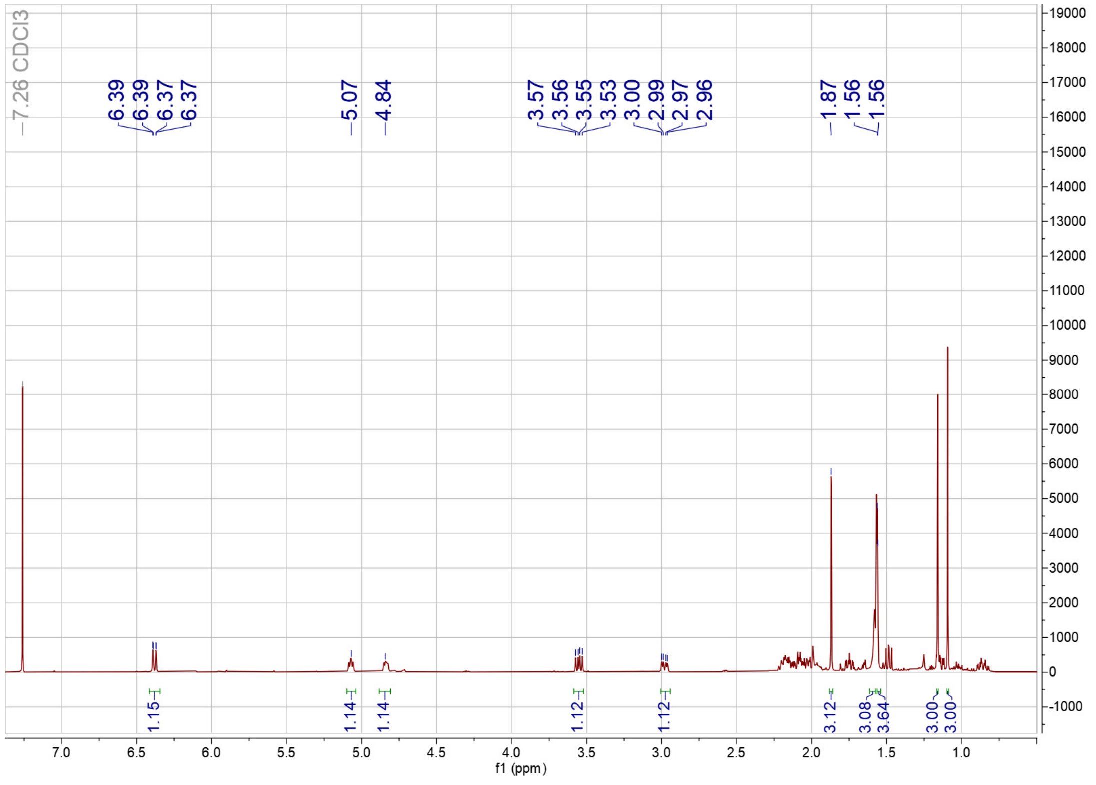

Figure S88. ^{1}H NMR spectrum (500 MHz) of **16** in $CDCl_3$.

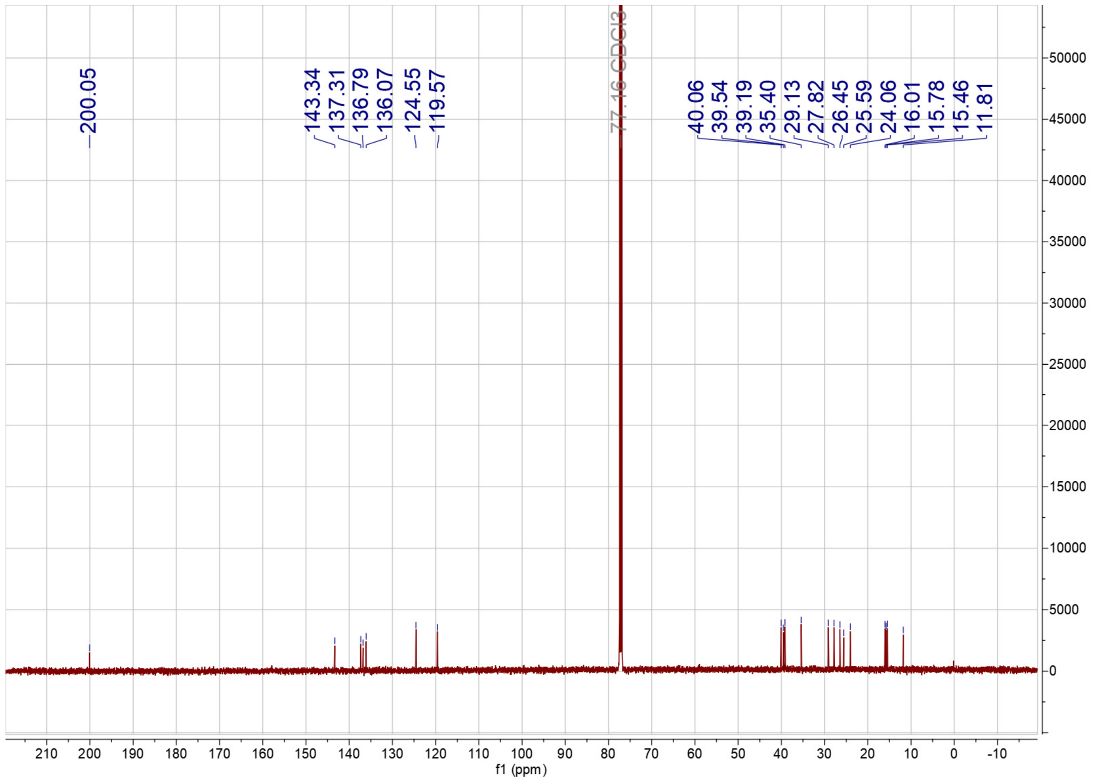

Figure S89. ^{13}C NMR spectrum (125 MHz) of **16** in $CDCl_3$.

10. Raw experimental bioactivity data**.**

Table S6. Raw data of the antitumor activity of the compounds against tumor cell line A549, with doxorubicin (DOX) as the positive control.

Cell viability（%）																
CON	DOX	1	2	3	4	5	6	7	8	9	10	11	12	13	14	16
100.00	17.57	90.17	83.79	101.73	89.63	81.17	73.60	92.94	102.77	106.84	99.80	83.27	76.31	93.10	90.98	98.28
100.00	16.85	94.42	89.89	106.26	101.57	88.40	73.26	102.56	103.79	102.09	102.77	90.81	79.20	91.66	90.14	95.48
100.00	14.67	88.41	91.74	112.73	94.94	91.89	79.79	99.51	105.06	103.53	108.37	92.85	76.23	93.19	87.68	93.87

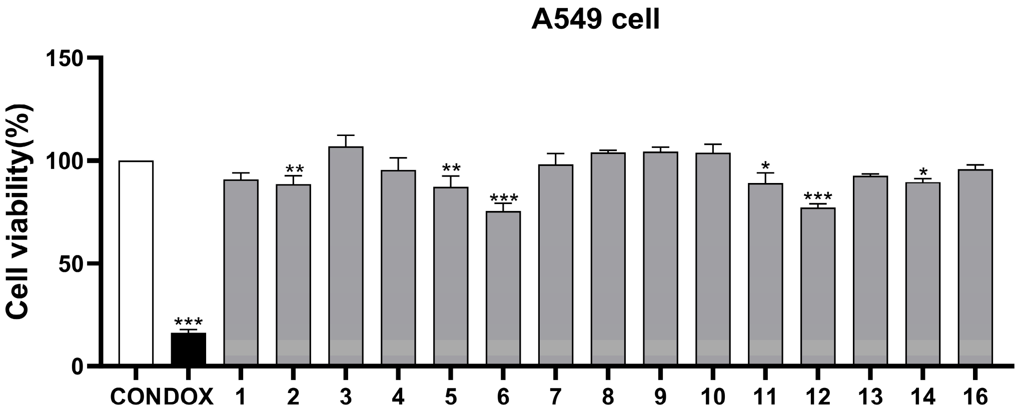

The antiproliferative activity of the series of compounds against A549 tumor cells was evaluated using the MTT assay. The DMSO-treated group (CON) was set as 100% viability, and doxorubicin (DOX) was used as the positive control. Three independent replicates were performed

for each experiment, and the data are presented as mean ± SD. *$p < 0.05$, **$p < 0.01$, ***$p < 0.001$.

Table S7. Raw data of the antitumor activity of the compounds against tumor cell line MV-4-11, with doxorubicin (DOX) as the positive control.

Cell viability（%）																
CON	DOX	1	2	3	4	5	6	7	8	9	10	11	12	13	14	16
100.00	24.66	76.33	93.21	71.73	75.61	77.15	51.92	55.81	51.75	60.14	42.92	53.06	64.60	76.49	79.55	58.83
100.00	24.36	77.35	81.96	70.70	78.27	84.00	55.33	55.34	51.92	60.93	45.45	48.95	65.30	74.13	82.95	63.90
100.00	23.48	78.99	84.82	78.38	76.74	87.59	53.15	55.81	50.79	55.07	47.03	52.19	66.61	82.60	80.24	61.89

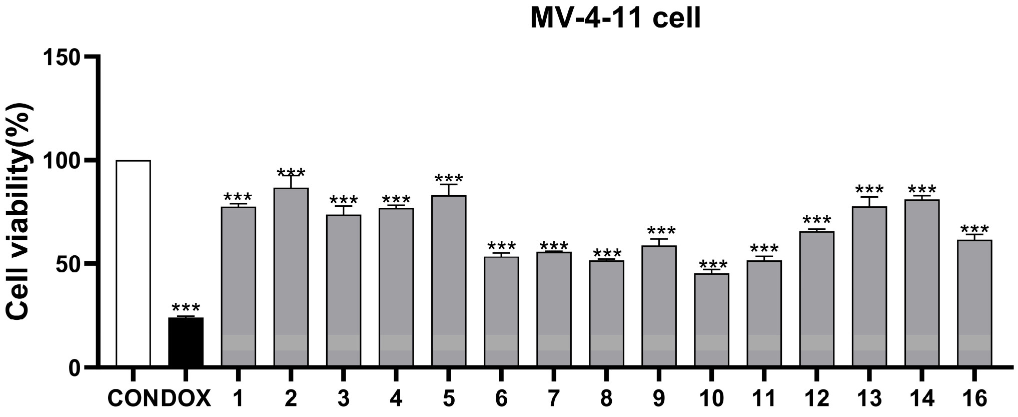

The antiproliferative activity of the series of compounds against MV-4-11 tumor cells was evaluated using the CCK-8 assay. The DMSO-treated group (CON) was set as 100% viability, and doxorubicin (DOX) was used as the positive control. Three independent replicates were performed for each experiment, and the data are presented as mean ± SD. *$p < 0.05$, **$p < 0.01$, ***$p < 0.001$.

Table S8. Raw data of the cytotoxicity activity of the compounds against cell line 293T.

Cell viability（%）															
CON	1	2	3	4	5	6	7	8	9	10	11	12	13	14	16
100.00	97.57	98.62	94.34	97.91	98.32	97.47	98.32	93.39	96.06	90.31	97.81	92.39	95.10	99.39	96.81
100.00	97.24	97.91	93.55	98.74	98.11	97.01	98.11	93.72	96.39	87.52	96.76	91.89	94.93	96.93	97.68
100.00	94.43	97.95	95.22	95.85	97.07	95.06	96.02	95.43	95.47	87.23	96.22	93.18	93.56	97.18	97.39

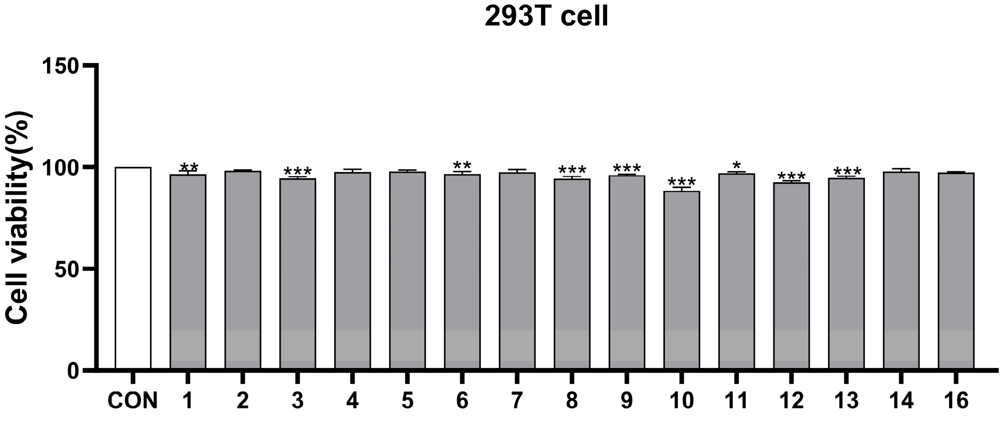

The cytotoxicity of the series of compounds against 293T normal cells was evaluated using the MTT assay. The DMSO-treated group (CON) was set as 100% viability. Three independent replicates were performed for each experiment, and the data are presented as mean ± SD. $*p < 0.05$, $**p < 0.01$, $***p < 0.001$.

Table S9. NO production rate of the compounds in RAW 264.7 macrophages, with dexamethasone (DEX) as the positive control.

NO production rate （%）																	
CON	LPS	DEX	1	2	3	4	5	6	7	8	9	10	11	12	13	14	16
0.00	100.00	41.01	83.70	101.63	82.07	109.78	109.78	62.94	72.28	90.36	75.13	72.08	75.13	94.92	96.45	101.63	84.26
0.00	100.00	41.67	83.70	103.26	80.43	108.15	108.15	59.90	86.96	87.31	75.13	73.60	75.13	93.40	97.97	103.26	81.22
0.00	100.00	38.38	83.70	101.63	90.22	109.78	106.52	64.47	80.43	88.83	75.13	78.17	72.08	91.88	96.45	103.26	85.79

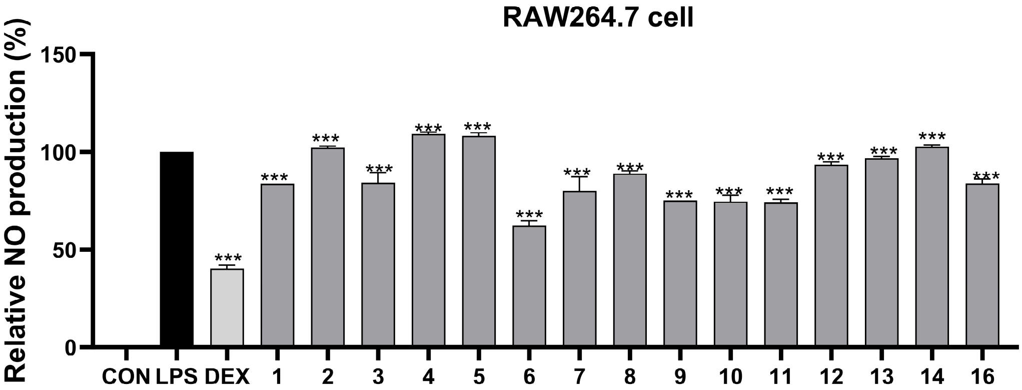

The effect of the test compounds on LPS-induced NO release in RAW 264.7 macrophages was determined using the Griess assay. The relative NO production rate of the LPS-treated model group was set as 100%, and dexamethasone (DEX) was used as the anti-inflammatory positive control. Three independent replicates were performed for each experiment, and the data are presented as mean ± SD. $*p < 0.05$, $**p < 0.01$, $***p < 0.001$.

Table S10. Cytotoxicity data of the compounds against RAW 264.7 cells, with dexamethasone (DEX) as the positive control.

Cell viability （%）																	
CON	LPS	DEX	1	2	3	4	5	6	7	8	9	10	11	12	13	14	16
100.00	101.81	61.91	91.96	94.71	86.72	96.24	92.75	88.86	91.96	47.80	62.30	34.62	44.76	54.39	65.44	68.08	92.36
100.00	98.77	60.34	95.98	99.63	91.38	93.33	89.05	86.33	95.98	49.78	63.16	39.29	47.40	53.99	65.04	65.80	91.30
100.00	93.79	62.08	94.18	97.35	94.81	95.03	88.78	90.23	94.18	50.34	62.86	40.76	52.87	53.79	72.54	68.94	92.21

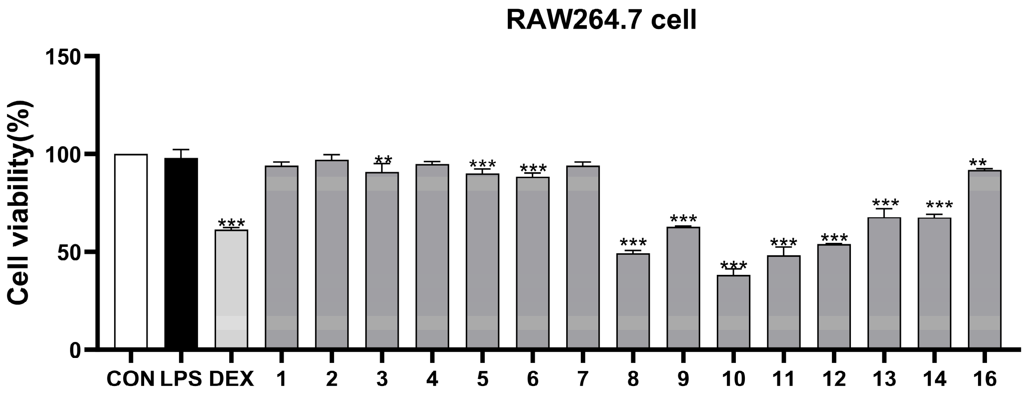

The cytotoxicity of the series of compounds against RAW 264.7 macrophages was evaluated using the MTT assay. The DMSO-treated group (CON) was set as 100% viability. Three independent replicates were performed for each experiment, and the data are presented as mean ± SD. *$p < 0.05$, **$p < 0.01$, ***$p < 0.001$.
